# Method Designed to Respect Molecular Heterogeneity Can Profoundly Correct Present Data Interpretations for Genome-Wide Expression Analysis

**DOI:** 10.1371/journal.pone.0121154

**Published:** 2015-03-20

**Authors:** Chih-Hao Chen, Chueh-Lin Hsu, Shih-Hao Huang, Shih-Yuan Chen, Yi-Lin Hung, Hsiao-Rong Chen, Yu-Chung Wu, Li-Jen Su, H.C. Lee

**Affiliations:** 1 Institute of Systems Biology and Bioinformatics, National Central University, Chungli, Taiwan 32001; 2 Cathay Medical Research Institute, Cathay General Hospital, Taipei, Taiwan 10630; 3 Institute of Bioinformatics and Structural Biology, National Tsing Hua University, Hsinchu, Taiwan 30013; 4 Department of Medicine, Boston University School of Medicine, Boston, MA 02118, United States of America; 5 Division of Thoracic Surgery, Department of Surgery, Taipei Veterans General Hospital, Taipei, Taiwan 11217; 6 School of Medicine, National Yang-Ming University, Taipei, Taiwan 11221; 7 National Center for Theoretical Sciences, Hsinchu, Taiwan 30043; 8 Department of Physics, National Central University, Chungli, Taiwan 32001; 9 Center for Dynamical Biomarkers and Translational Medicine, National Central University, Chungli, Taiwan 32001; Indiana University Bloomington, UNITED STATES

## Abstract

Although genome-wide expression analysis has become a routine tool for gaining insight into molecular mechanisms, extraction of information remains a major challenge. It has been unclear why standard statistical methods, such as the *t*-test and ANOVA, often lead to low levels of reproducibility, how likely applying fold-change cutoffs to enhance reproducibility is to miss key signals, and how adversely using such methods has affected data interpretations. We broadly examined expression data to investigate the reproducibility problem and discovered that molecular heterogeneity, a biological property of genetically different samples, has been improperly handled by the statistical methods. Here we give a mathematical description of the discovery and report the development of a statistical method, named HTA, for better handling molecular heterogeneity. We broadly demonstrate the improved sensitivity and specificity of HTA over the conventional methods and show that using fold-change cutoffs has lost much information. We illustrate the especial usefulness of HTA for heterogeneous diseases, by applying it to existing data sets of schizophrenia, bipolar disorder and Parkinson’s disease, and show it can abundantly and reproducibly uncover disease signatures not previously detectable. Based on 156 biological data sets, we estimate that the methodological issue has affected over 96% of expression studies and that HTA can profoundly correct 86% of the affected data interpretations. The methodological advancement can better facilitate systems understandings of biological processes, render biological inferences that are more reliable than they have hitherto been and engender translational medical applications, such as identifying diagnostic biomarkers and drug prediction, which are more robust.

## Introduction

Genome-wide expression analysis, based on DNA microarray [1] or the more advanced technology of next-generation sequencing [2], has been a mainstay of genomics research. Its application to discover pathways and functions overrepresented in differentially expressed genes (DEGs) between replicated sample cohorts affords biologists an opportunity to gain holistic insight into molecular mechanisms. For selecting DEGs, the *t*-test was the standard method in the earliest years following the introduction of DNA microarray. Although many studies reported success of application, disparities between results obtained by different groups analyzing similar samples were observed [3–9]. In a later study [10], the MicroArray Quality Control Consortium ascribed the disparities to use of the *t*-test and suggested a hybrid method (HM) employing a non-stringent cutoff for the *p*-value from a *t*-test and a fold-change (FC) cutoff because fold-change ranking was found much more reproducible than *t*-test ranking between platforms and test sites [11, 12]. HM has gained popularity ever since, often applied with a greater-than-1 cutoff for fold-change (on log-2 scale) and a cutoff of 0.05 for the *p*-value from a significance test not limited to the *t*-test. To date, HM and and the *t*-test remain the most adopted methods among few alternatives.

Despite the popularity, we remain concerned about their effectiveness because the reproducibility problem has not been appropriately solved. While the poorer reproducibility of the *t*-test signals compromised specificity, possibly due to its blemished approach for variance estimation, little effort has been made to fully clarify the problem so as to formulate a statistical solution. HM enhances reproducibility but lacks statistical control. It may lose signals for two reasons. One is that continuing use of the *t*-test with a loosened cutoff to lessen its impact on specificity is preposterous and has doubtful effect. The other is that arbitrary cutoffs for fold-change are biased towards selecting genes displaying the most pronounced magnitude of differential expression and may neglect biologically significant signals of unemphatic magnitude. For instance, although a metabolic pathway with all member encoding genes displaying a 20% increase can lead to a vastly higher flux than with a single gene displaying a 20-fold increase, it is far less detectable. The weaknesses can leave pathways or functions falsely prioritized and impact data interpretation.

To investigate the reproducibility problem associated with the *t*-test, we examined 156 expression data sets to research data properties violating principles of the *t*-test. We identified as the problem’s primary cause mishandling of molecular heterogeneity, namely the multiplicity of genotypes associated with a phenotype. An accessible example is a patient cohort having two disease subtypes each due to dysfunction of a different pathway. Due to differential expression, the variances of the genes encoding either pathway’s members are wider than average. The variances are mistaken for larger error in a *t*-test, leaving the genes deprioritized and the pathways undetected. The cause has wide impact because most expression studies are based on genetically different samples and that none of currently used methods respect molecular heterogeneity. Increasing sample size won’t help as it cannot improve gene ranking. The impact has been unnoticed for two reasons. One is the lack of a method to appropriately handling molecular heterogeneity. The other is that conventional assessment of methods has been limited to simulation data, which cannot fully account for biological complexity, and spike-in data (e.g. the Affymetrix Latin Square data), which are based on genetically identical samples. Using relevant biological data has been desirable but technically unachievable for the inherent DEGs are not priorly known.

Studies of heterogeneous diseases, such as schizophrenia, bipolar disorder and Parkinson’s disease, are possibly affected the most by the reproducibility problem. Schizophrenia is a psychiatric disorder that alters basic brain processes of perception, emotion and judgment. Bipolar disorder is a psychiatric disorder that manifests in the form of extreme shifts in a person’s mood, energy, and ability to function. Parkinson’s disease is a degenerative neurological disorder characterized by impaired dopaminergic transmission. Although their causes remain elusive, the many genome-wide association studies conducted in recent years, which are mostly collected and meta-analyzed at the SZGene [13], BDGene [14] and PDGene [15] databases, have rendered reliable lists of predisposing genes and have provided formidable insight linking the disease etiologies to genetic background. Sporadic cases of Parkinson’s disease, which represent the vast majority of all diagnosed cases, have been recognized to have a multi-factorial nature. To date only a restricted number of mechanisms are known to contribute to nigral cell death and mitochondrial dysfunction is among the most well-studied. Since the first link between mitochondria and the disease became evident in the early 1980s, a large body of evidence has accrued to confirm that complex I defect plays a central role [16]. Studies examining gene expression profiles in post-mortem human brain samples from patients compared with healthy controls, on the other hand, have only rendered short lists of overall discordant findings [17–32]. Although the disparities raised concerns, they were often attributed to methodological differences in sample preparation, choice of platform, small sample sizes, and lack of control for factors such as age, brain pH and data quality.

In this article, we explain the reproducibility problem in mathematical terms and present a novel method, named Heterogeneity-corrected Transcriptome Analysis (HTA), for appropriately solving it. We also put forth a novel platform, named Biological Measures Of Relative Reliability (BMORR), for assessing methods using any relevant biological data without priorly knowing the inherent DEGs. On BMORR we comprehensively demonstrate the improved reliability of HTA over conventional methods, using 25 data sets for studying schizophrenia, bipolar disorder and Parkinson’s disease, and show the mentioned disparities among previous findings were due to methodological flaws. Based on the 156 data sets, we give an impact assessment of HTA in broadness and profoundness.

## Materials and Methods

### Data collection and data analysis

The data sets for investigating the reproducibility problem (S1 Table) were randomly collected from the Gene Expression Omnibus (GEO) database and were all produced based on the Affymetrix Human Genome U133 Plus 2.0 platform for relevant biological studies. From the data sets 304 contrasts between replicated cohorts were made in the original studies. The data sets for studying the three diseases (S2 Table) were collected from the Stanley Medical Research Institute, the Harvard Brain Tissue Resource Center and the GEO. All data sets were collected in the Affymetrix CEL formats and analyzed using our own developed software.

For the calculations with HTA, we normalized data using scaling normalization, which scales all arrays’ intensities to a global geometric mean; for otherwise calculations, we applied Robust Multi-array Average, which has been the gold standard. We applied no background correction and no control for data quality or confounding factors. We used the Benjamini-Hochberg method [33] to derive false discovery rate (FDR). Our functional analysis was based on the Fisher exact test; the significance level for overrepresentation was *p* < 0.001.

We have also collected from the literature signal lists of the three diseases (S3 Table) for demonstrating our methodological improvements. Note some lists were derived with complicated control for data quality or confounding factors, or through meta-analysis which supposedly improves statistical power.

### An explanation of the reproducibility problem

**Fig 1:**
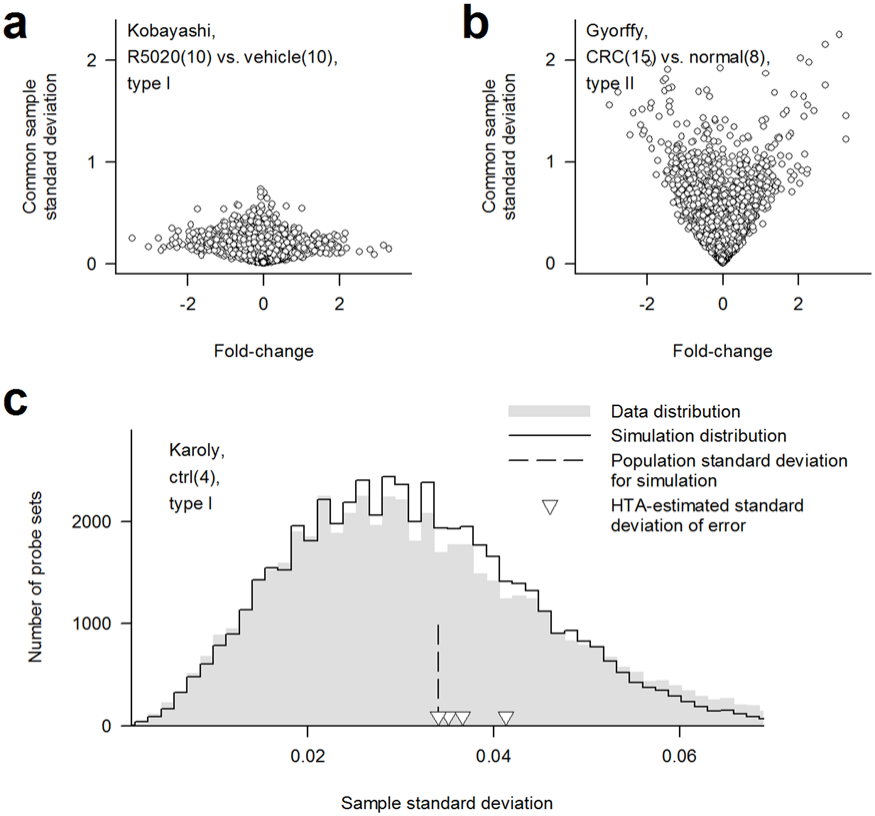
An explanation of the reproducibility problem. **(a)** Probe set scatter plot of a type I contrast showing independence between sample variance and fold-change. **(b)** Probe set scatter plot of a type II contrast showing dependence between sample variance and fold-change. **(c)** Agreement of distribution of sample standard deviation between a type I contrast and a simulated contrast generated using 0.034 as population standard deviation. Also shown for comparison are the samplewise standard deviations of error estimated using HTA. Printed in the panels are the contrasts or the cohort used. Number in parentheses is number of subjects. Common sample standard deviation, a factor of the denominator in the formula for the *t*-statistic, is defined as $\sqrt{\left((n_{1}-1)S_{1}^{2}+(n_{2}-1)S_{2}^{2})/(n_{1}+n_{2}-2\right)}$, where *n*_*i*_ and *S*_*i*_, *i* = 1 or 2, are respectively number of replicates and sample standard deviation of sample cohort *i*.

Of the reproducibility problem, we identified limitations by molecular heterogeneity and by sample size as the primary and secondary causes, respectively. For explanation, we categorize samples as type I or type II and divide variance into error and non-error. Type I samples are genetically identical, while type II samples are not. Of the 304 contrasts, 89 are type I. Error is independent of differential expression, is probabilistic and typically follows a normal distribution. Non-error, as explained below, exists only in type II data, arises from differential expression and should not factor into the significance testing. In a *t*-test, sample variance is the estimator of error variance. The estimator is marred by molecular heterogeneity, which manifests itself in type II data as expansion of non-error with absolute fold-change (Fig. 1a and 1b, see more examples in S1 Fig). Magnitude of the expansion can be pronounced. Although the expansion ostensibly signals variance heterogeneity and justifies variance estimation on a gene-by-gene basis, it results from differential expression and invalidates any method mistaking the affected variances for error and deprioritizing the genes. Increasing sample size won’t help. Accuracy of the estimator is further limited by sample size. We illustrate the impact by comparing data of a type I quadruplicate cohort to four simulated arrays, generated by adding Gaussian noise of the same variance to a common template array, in distribution of sample standard deviations (Fig. 1c). The agreement between the two distributions suggests the probe sets share a common error variance, while the data distribution width reveals how easily *t*-test ranking can be disarranged by chance.

A reasonable solution to factoring non-error out of the significance testing is to assume error variance homogeneity among genes and to estimate the common variances based on data of non-DEGs. Such a solution can also mitigate the sample size limitation because the common variances are estimated based on data of many genes.

### The HTA method

HTA was devised following the above guideline. It takes error variance as homogeneous among genes and, to better handle samples of uneven quality, heterogeneous among replicates. It assumes most genes are non-DEGs and estimates the samplewise error variances based on data of all genes. Accuracy of the estimation allows the significance be tested using *z*-statistics.

**Fig 2:**
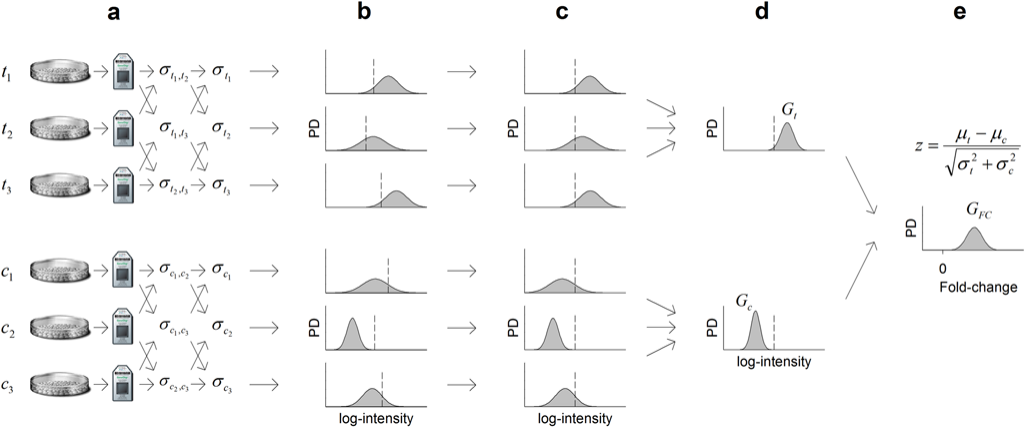
A flow chart of HTA. Here HTA is applied to analyze a gene by contrasting two triplicate cohorts: {*t*_1_,*t*_2_,*t*_3_} vs. {*c*_1_,*c*_2_,*c*_3_}. **(a)** Estimation of samplewise error variances. **(b)** Assigning of a Gaussian to each measurement of log-intensity as the probability density (PD) function of its true value. **(c)** Application of scaling normalization to align the arrays’ log-intensity means, marked with the dashed lines. **(d)** Derivation of the PDF of each cohort’s mean. **(e)** Derivation of the PDF of the true fold-change and calculation of the *z*-statistic for evaluating differential expression of the gene.

HTA is illustrated in Fig. 2, where it evaluates differential expression of a gene between a test cohort {*t*_*i*_∣*i* = 1,2,3} and a control cohort {*c*_*i*_∣*i* = 1,2,3} in the following steps. (i) HTA estimates the samplewise error variances by pairwisely comparing arrays of a cohort (Fig. 2a). The estimation procedure follows. Let {*r*_*i*_∣*i* = 1,…,*n*} be the *n* arrays of a general cohort *r*. HTA calculates the log-intensity difference of each gene between *r*_*i*_ and *r*_*j*_ and then calculates the variance of the differences, which is denoted by $\sigma_{r_{i},r_{j}}^{2}$. Assuming errors are normally distributed, we get $\sigma_{r_{i},r_{j}}^{2}$=$\sigma_{r_{i}}^{2}+\sigma_{r_{j}}^{2}$, where $\sigma_{r_{i}}^{2}$ and $\sigma_{r_{j}}^{2}$ are the samplewise error variances of *r*_*i*_ and *r*_*j*_, respectively. By taking $\sigma_{r_{i},r_{j}}^{2}/2$ as an estimate of $\sigma_{r_{i}}^{2}$ and taking $\sigma_{r_{i}}^{2}$ to be the average of all of its estimates, we get $\sigma_{r_{i}}^{2}$ = $2^{-1}{\left(n-1\right)}^{-1}\underset{j\neq i}{\sum }\sigma_{r_{i},r_{j}}^{2}$. (ii) HTA assigns a Gaussian distribution function (Gaussian) to each measurement of log-intensity (Fig. 2b), taking the measured value as mean and the samplewise error variance as variance, as the probability density function (PDF) of the measurement’s true value. (iii) Following scaling normalization (Fig. 2c), HTA multiplies together the Gaussians of each cohort (Fig. 2d). The resultant Gaussians, *G*_*t*_ = $G(y;\mu_{t},\sigma_{t}^{2})$ and *G*_*c*_=$G(y;\mu_{c},\sigma_{c}^{2})$, are the respective PDFs of the true means of the test and the control cohorts [34]. (iv) The fold-change, the difference between the two true means, can then be predicted using *G*_*FC*_=$G(y;\mu_{t}-\mu_{c},\sigma_{t}^{2}+\sigma_{c}^{2})$ as the PDF. Accordingly, HTA takes $z=(\mu_{t}-\mu_{c})/\sqrt{\sigma_{t}^{2}+\sigma_{c}^{2}}$ to be the *z*-static for evaluating differential expression of the gene (Fig. 2e).

Other than solving both limitations, HTA has the following distinctive features. (i) Its error variance estimation is not susceptible to normalization and is much more accurate than that of the *t*-test (Fig. 1c). (ii) It relies solely on the *z*-test for selecting genes and hence provides complete statistical control. (iii) The samplewise error variances facilitate weighting of samples; when sample quality is even, HTA ranking is same as fold-change ranking; otherwise, the weighting lessens impact from outliers and makes HTA ranking more favorable.

### The BMORR platform

BMORR assesses a method in 3 biological criteria: relative specificity, relative sensitivity and relative reproducibility. The first two are with respect to a single data set. Relative specificity is estimated in number of Gene Ontology functions overrepresented in genes selected under *p* < 0.05, before being divided by that of HTA for normalization. This is because coexpressed genes tend to be functionally coherent but randomly selected genes do not. The *p*-value cutoff ensures the measure is reliably estimated based on sufficient genes even under poor sensitivity. Relative sensitivity is estimated in the product of relative specificity and number of genes selected under a more rigorous cutoff of FDR < 0.05, before being divided by that of HTA for normalization. This is because, under the assumption that relative specificity is proportional to absolute specificity, the product is proportional to number of true positives. Relative reproducibility is with respect to multiple data sets for similar studies and is estimated in average number of times a detected function as described above is repeatedly detected across the data sets, before being divided by that of HTA for normalization.

## Results

### Validation for BMORR

**Fig 3:**
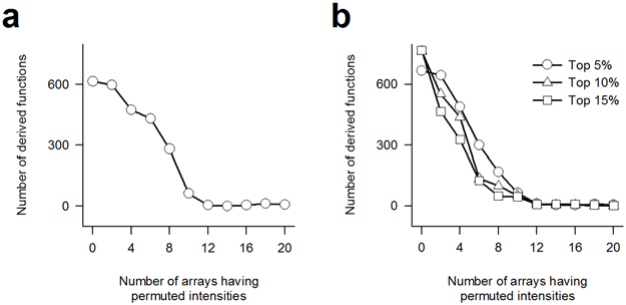
Validation for BMORR. Positive correlation between number of derived functions and specificity demonstrated using **(a)** probe sets selected using the *t*-test(*p* < 0.05) and **(b)** top 5%, 10% and 15% probe sets in *t*-test ranking.

We used the Kobayashi data set [35] to demonstrate the positive correlation between number of derived functions from probe sets selected under *p* < 0.05 and specificity of the probe sets. The type I data set is a contrast between 10 test samples of human mammary epithelial cells treated with R5020 and 10 controls treated with vehicle. From the data set, we first generated 11 replicates and numbered them from 0 to 10. Next, we permuted the intensities of each array of the first *i* test-control pairs of the *i*-th replicate, where *i* = 1–10. We then applied the *t*-test to derive a probe set list from each replicate. The 11 resultant lists supposedly have descending specificities in numerical order. Lastly, we derived functions from the lists and confirmed that they decline with degenerating specificities (Fig. 3a). The decline holds true for top 5%, 10% and 15% probe sets as well (Fig. 3b), indicating the primary cause of the decline is derangement of gene ranking rather than reduction of selected probe sets.

### A survey of methods in use

To assess the scale of impact of the reproducibility problem, we surveyed the original studies of the 156 data sets to estimate the adoption rates of the methods in use (Table 1). Only those of 148 data sets have accessible articles which reveal relevant information. The rates were also separately estimated for the 2006–2009 and 2010–2012 periods to identify potential temporal trends. The resultants show no significant change over time. HM (55%) and the *t*-test (26%) were the most popular. All fold-change cutoffs for HM were greater than 1. The methods were followed by GSEA [36] (11%), ANOVA (9%), SAM (8%) and limma [37] (5%). GSEA, SAM and limma represent earlier efforts to solve the reproducibility problem. GSEA bypasses single gene analysis and evaluates data at the level of gene set, namely a group of genes sharing common biological functions, chromosomal location or location; while SAM and limma moderate *t*-statistics by augmenting variances *ad hoc* to keep variances from becoming too small. Collectively, the 6 methods were adopted by 96% of the studies. None of them recognize the problem of overestimated error.

**Table 1:** Adoption frequencies and rates of data analysis methods.

	2006–2009^1^		2010–2012^2^		Overall	
	Frequency	Rate(%)	Frequency	Rate(%)	Frequency	Rate(%)
*t*-test	22	25	17	28	39	26
ANOVA	7	8	6	10	13	9
limma	5	6	2	3	7	5
SAM	5	6	7	11	12	8
Other tests^3^	4	5	2	3	6	4
HM	49	56	32	52	81	55
GSEA	10	11	6	10	16	11

^1^Based on 87 data sets. Some studies adopted multiple methods.

^2^Based on 61 data sets. Some studies adopted multiple methods.

^3^Including Wilcoxon rank sum test, Mann-Whitney sum rank test, etc.

In the following, we present reliability comparisons of HTA to the above methods except ANOVA. ANOVA is not addressed because it is same as the *t*-test when contrasting two sample cohorts.

### Comprehensive reliability comparisons on the disease data sets

We compared HTA-derived signals from the 25 contrasts for studying the 3 diseases to those derived using the *t*-test, HM(*p* < 0.05,∣*FC*∣ > 1), limma, SAM and GSEA and to those reported in the literature. The significance test for HM was the *t*-test. The signals were first compared in the following 4 aspects if applicable: (i) number of probe sets selected under FDR < 0.05; (ii) number of functions overrepresented in probe sets selected under *p* < 0.05; (iii) occurrence frequency across the data sets of each disease of the derived functions; (iv) capability of detecting functional signatures of the diseases, measured in bias of derived signals towards selected disease-specific functions. HM was applied with the predetermined cutoffs throughout. For schizophrenia and bipolar disorder, the disease-specific functions were neural functions implicated by both the SZGene and the BDGene lists; for Parkinson’s disease, they were mitochondrial functions. For HTA, the *t*-test, HM, limma and SAM, the bias was measured in the *p*-value for overrepresentation; for GSEA, each needed gene set was composed of the platform’s probe sets annotated as relevant, the bounds on size of gene set were removed and the bias was measured using the output nominal *p*-value. The derived biases were benchmarked against those expected by chance, derived for validation with the links between probe sets and functions, or between genes and functions, disordered.

Throughout the diseases, HTA rendered far more probe sets under FDR < 0.05 than the *t*-test, HM, SAM and limma (Panels a and d of S2–S5, S8–S11 and S14–S17 Figs). Regarding overrepresented functions, HTA far outmatched the *t*-test, HM, SAM, limma and the literature-reported signals in number (Panels b and e of S2–S5, S7, S8–S11, S13, S14–S17 and S19 Figs) and in occurrence frequency (Panels c and f of S2–S5, S7, S8–S11, S13, S14–S17 and S19 Figs); HTA also far outmatched the *t*-test, HM, SAM, limma, GSEA and the literature-reported signals in coverage of the disease-specific functions (Panels h and k of S2–S5, S7, S8–S11, S13, S14–S17 and S19 Figs; panels b and e of S6, S12 and S18 Figs). For schizophrenia and bipolar disorder, HTA detected impairments of the neural functions (S2h and S8h Figs), which are overrepresented in the SZGene and BDGene lists (S2g and S8g Figs). For Parkinson’s disease, HTA detected mitochondrial dysfunction and pinpointed downregulation of both complex I and ATP synthase complex (S14h Fig). The problem with complex I is also implicated by the PDGene list (S14g Figs). The findings were reproducible and as significant as *p* = 10^−20^. The *t*-test and HM performed poorly in all aspects (S2, S3, S8, S9, S14 and S15 Figs). SAM and limma rendered slightly more functions than the *t*-test under *p* < 0.05 but zero probe sets under FDR < 0.05 (S4, S5, S10, S11, S16 and S17 Figs), a reasonable result of global variance augmentation which trades off sensitivity for specificity. GSEA exhibited no sensitivity at all for the functions under discussion (S6, S12 and S18 Figs). The results of the literature-reported signals (S7, S13 and S19 Figs) confirmed most of the above findings.

For clearer interpretation, we converted the above results based on BMORR into 4 reliability measures: (i) data set average of relative specificity; (ii) data set average of relative sensitivity; (iii) relative reproducibility; (iv) data set average of detection rate of the disease-specific functions (Table 2). The results show HTA is remarkably superior in all measures, particularly in sensitivity.

**Table 2:** Comprehensive reliability comparisons of HTA to conventional methods and literature-reported signals.

	Schizophrenia				Bipolar disorder				Parkinson’s disease			
	RSP^1^	RSE^2^	RR^3^	DRF^4^	RSP^1^	RSE^2^	RR^3^	DRF^4^	RSP^1^	RSE^2^	RR^3^	DRF^4^
HTA	1.00	1.00	1.00	0.60	1.00	1.00	1.00	0.45	1.00	1.00	1.00	0.66
*t*-test	0.11	0.00	0.05	0.01	0.12	0.00	0.22	0.00	0.19	0.00	0.14	0.21
HM	0.01	0.00	0.00	0.00	0.00	0.00	0.00	0.00	0.14	0.00	0.11	0.04
SAM	0.27	0.00	0.25	0.07	0.22	0.00	0.32	0.05	0.33	0.00	0.25	0.29
limma	0.15	0.00	0.07	0.03	0.14	0.00	0.19	0.03	0.29	0.00	0.25	0.27
GSEA	NA	NA	NA	0.00	NA	NA	NA	0.00	NA	NA	NA	0.00
LRS^5^	NA	NA	NA	0.02	NA	NA	NA	0.09	NA	NA	NA	0.07

^1^Average relative specificity compared to HTA.

^2^Average relative sensitivity compared to HTA.

^3^Relative reproducibility compared to HTA.

^4^Average detection rate of the disease-specific functions.

^5^Literature-reported signals.

The overall results show the disparities among the previous findings were due to methodological flaws and that a large body of disease information has been overlooked. They also reveal that using the conventional methods rendered the efforts to control for data quality and confounding factors, or to perform meta-analysis, futile.

Because we saw no apparent advantage of SAM, limma and GSEA over the *t*-test, we excluded them from further comparisons.

### Broad reliability comparisons and HTA impact assessment

To see if the above results hold true for general studies, we repeated most of the above calculations on the 304 contrasts comparing HTA to the *t*-test and HM (S20–S22 Figs). Quantitatively, contrast averages of relative specificity and relative sensitivity were respectively 0.36 and 0.20 for the *t*-test, and respectively 0.44 and 0.02 for HM. HTA bettered the *t*-test in relative specificity and relative sensitivity respectively on 98% and 97% of the contrasts, and bettered HM respectively on 89% and 99%. Overall, HTA rendered remarkably improved specificity and sensitivity. The inferior sensitivity of HM revealed ∣*FC*∣ > 1 is too stringent, even though it is softer than conventionally chosen.

**Fig 4:**
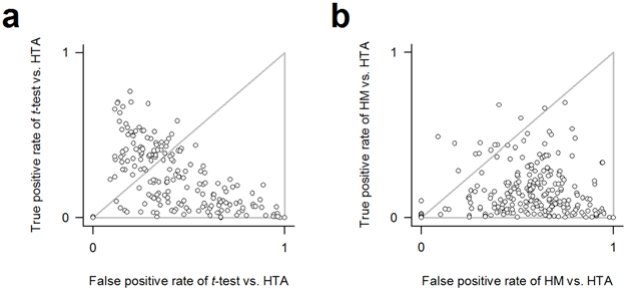
An impact assessment of HTA. In reference to HTA-derived functions from each of the 304 contrasts, we calculated the receiver operating characteristics of **(a)** *t*-test-derived functions and **(b)** HM-derived functions and evaluated the AUC accordingly. The grey triangle in an ROC space encloses the area of AUC < 0.5.

The broad sensitivity improvement of HTA piqued our curiosity about the amount of lost information in existing data. We estimated that as follows using the 304 contrast. We quantified the content difference between functions derived from a contrast using either the *t*-test(FDR < 0.05) or HM(*p* < 0.05,∣*FC*∣ > 1) and those derived using HTA(FDR < 0.05) in area under receiver operating curve (AUC), evaluated taking the latter functions as reference, and considered the contrast has been misanalyzed if AUC < 0.5. Respectively for the *t*-test and HM, we found 74% and 91% of the 304 contrasts have been misanalyzed (Fig. 4). Taking into account the methods’ respective adoption rates of 26% and 55%, we conclude HTA can profoundly correct 86% of the affected data interpretations. Note AUC is independent of choice of reference.

## Discussion

We have performed the first study to clarify, solve and broadly appraise the reproducibility problem of genome-wide expression analysis. Our work was based on a total of 328 cohort contrasts derived from 180 data sets produced for relevant biological studies. We present HTA as a solution, elucidate why its simple but rigorous design can solve the two fundamental causes of the problem and demonstrate its improved reliability, comprehensibly and broadly. The demonstration is facilitated by BMORR, a novel platform designed to assess methods using any biological data so that biological complexity, such as molecular heterogeneity, can be taken into account. Using HTA and BMORR we show the problem has affected over 96% of expression studies and that 86% of the affected data interpretations can be profoundly corrected.

HTA is demonstrated on raw data with the simplest normalization strategy, no background correction and no control for data quality or confounding factors. The remarkably improved reliability indicates that mishandling of molecular heterogeneity has been the bottleneck confining the breadth of biomedical research hypotheses explorable and warrants a paradigm shift in future method design. Although the data for the demonstrations were generated using microarrays, molecular heterogeneity as a biological property will equally necessitate HTA, or similarly designed methods, whatever technology is adopted, including next-generation sequencing.

The improved reliability of HTA can benefit a wide spectrum of research fields, ranging from basic biology to the pharmaceutical industry, where it can render inferences that are more reliable than they have hitherto been and engender translational medical applications, such as identifying diagnostic biomarkers and predicting drugs, that are more robust. We also expect HTA to represent an excellent opportunity to rediscover the large body of existing data having been accumulating at public repositories since the introduction of DNA microarray.

## Supporting Information

S1 Fig Manifestation of molecular heterogeneity in expression data. Variance appears independent of fold-change for the type I contrasts on the left; while for the type II contrasts on the right, it tends to expand with absolute fold-change. The contrasts used are as printed. Number in parentheses is number of subjects. Common sample standard deviation, a factor in the denominator of the formula for the *t*-statistic, is defined as $\sqrt{\left(\left(n_{1}-1\right)S_{1}^{2}+\left(n_{2}-1\right)S_{2}^{2}\right)/\left(n_{1}+n_{2}-2\right)}$, where *n*_*i*_ and *S*_*i*_, *i* = 1 or 2, are respectively number of replicates and sample standard deviation of sample cohort *i*.
(TIF)

S2 Fig Superior reliability of HTA over the *t*-test on the schizophrenia data sets. **(a)** Numbers of HTA-derived probe sets. **(b)** Numbers of HTA-derived functions. **(c)** Occurrence frequency distributions of HTA-derived functions. **(d)** Numbers of *t*-test-derived probe sets. **(e)** Numbers of *t*-test-derived functions. **(f)** Occurrence frequency distributions of *t*-test-derived functions. **(g)** Bias of the SZGene list towards the neural functions. **(h)** Biases of HTA-derived probe sets towards the neural functions. **(i)** The biases in (h) expected by chance. **(j)** Biases of *t*-test-derived probe sets towards the neural functions. **(k)** The biases in (j) expected by chance.
(TIF)

S3 Fig Superior reliability of HTA over HM on the schizophrenia data sets. **(a)** Numbers of HTA-derived probe sets. **(b)** Numbers of HTA-derived functions. **(c)** Occurrence frequency distributions of HTA-derived functions. **(d)** Numbers of HM-derived probe sets. **(e)** Numbers of HM-derived functions. **(f)** Occurrence frequency distributions of HM-derived functions. **(g)** Bias of the SZGene list towards the neural functions. **(h)** Biases of HTA-derived probe sets towards the neural functions. **(i)** The biases in (h) expected by chance. **(j)** Biases of HM-derived probe sets towards the neural functions. **(k)** The biases in (j) expected by chance.
(TIF)

S4 Fig Superior reliability of HTA over SAM on the schizophrenia data sets. **(a)** Numbers of HTA-derived probe sets. **(b)** Numbers of HTA-derived functions. **(c)** Occurrence frequency distributions of HTA-derived functions. **(d)** Numbers of SAM-derived probe sets. **(e)** Numbers of SAM-derived functions. **(f)** Occurrence frequency distributions of SAM-derived functions. **(g)** Bias of the SZGene list towards the neural functions. **(h)** Biases of HTA-derived probe sets towards the neural functions. **(i)** The biases in (h) expected by chance. **(j)** Biases of SAM-derived probe sets towards the neural functions. **(k)** The biases in (j) expected by chance.
(TIF)

S5 Fig Superior reliability of HTA over limma on the schizophrenia data sets. **(a)** Numbers of HTA-derived probe sets. **(b)** Numbers of HTA-derived functions. **(c)** Occurrence frequency distributions of HTA-derived functions. **(d)** Numbers of limma-derived probe sets. **(e)** Numbers of limma-derived functions. **(f)** Occurrence frequency distributions of limma-derived functions. **(g)** Bias of the SZGene list towards the neural functions. **(h)** Biases of HTA-derived probe sets towards the neural functions. **(i)** The biases in (h) expected by chance. **(j)** Biases of limma-derived probe sets towards the neural functions. **(k)** The biases in (j) expected by chance.
(TIF)

S6 Fig Superior reliability of HTA over GSEA on the schizophrenia data sets. **(a)** Bias of the SZGene list towards the neural functions. **(b)** Biases of HTA-derived probe sets towards the neural functions. **(c)** The biases in (b) expected by chance. **(d)** GSEA-derived biases towards the neural functions. **(e)** The biases in (d) expected by chance.
(TIF)

S7 Fig Superiority of HTA-derived schizophrenia signals over the literature-reported ones. **(a)** Numbers of HTA-derived probe sets. **(b)** Numbers of HTA-derived functions. **(c)** Occurrence frequency distributions of HTA-derived functions. **(d)** Numbers of the literature-reported signals. **(e)** Numbers of functions derived from the literature-reported signals. **(f)** Occurrence frequency distributions of the functions in (e). **(g)** Bias of the SZGene list towards the neural functions. **(h)** Biases of HTA-derived signals towards the neural functions. **(i)** The biases in (h) expected by chance. **(j)** Biases of the literature-reported signals towards the neural functions. **(k)** The biases in (j) derived by chance.
(TIF)

S8 Fig Superior reliability of HTA over the *t*-test on the bipolar disorder data sets. **(a)** Numbers of HTA-derived probe sets. **(b)** Numbers of HTA-derived functions. **(c)** Occurrence frequency distributions of HTA-derived functions. **(d)** Numbers of *t*-test-derived probe sets. **(e)** Numbers of *t*-test-derived functions. **(f)** Occurrence frequency distributions of *t*-test-derived functions. **(g)** Bias of the BDGene list towards the neural functions. **(h)** Biases of HTA-derived probe sets towards the neural functions. **(i)** The biases in (h) expected by chance. **(j)** Biases of *t*-test-derived probe sets towards the neural functions. **(k)** The biases in (j) expected by chance.
(TIF)

S9 Fig Superior reliability of HTA over HM on the bipolar disorder data sets. **(a)** Numbers of HTA-derived probe sets. **(b)** Numbers of HTA-derived functions. **(c)** Occurrence frequency distributions of HTA-derived functions. **(d)** Numbers of HM-derived probe sets. **(e)** Numbers of HM-derived functions. **(f)** Occurrence frequency distributions of HM-derived functions. **(g)** Bias of the BDGene list towards the neural functions. **(h)** Biases of HTA-derived probe sets towards the neural functions. **(i)** The biases in (h) expected by chance. **(j)** Biases of HM-derived probe sets towards the neural functions. **(k)** The biases in (j) expected by chance.
(TIF)

S10 Fig Superior reliability of HTA over SAM on the bipolar disorder data sets. **(a)** Numbers of HTA-derived probe sets. **(b)** Numbers of HTA-derived functions. **(c)** Occurrence frequency distributions of HTA-derived functions. **(d)** Numbers of SAM-derived probe sets. **(e)** Numbers of SAM-derived functions. **(f)** Occurrence frequency distributions of SAM-derived functions. **(g)** Bias of the BDGene list towards the neural functions. **(h)** Biases of HTA-derived probe sets towards the neural functions. **(i)** The biases in (h) expected by chance. **(j)** Biases of SAM-derived probe sets towards the neural functions. **(k)** The biases in (j) expected by chance.
(TIF)

S11 Fig Superior reliability of HTA over limma on the bipolar disorder data sets. **(a)** Numbers of HTA-derived probe sets. **(b)** Numbers of HTA-derived functions. **(c)** Occurrence frequency distributions of HTA-derived functions. **(d)** Numbers of limma-derived probe sets. **(e)** Numbers of limma-derived functions. **(f)** Occurrence frequency distributions of limma-derived functions. **(g)** Bias of the BDGene list towards the neural functions. **(h)** Biases of HTA-derived probe sets towards the neural functions. **(i)** The biases in (h) expected by chance. **(j)** Biases of limma-derived probe sets towards the neural functions. **(k)** The biases in (j) expected by chance.
(TIF)

S12 Fig Superior reliability of HTA over GSEA on the bipolar disorder data sets. **(a)** Bias of the BDGene list towards the neural functions. **(b)** Biases of HTA-derived probe sets towards the neural functions. **(c)** The biases in (b) expected by chance. **(d)** GSEA-derived biases towards the neural functions. **(e)** The biases in (d) expected by chance.
(TIF)

S13 Fig Superiority of HTA-derived bipolar disorder signals over the literature-reported ones. **(a)** Numbers of HTA-derived probe sets. **(b)** Numbers of HTA-derived functions. **(c)** Occurrence frequency distributions of HTA-derived functions. **(d)** Numbers of the literature-reported signals. **(e)** Numbers of functions derived from the literature-reported signals. **(f)** Occurrence frequency distributions of the functions in (e). **(g)** Bias of the BDGene list towards the neural functions. **(h)** Biases of HTA-derived signals towards the neural functions. **(i)** The biases in (h) expected by chance. **(j)** Biases of the literature-reported signals towards the neural functions. **(k)** The biases in (j) derived by chance.
(TIF)

S14 Fig Superior reliability of HTA over the *t*-test on the Parkinson’s disease data sets. **(a)** Numbers of HTA-derived probe sets. **(b)** Numbers of HTA-derived functions. **(c)** Occurrence frequency distributions of HTA-derived functions. **(d)** Numbers of *t*-test-derived probe sets. **(e)** Numbers of *t*-test-derived functions. **(f)** Occurrence frequency distributions of *t*-test-derived functions. **(g)** Bias of the PDGene list towards the mitochondrial functions. **(h)** Biases of HTA-derived probe sets towards the mitochondrial functions. **(i)** The biases in (h) expected by chance. **(j)** Biases of *t*-test-derived probe sets towards the mitochondrial functions. **(k)** The biases in (j) expected by chance.
(TIF)

S15 Fig Superior reliability of HTA over HM on the Parkinson’s disease data sets. **(a)** Numbers of HTA-derived probe sets. **(b)** Numbers of HTA-derived functions. **(c)** Occurrence frequency distributions of HTA-derived functions. **(d)** Numbers of HM-derived probe sets. **(e)** Numbers of HM-derived functions. **(f)** Occurrence frequency distributions of HM-derived functions. **(g)** Bias of the PDGene list towards the mitochondrial functions. **(h)** Biases of HTA-derived probe sets towards the mitochondrial functions. **(i)** The biases in (h) expected by chance. **(j)** Biases of HM-derived probe sets towards the mitochondrial functions. **(k)** The biases in (j) expected by chance.
(TIF)

S16 Fig Superior reliability of HTA over SAM on the Parkinson’s disease data sets. **(a)** Numbers of HTA-derived probe sets. **(b)** Numbers of HTA-derived functions. **(c)** Occurrence frequency distributions of HTA-derived functions. **(d)** Numbers of SAM-derived probe sets. **(e)** Numbers of SAM-derived functions. **(f)** Occurrence frequency distributions of SAM-derived functions. **(g)** Bias of the PDGene list towards the mitochondrial functions. **(h)** Biases of HTA-derived probe sets towards the mitochondrial functions. **(i)** The biases in (h) expected by chance. **(j)** Biases of SAM-derived probe sets towards the mitochondrial functions. **(k)** The biases in (j) expected by chance.
(TIF)

S17 Fig Superior reliability of HTA over limma on the Parkinson’s disease data sets. **(a)** Numbers of HTA-derived probe sets. **(b)** Numbers of HTA-derived functions. **(c)** Occurrence frequency distributions of HTA-derived functions. **(d)** Numbers of limma-derived probe sets. **(e)** Numbers of limma-derived functions. **(f)** Occurrence frequency distributions of limma-derived functions. **(g)** Bias of the PDGene list towards the mitochondrial functions. **(h)** Biases of HTA-derived probe sets towards the mitochondrial functions. **(i)** The biases in (h) expected by chance. **(j)** Biases of limma-derived probe sets towards the mitochondrial functions. **(k)** The biases in (j) expected by chance.
(TIF)

S18 Fig Superior reliability of HTA over GSEA on the Parkinson’s disease data sets. **(a)** Bias of the PDGene list towards the mitochondrial functions. **(b)** Biases of HTA-derived probe sets towards the mitochondrial functions. **(c)** The biases in (b) expected by chance. **(d)** GSEA-derived biases towards the mitochondrial functions. **(e)** The biases in (d) expected by chance.
(TIF)

S19 Fig Superiority of HTA-derived Parkinson’s disease signals over the literature-reported ones. **(a)** Numbers of HTA-derived probe sets. **(b)** Numbers of HTA-derived functions. **(c)** Occurrence frequency distributions of HTA-derived functions. **(d)** Numbers of the literature-reported signals. **(e)** Numbers of functions derived from the literature-reported signals. **(f)** Occurrence frequency distributions of the functions in (e). **(g)** Bias of the PDGene list towards the mitochondrial functions. **(h)** Biases of HTA-derived signals towards the mitochondrial functions. **(i)** The biases in (h) expected by chance. **(j)** Biases of the literature-reported signals towards the mitochondrial functions. **(k)** The biases in (j) expected by chance.
(TIF)

S20 Fig Numbers of probe sets and functions derived using HTA(*p* < 0.05), *t*-test(*p* < 0.05) and HM(*p* < 0.05,∣*FC*∣ > 1) from the 304 contrasts. Number in parentheses is number of subjects. **(a,c,e,g)** Numbers of probe sets. **(b,d,f,h)** Numbers of functions.
(TIF)

S21 Fig Numbers of probe sets and functions derived using HTA(FDR < 0.05), *t*-test(FDR < 0.05) and HM(*p* < 0.05,∣*FC*∣ > 1) from the 304 contrasts. Number in parentheses is number of subjects. **(a,c,e,g)** Numbers of probe sets. **(b,d,f,h)** Numbers of functions.
(TIF)

S22 Fig Superior specificity and sensitivity of HTA over the *t*-test and HM. **The 304 contrasts were used. Number in parentheses is number of subjects. (a,c,e,g)** Relative sensitivity of the methods. **(b,d,f,h)** Relative specificity of the methods.
(TIF)

S1 Table The 156 data sets and the 304 contrasts. (PDF)

S2 Table The schizophrenia, bipolar disorder and Parkinson’s disease data sets. (PDF)

S3 Table The literature-reported signals of schizophrenia, bipolar disorder and Parkinson’s disease. (PDF)

S1 Text The URLs for downloading the used data sets. (PDF)
