# Supplementary figures and images for: Method Designed to Respect Molecular Heterogeneity Can Profoundly Correct Present Data Interpretations for Genome-Wide Expression Analysis
Source: PLoS One. 2015 Mar 20;10(3):e0121154. doi: 10.1371/journal.pone.0121154 (PMC4368820; doi:10.1371/journal.pone.0121154)

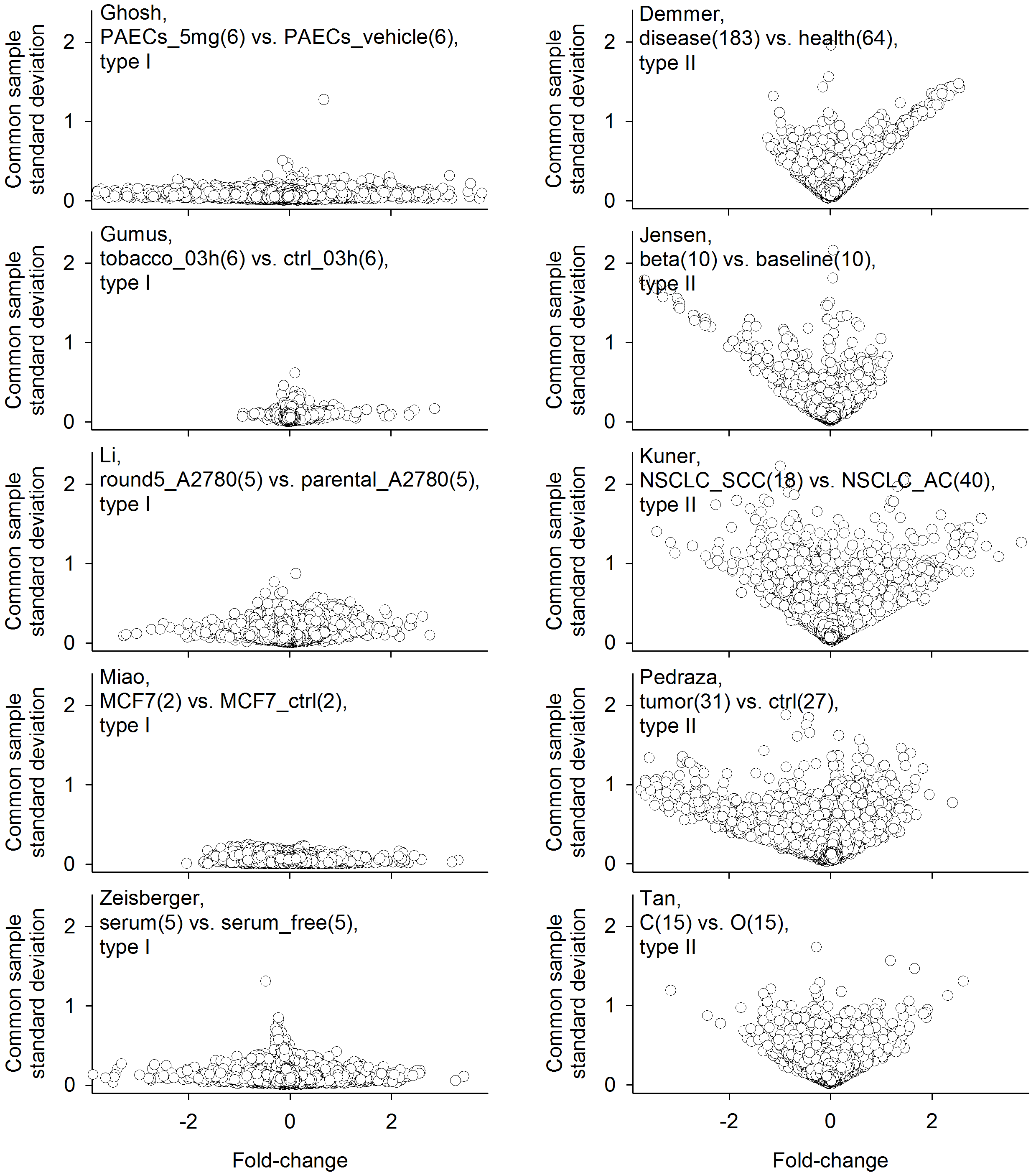

Supplement: S1 Fig — Variance appears independent of fold-change for the type I contrasts on the left; while for the type II contrasts on the right, it tends to expand with absolute fold-change. The contrasts used are as printed. Number in parentheses is number of subjects. Common sample standard deviation, a factor in the denominator of the formula for the t-statistic, is defined as ((n1−1)S12+(n2−1)S22)/(n1+n2−2), where n i and S i, i = 1 or 2, are respectively number of replicates and sample standard deviation of sample cohort i. (TIF) [file pone.0121154.s001.TIF]

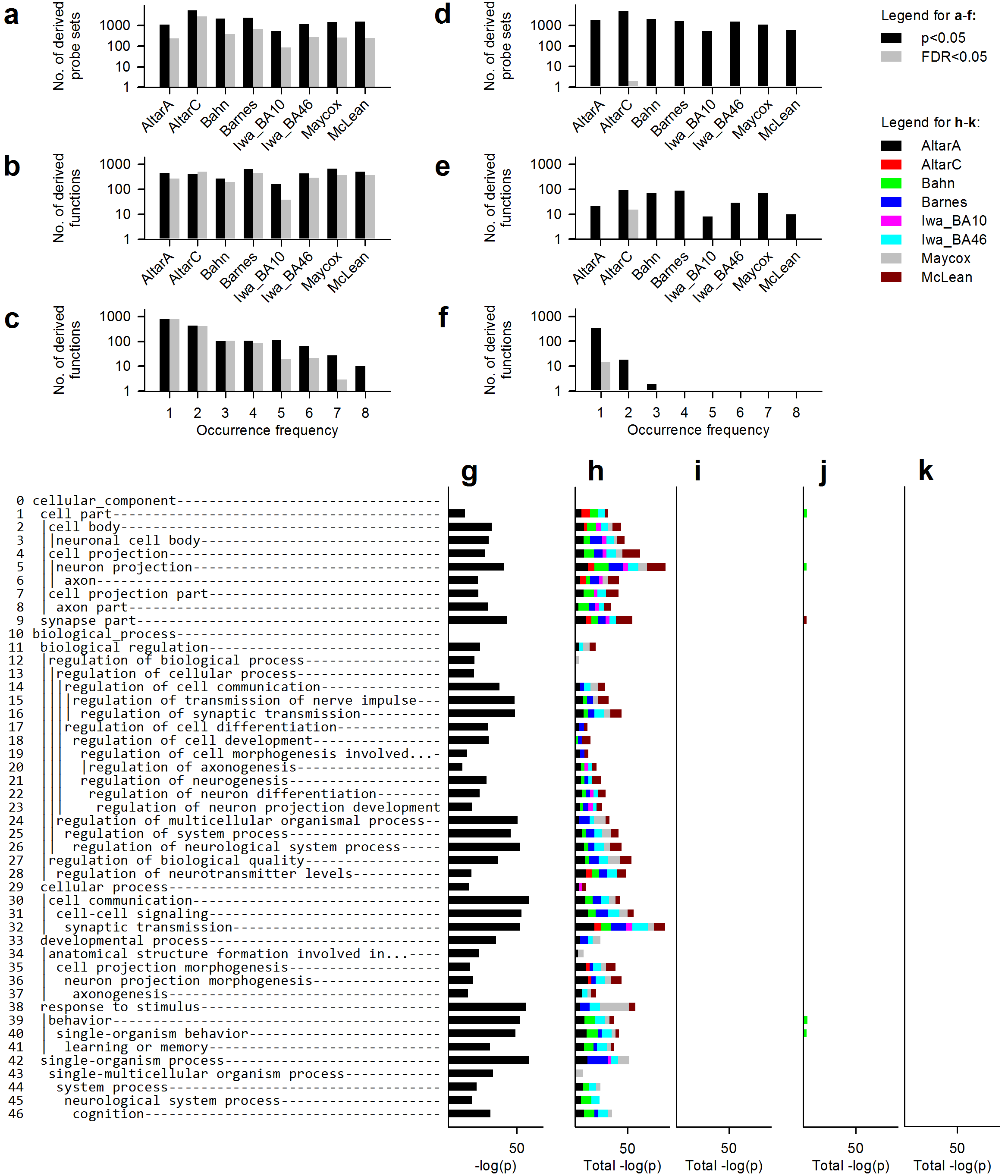

Supplement: S2 Fig — (a) Numbers of HTA-derived probe sets. (b) Numbers of HTA-derived functions. (c) Occurrence frequency distributions of HTA-derived functions. (d) Numbers of t-test-derived probe sets. (e) Numbers of t-test-derived functions. (f) Occurrence frequency distributions of t-test-derived functions. (g) Bias of the SZGene list towards the neural functions. (h) Biases of HTA-derived probe sets towards the neural functions. (i) The biases in (h) expected by chance. (j) Biases of t-test-derived probe sets towards the neural functions. (k) The biases in (j) expected by chance. (TIF) [file pone.0121154.s002.TIF]

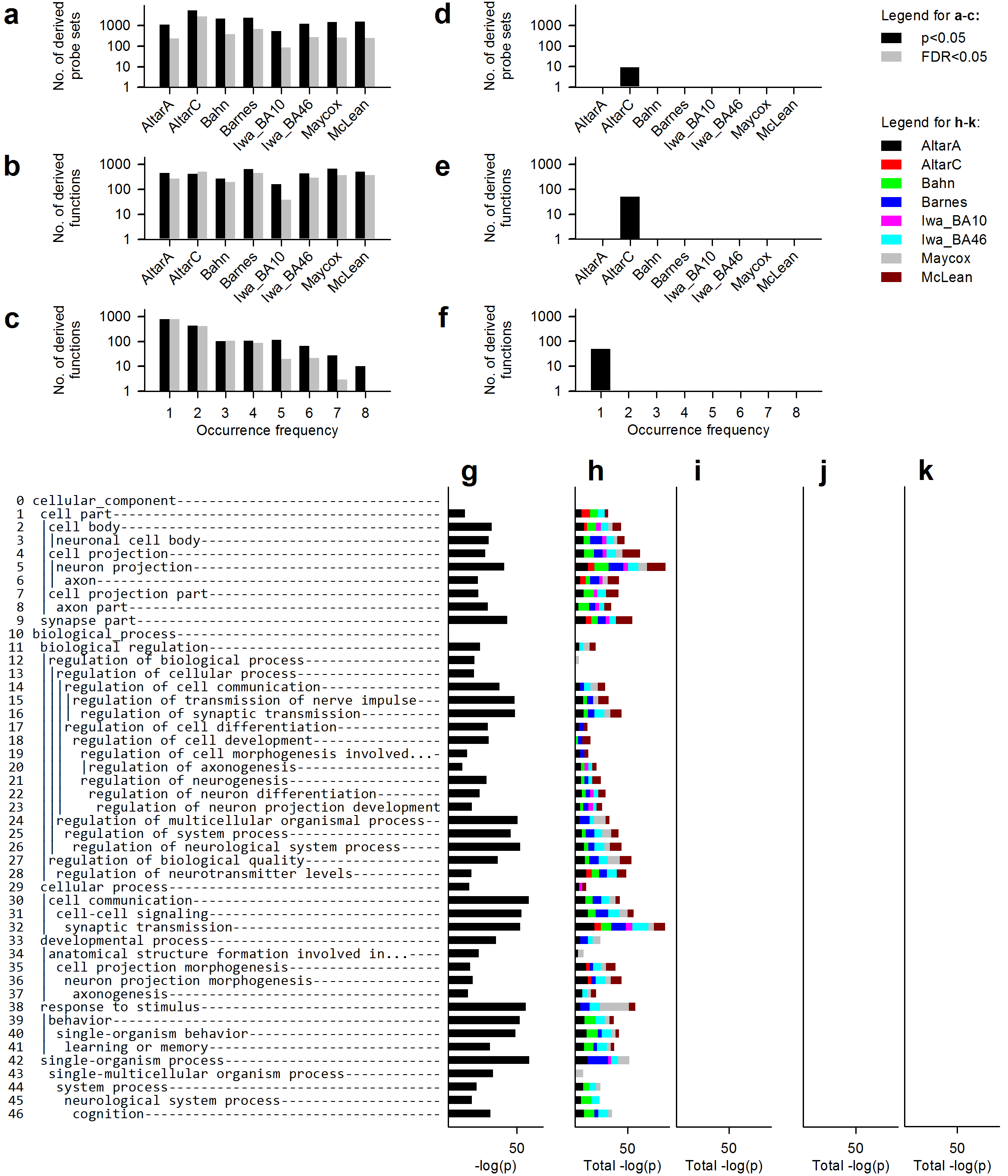

Supplement: S3 Fig — (a) Numbers of HTA-derived probe sets. (b) Numbers of HTA-derived functions. (c) Occurrence frequency distributions of HTA-derived functions. (d) Numbers of HM-derived probe sets. (e) Numbers of HM-derived functions. (f) Occurrence frequency distributions of HM-derived functions. (g) Bias of the SZGene list towards the neural functions. (h) Biases of HTA-derived probe sets towards the neural functions. (i) The biases in (h) expected by chance. (j) Biases of HM-derived probe sets towards the neural functions. (k) The biases in (j) expected by chance. (TIF) [file pone.0121154.s003.TIF]

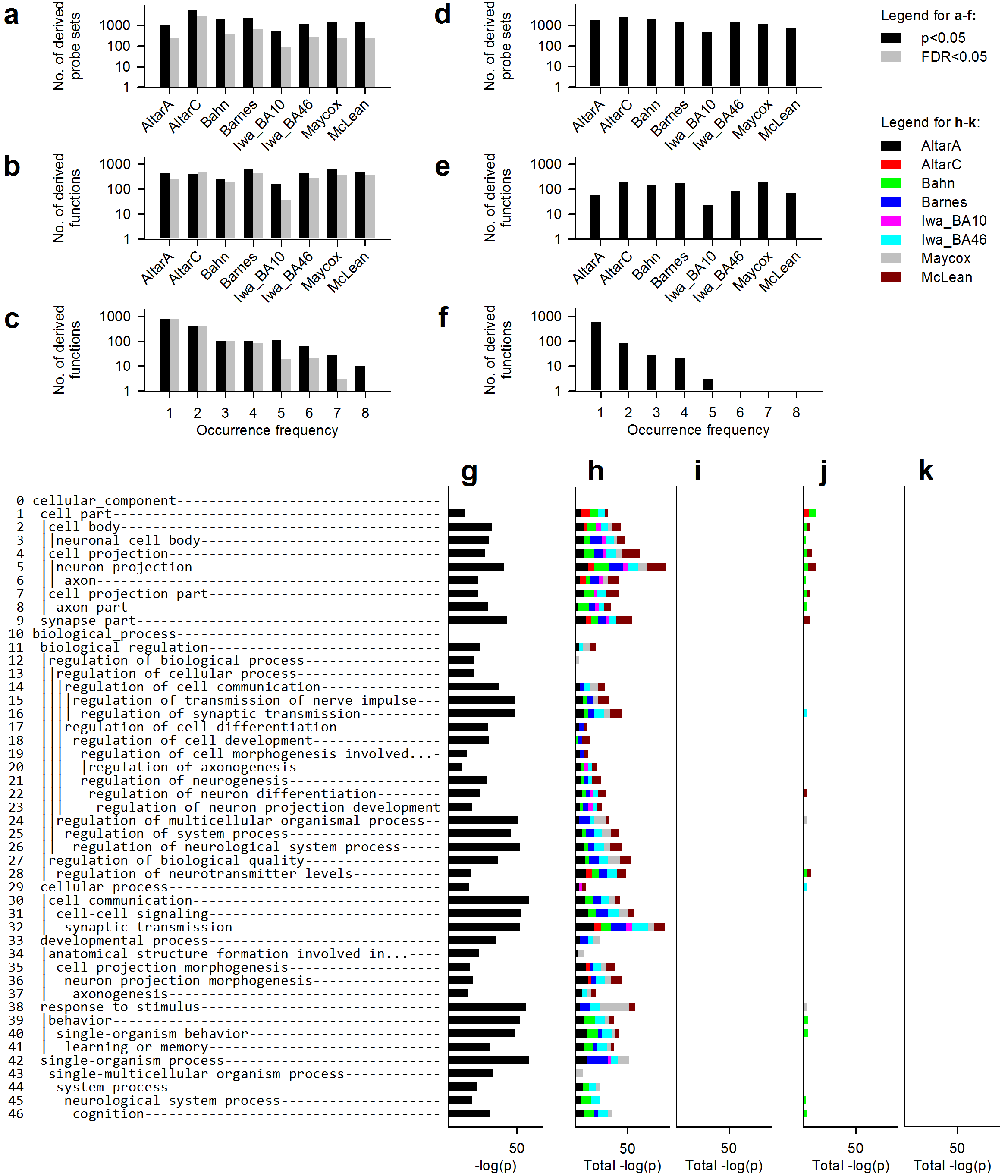

Supplement: S4 Fig — (a) Numbers of HTA-derived probe sets. (b) Numbers of HTA-derived functions. (c) Occurrence frequency distributions of HTA-derived functions. (d) Numbers of SAM-derived probe sets. (e) Numbers of SAM-derived functions. (f) Occurrence frequency distributions of SAM-derived functions. (g) Bias of the SZGene list towards the neural functions. (h) Biases of HTA-derived probe sets towards the neural functions. (i) The biases in (h) expected by chance. (j) Biases of SAM-derived probe sets towards the neural functions. (k) The biases in (j) expected by chance. (TIF) [file pone.0121154.s004.TIF]

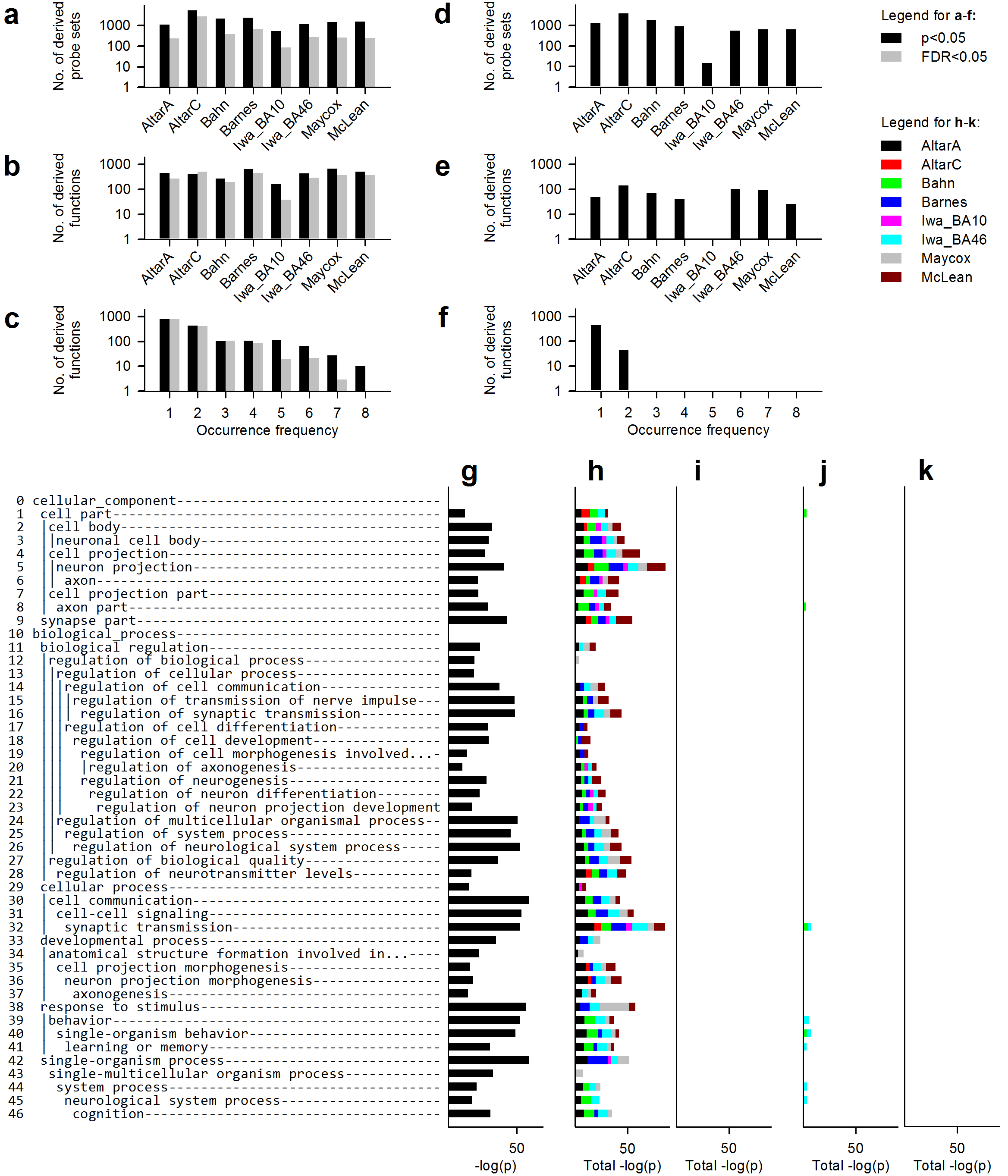

Supplement: S5 Fig — (a) Numbers of HTA-derived probe sets. (b) Numbers of HTA-derived functions. (c) Occurrence frequency distributions of HTA-derived functions. (d) Numbers of limma-derived probe sets. (e) Numbers of limma-derived functions. (f) Occurrence frequency distributions of limma-derived functions. (g) Bias of the SZGene list towards the neural functions. (h) Biases of HTA-derived probe sets towards the neural functions. (i) The biases in (h) expected by chance. (j) Biases of limma-derived probe sets towards the neural functions. (k) The biases in (j) expected by chance. (TIF) [file pone.0121154.s005.TIF]

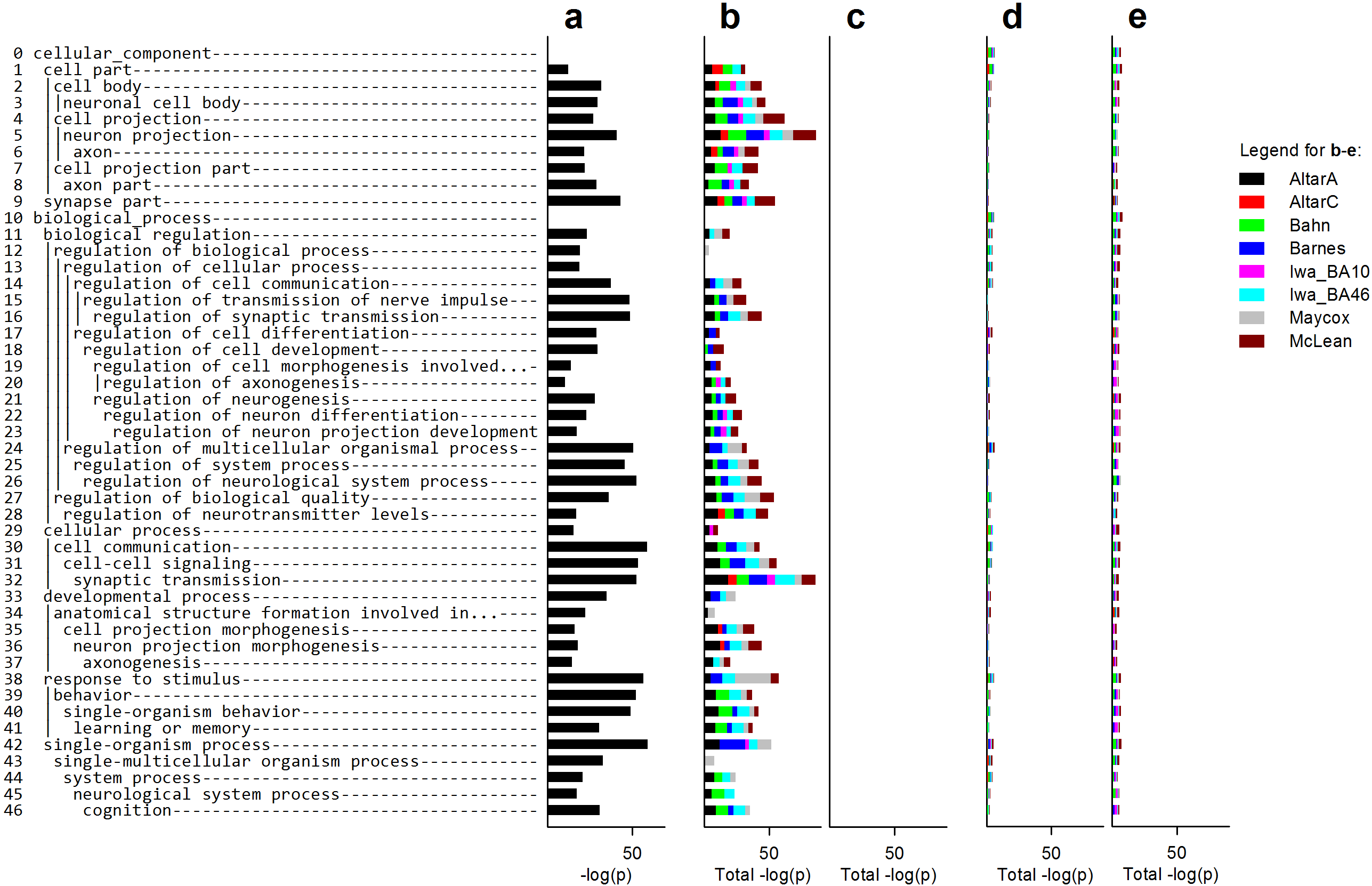

Supplement: S6 Fig — (a) Bias of the SZGene list towards the neural functions. (b) Biases of HTA-derived probe sets towards the neural functions. (c) The biases in (b) expected by chance. (d) GSEA-derived biases towards the neural functions. (e) The biases in (d) expected by chance. (TIF) [file pone.0121154.s006.TIF]

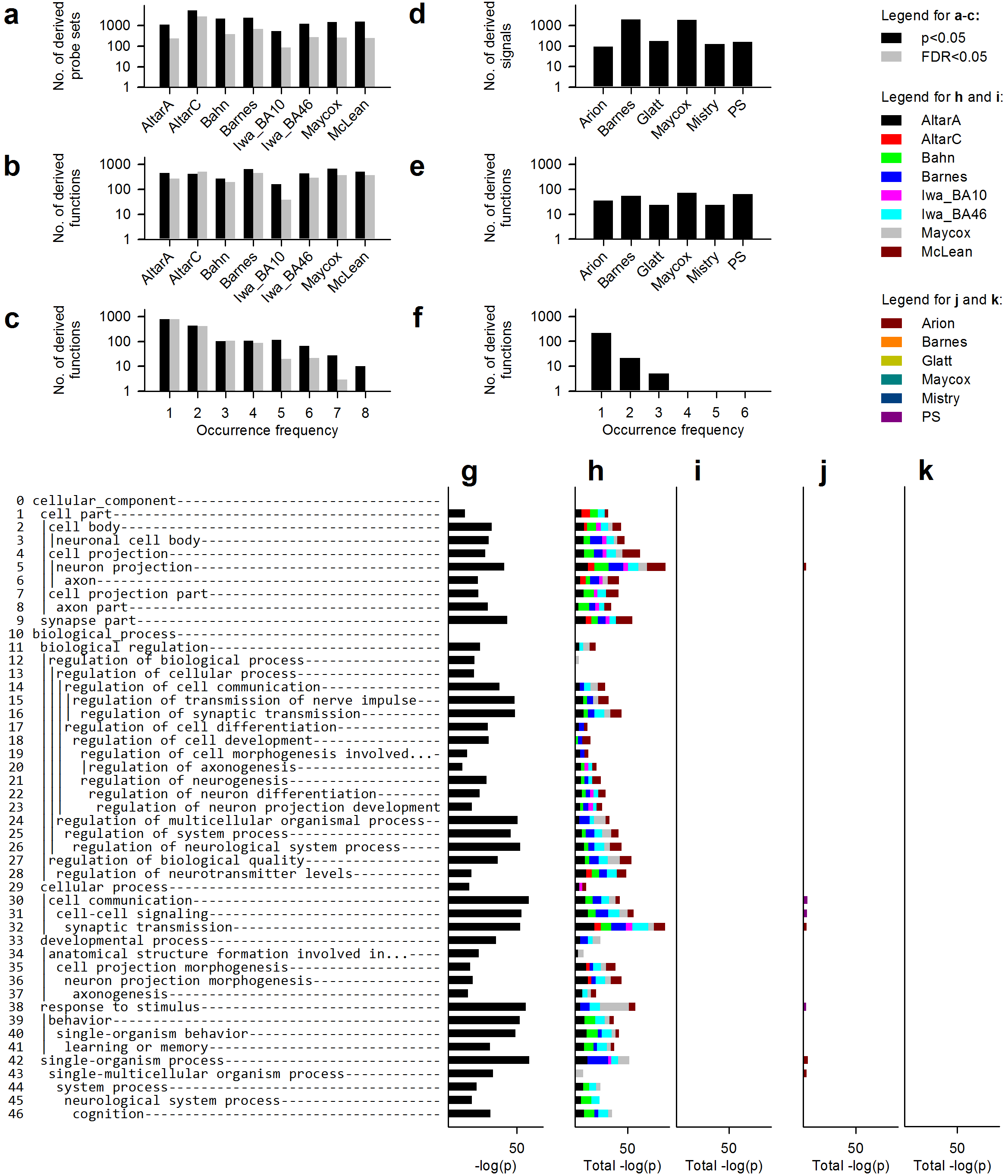

Supplement: S7 Fig — (a) Numbers of HTA-derived probe sets. (b) Numbers of HTA-derived functions. (c) Occurrence frequency distributions of HTA-derived functions. (d) Numbers of the literature-reported signals. (e) Numbers of functions derived from the literature-reported signals. (f) Occurrence frequency distributions of the functions in (e). (g) Bias of the SZGene list towards the neural functions. (h) Biases of HTA-derived signals towards the neural functions. (i) The biases in (h) expected by chance. (j) Biases of the literature-reported signals towards the neural functions. (k) The biases in (j) derived by chance. (TIF) [file pone.0121154.s007.TIF]

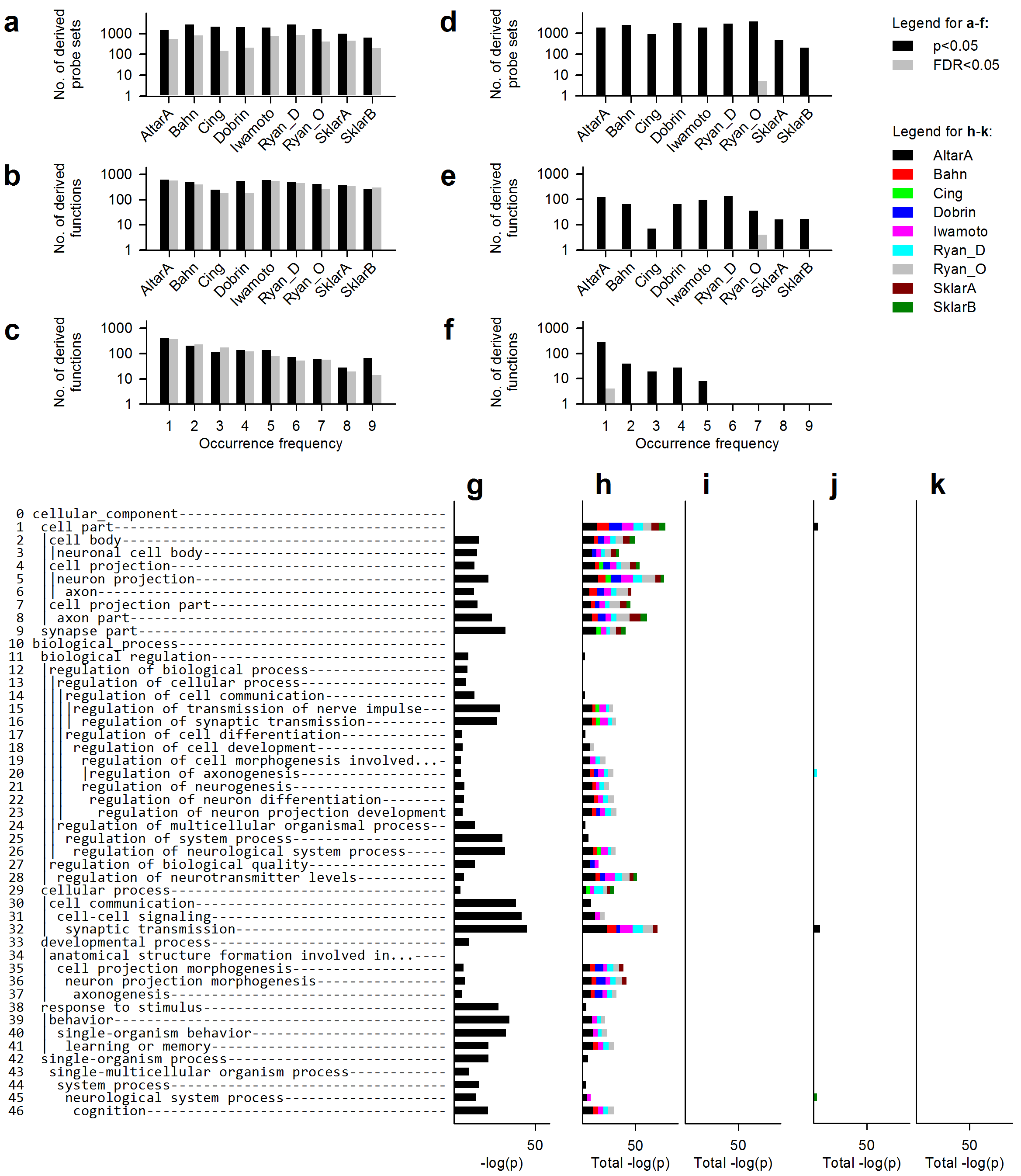

Supplement: S8 Fig — (a) Numbers of HTA-derived probe sets. (b) Numbers of HTA-derived functions. (c) Occurrence frequency distributions of HTA-derived functions. (d) Numbers of t-test-derived probe sets. (e) Numbers of t-test-derived functions. (f) Occurrence frequency distributions of t-test-derived functions. (g) Bias of the BDGene list towards the neural functions. (h) Biases of HTA-derived probe sets towards the neural functions. (i) The biases in (h) expected by chance. (j) Biases of t-test-derived probe sets towards the neural functions. (k) The biases in (j) expected by chance. (TIF) [file pone.0121154.s008.TIF]

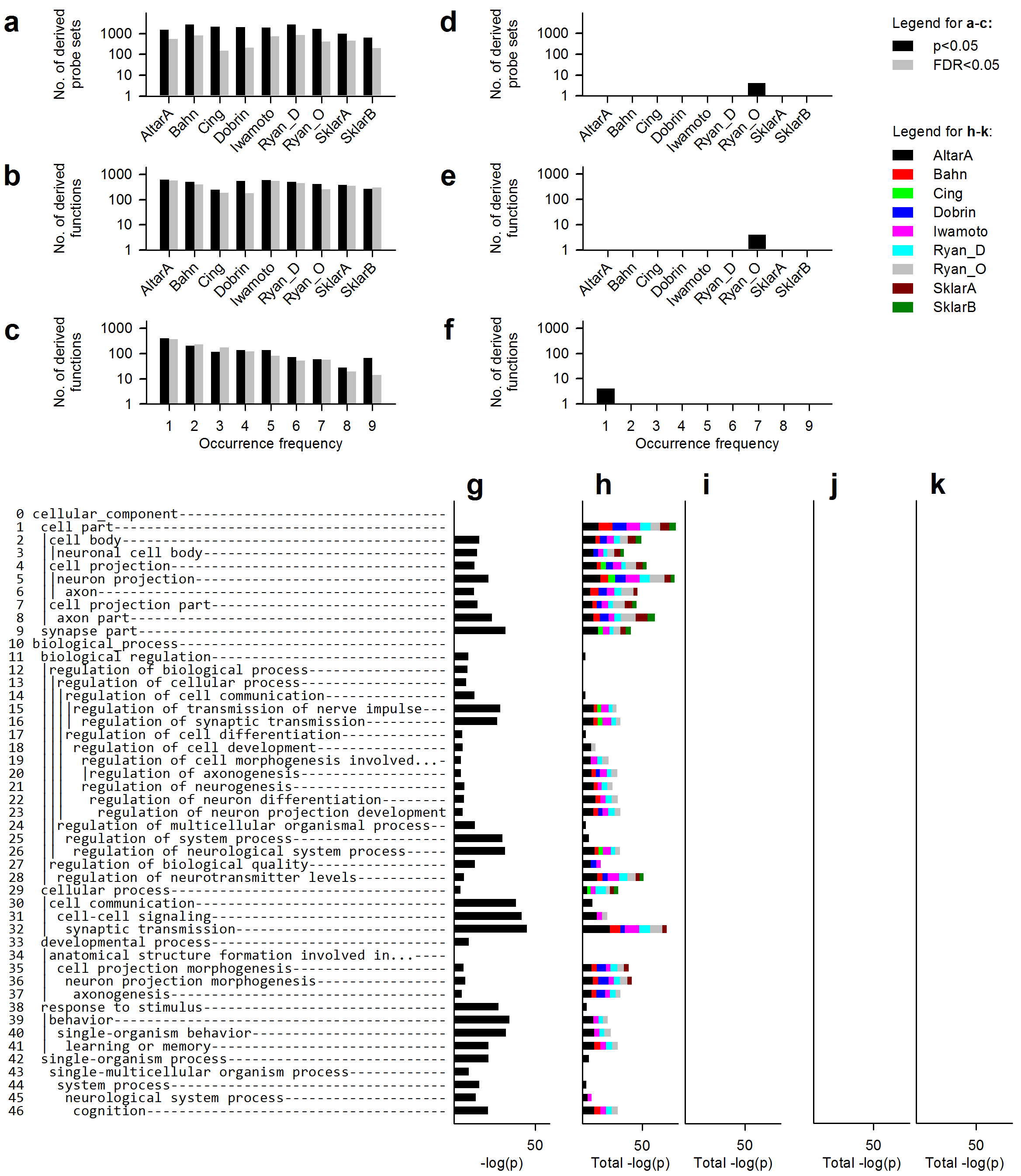

Supplement: S9 Fig — (a) Numbers of HTA-derived probe sets. (b) Numbers of HTA-derived functions. (c) Occurrence frequency distributions of HTA-derived functions. (d) Numbers of HM-derived probe sets. (e) Numbers of HM-derived functions. (f) Occurrence frequency distributions of HM-derived functions. (g) Bias of the BDGene list towards the neural functions. (h) Biases of HTA-derived probe sets towards the neural functions. (i) The biases in (h) expected by chance. (j) Biases of HM-derived probe sets towards the neural functions. (k) The biases in (j) expected by chance. (TIF) [file pone.0121154.s009.TIF]

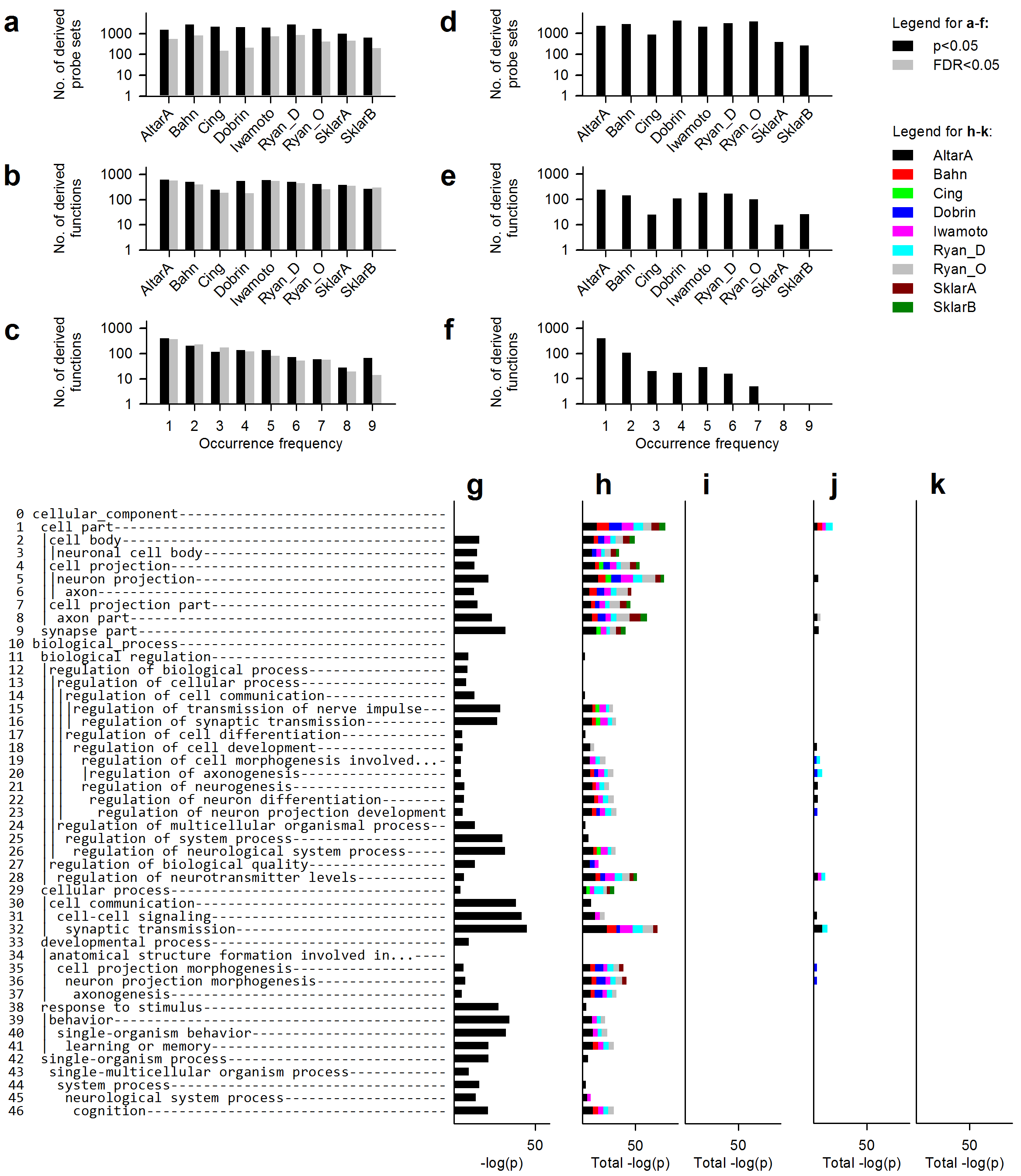

Supplement: S10 Fig — (a) Numbers of HTA-derived probe sets. (b) Numbers of HTA-derived functions. (c) Occurrence frequency distributions of HTA-derived functions. (d) Numbers of SAM-derived probe sets. (e) Numbers of SAM-derived functions. (f) Occurrence frequency distributions of SAM-derived functions. (g) Bias of the BDGene list towards the neural functions. (h) Biases of HTA-derived probe sets towards the neural functions. (i) The biases in (h) expected by chance. (j) Biases of SAM-derived probe sets towards the neural functions. (k) The biases in (j) expected by chance. (TIF) [file pone.0121154.s010.TIF]

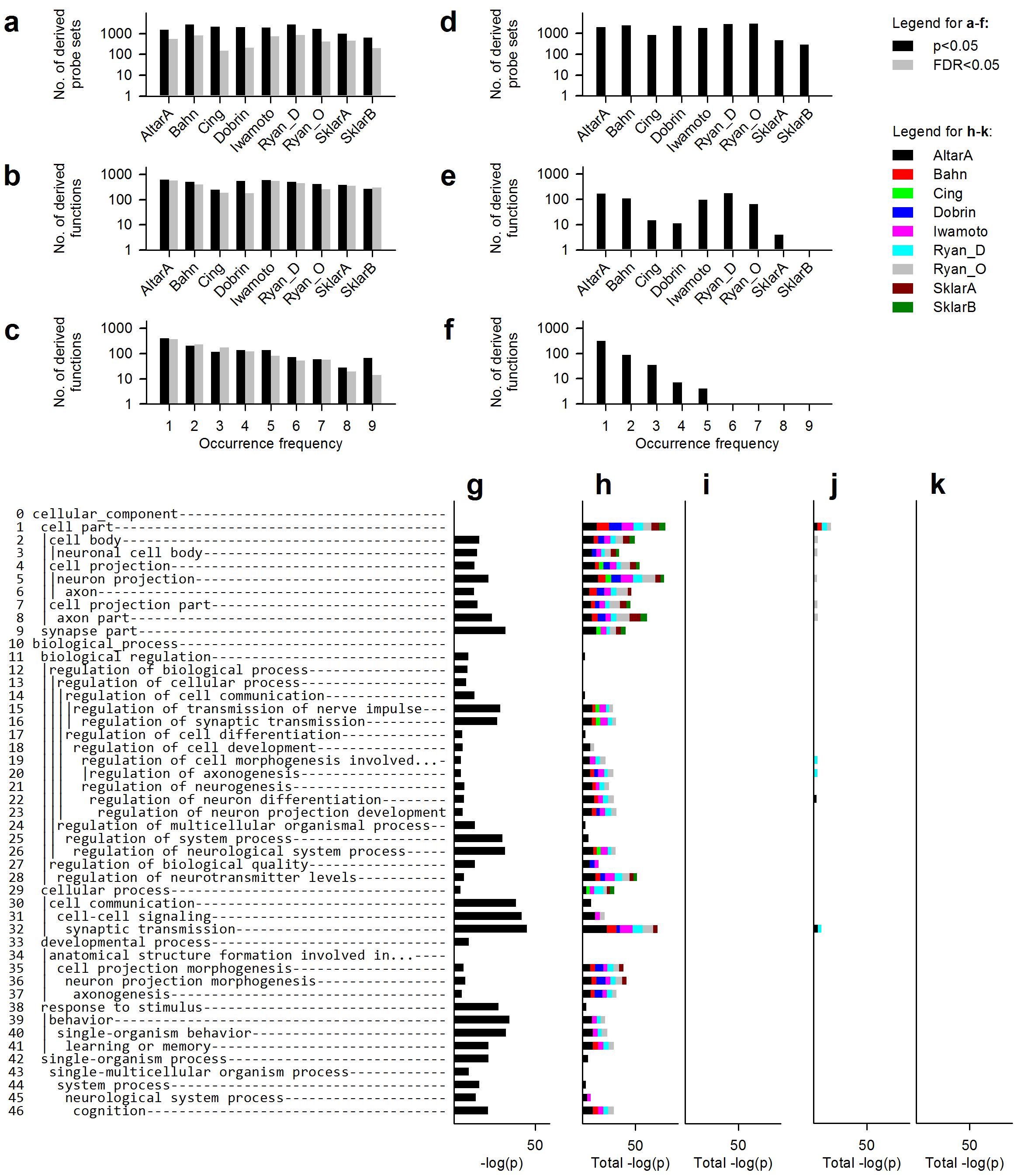

Supplement: S11 Fig — (a) Numbers of HTA-derived probe sets. (b) Numbers of HTA-derived functions. (c) Occurrence frequency distributions of HTA-derived functions. (d) Numbers of limma-derived probe sets. (e) Numbers of limma-derived functions. (f) Occurrence frequency distributions of limma-derived functions. (g) Bias of the BDGene list towards the neural functions. (h) Biases of HTA-derived probe sets towards the neural functions. (i) The biases in (h) expected by chance. (j) Biases of limma-derived probe sets towards the neural functions. (k) The biases in (j) expected by chance. (TIF) [file pone.0121154.s011.TIF]

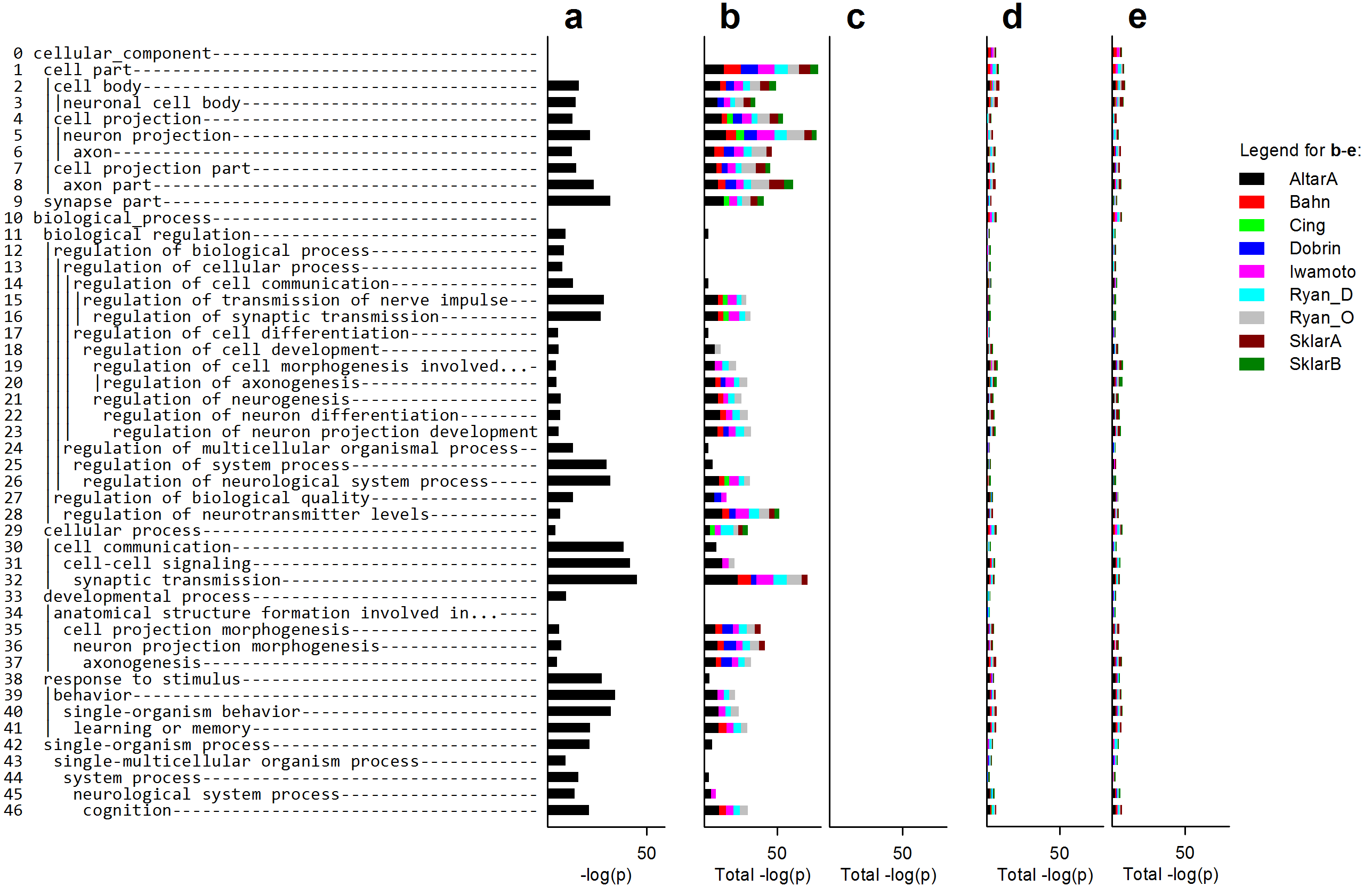

Supplement: S12 Fig — (a) Bias of the BDGene list towards the neural functions. (b) Biases of HTA-derived probe sets towards the neural functions. (c) The biases in (b) expected by chance. (d) GSEA-derived biases towards the neural functions. (e) The biases in (d) expected by chance. (TIF) [file pone.0121154.s012.TIF]

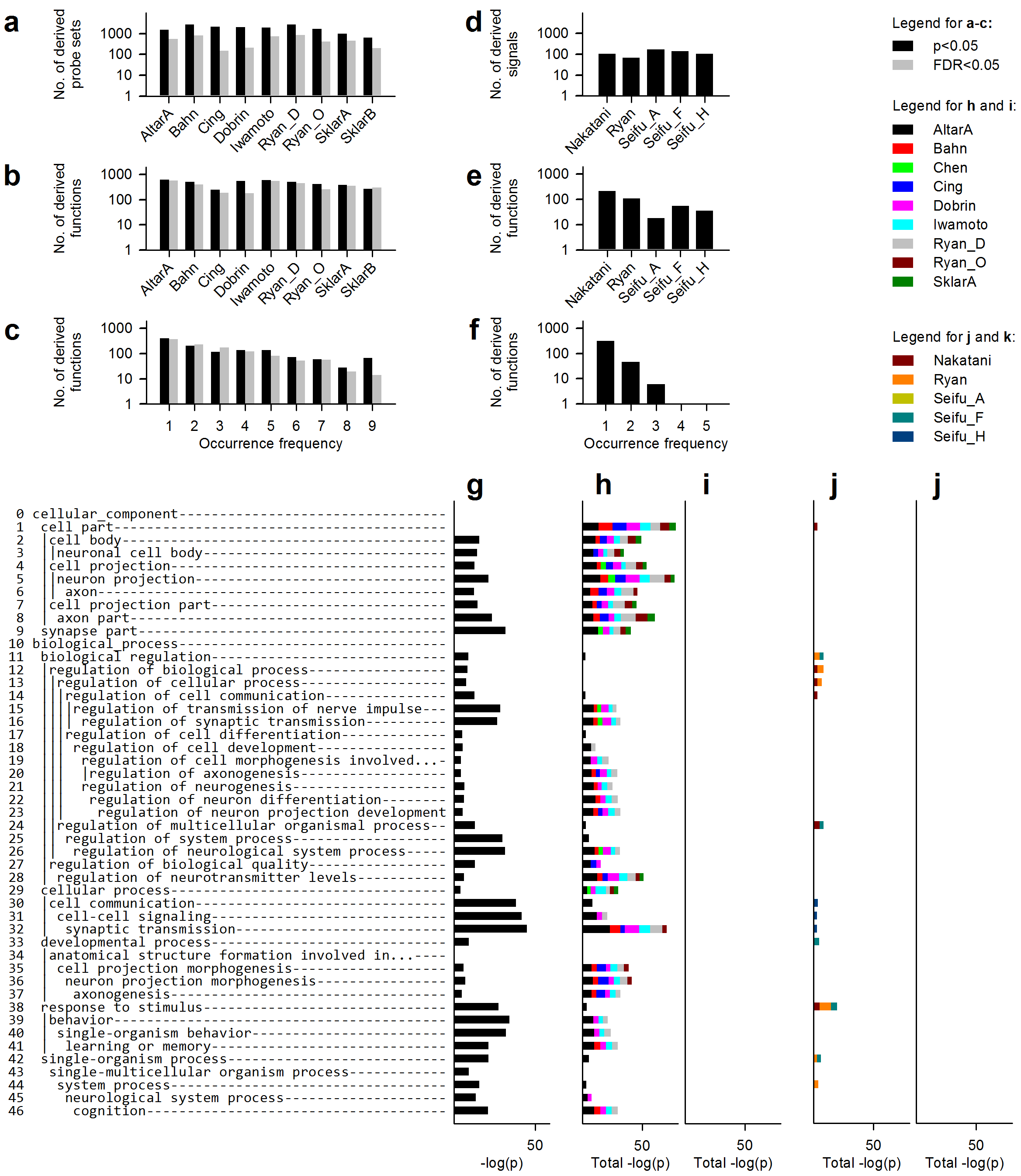

Supplement: S13 Fig — (a) Numbers of HTA-derived probe sets. (b) Numbers of HTA-derived functions. (c) Occurrence frequency distributions of HTA-derived functions. (d) Numbers of the literature-reported signals. (e) Numbers of functions derived from the literature-reported signals. (f) Occurrence frequency distributions of the functions in (e). (g) Bias of the BDGene list towards the neural functions. (h) Biases of HTA-derived signals towards the neural functions. (i) The biases in (h) expected by chance. (j) Biases of the literature-reported signals towards the neural functions. (k) The biases in (j) derived by chance. (TIF) [file pone.0121154.s013.TIF]

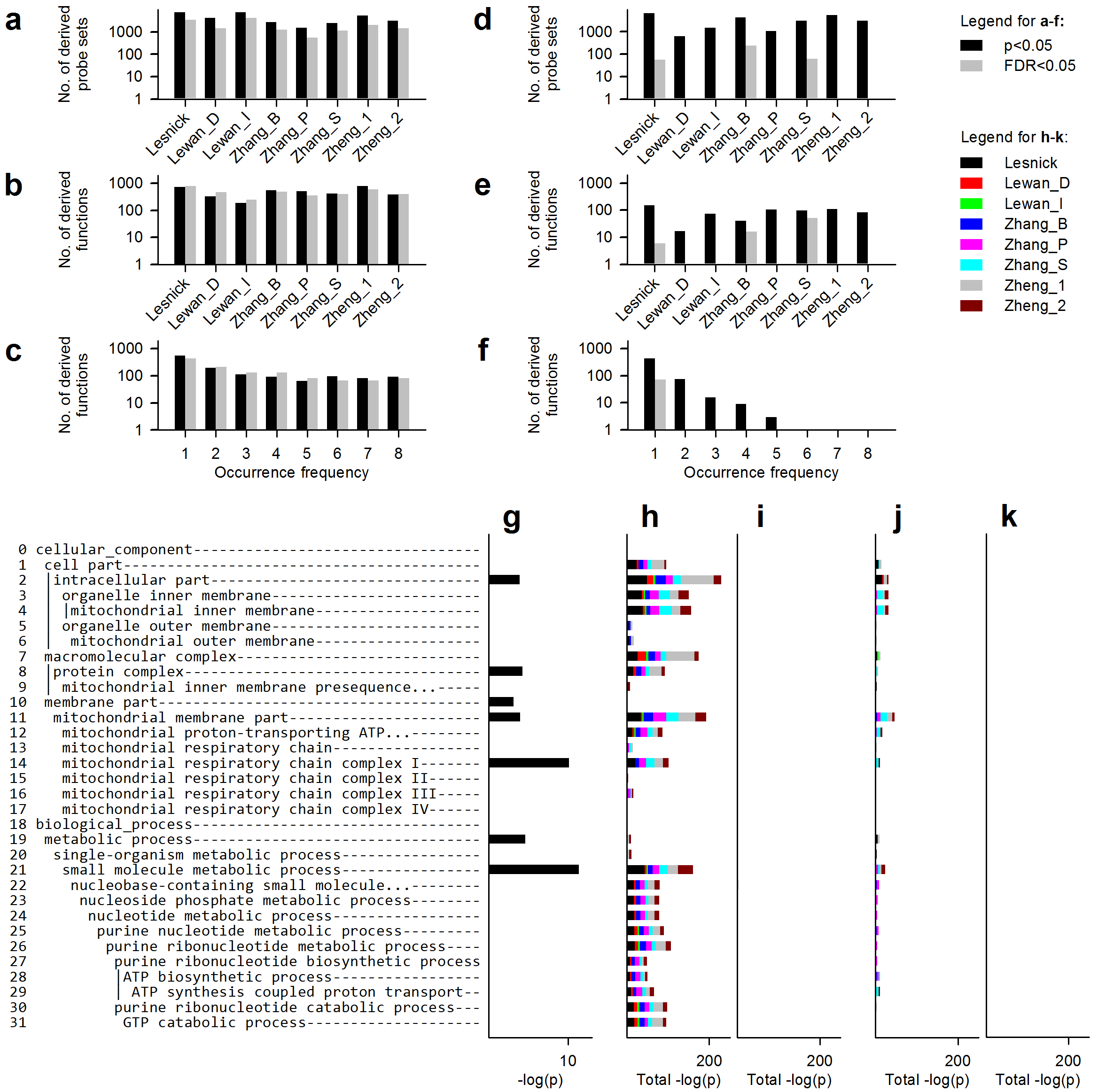

Supplement: S14 Fig — (a) Numbers of HTA-derived probe sets. (b) Numbers of HTA-derived functions. (c) Occurrence frequency distributions of HTA-derived functions. (d) Numbers of t-test-derived probe sets. (e) Numbers of t-test-derived functions. (f) Occurrence frequency distributions of t-test-derived functions. (g) Bias of the PDGene list towards the mitochondrial functions. (h) Biases of HTA-derived probe sets towards the mitochondrial functions. (i) The biases in (h) expected by chance. (j) Biases of t-test-derived probe sets towards the mitochondrial functions. (k) The biases in (j) expected by chance. (TIF) [file pone.0121154.s014.TIF]

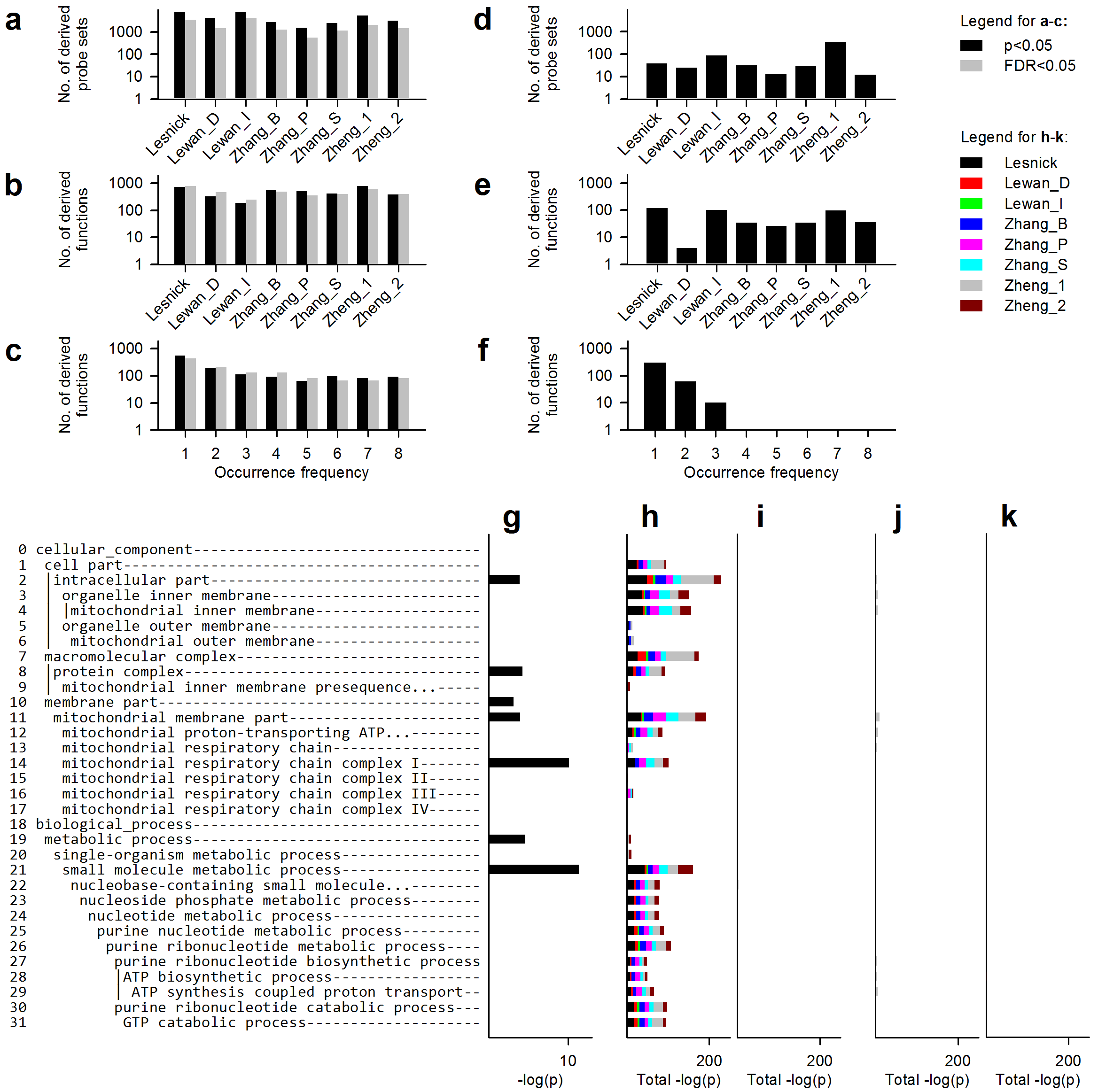

Supplement: S15 Fig — (a) Numbers of HTA-derived probe sets. (b) Numbers of HTA-derived functions. (c) Occurrence frequency distributions of HTA-derived functions. (d) Numbers of HM-derived probe sets. (e) Numbers of HM-derived functions. (f) Occurrence frequency distributions of HM-derived functions. (g) Bias of the PDGene list towards the mitochondrial functions. (h) Biases of HTA-derived probe sets towards the mitochondrial functions. (i) The biases in (h) expected by chance. (j) Biases of HM-derived probe sets towards the mitochondrial functions. (k) The biases in (j) expected by chance. (TIF) [file pone.0121154.s015.TIF]

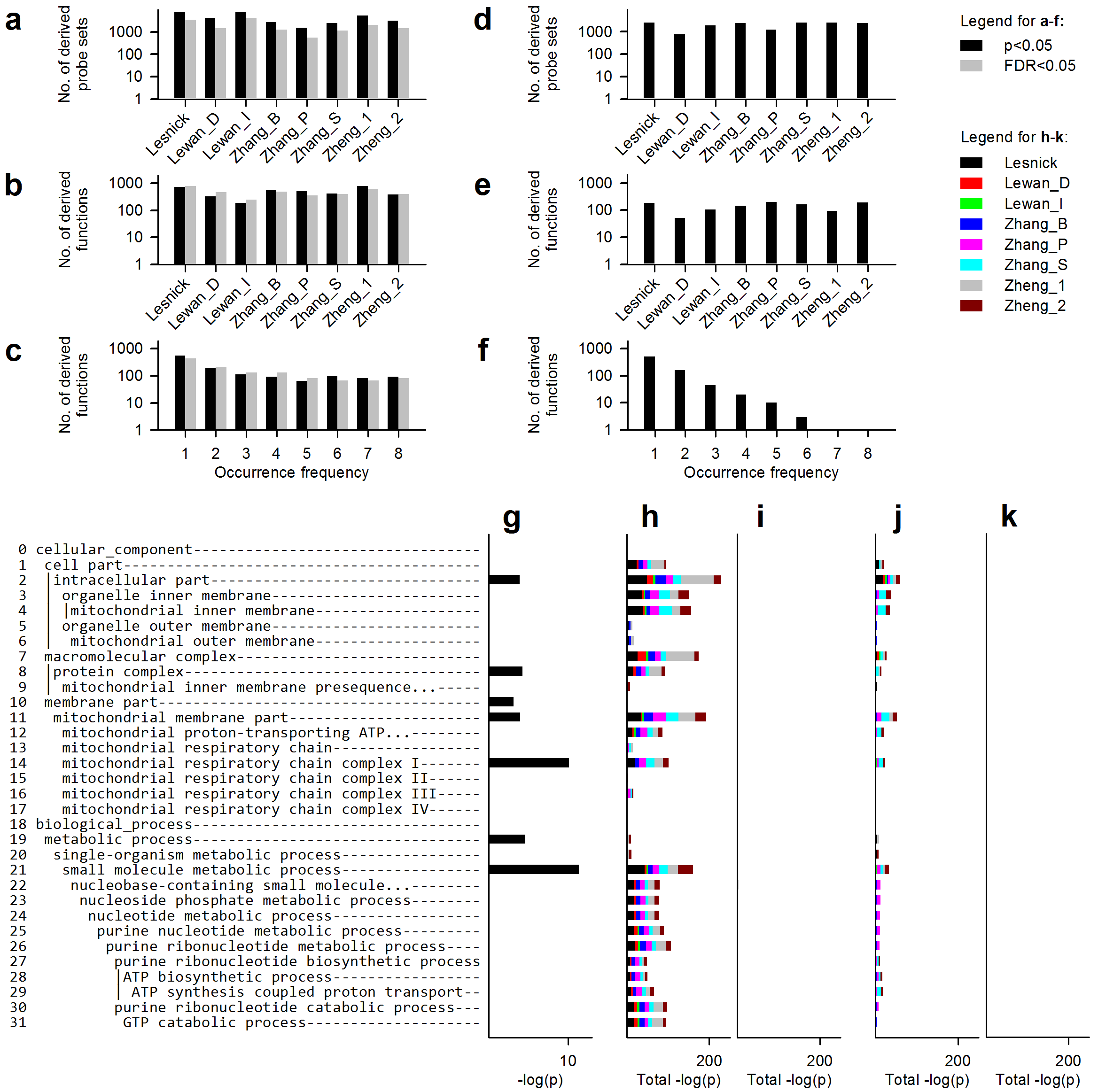

Supplement: S16 Fig — (a) Numbers of HTA-derived probe sets. (b) Numbers of HTA-derived functions. (c) Occurrence frequency distributions of HTA-derived functions. (d) Numbers of SAM-derived probe sets. (e) Numbers of SAM-derived functions. (f) Occurrence frequency distributions of SAM-derived functions. (g) Bias of the PDGene list towards the mitochondrial functions. (h) Biases of HTA-derived probe sets towards the mitochondrial functions. (i) The biases in (h) expected by chance. (j) Biases of SAM-derived probe sets towards the mitochondrial functions. (k) The biases in (j) expected by chance. (TIF) [file pone.0121154.s016.TIF]

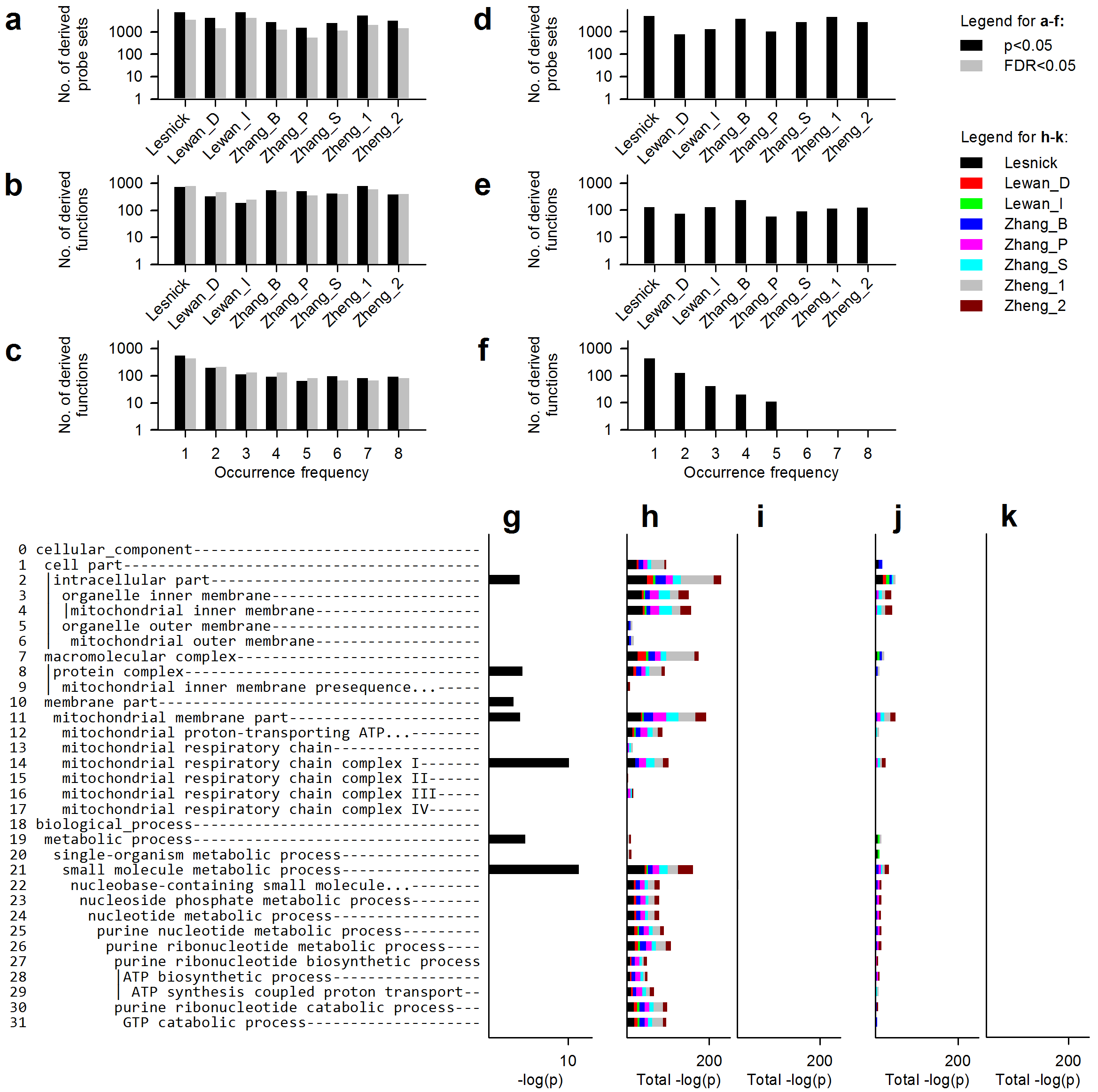

Supplement: S17 Fig — (a) Numbers of HTA-derived probe sets. (b) Numbers of HTA-derived functions. (c) Occurrence frequency distributions of HTA-derived functions. (d) Numbers of limma-derived probe sets. (e) Numbers of limma-derived functions. (f) Occurrence frequency distributions of limma-derived functions. (g) Bias of the PDGene list towards the mitochondrial functions. (h) Biases of HTA-derived probe sets towards the mitochondrial functions. (i) The biases in (h) expected by chance. (j) Biases of limma-derived probe sets towards the mitochondrial functions. (k) The biases in (j) expected by chance. (TIF) [file pone.0121154.s017.TIF]

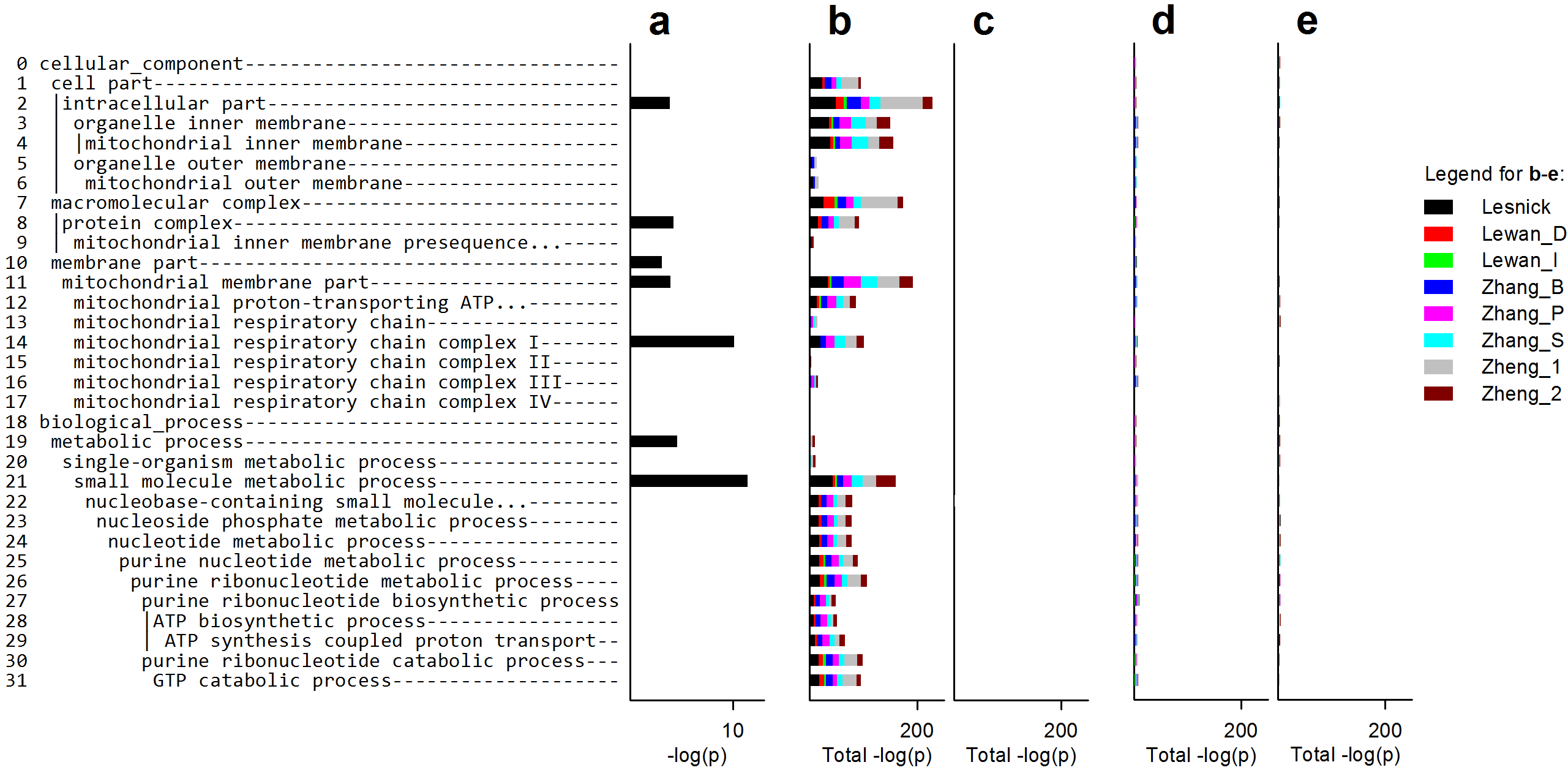

Supplement: S18 Fig — (a) Bias of the PDGene list towards the mitochondrial functions. (b) Biases of HTA-derived probe sets towards the mitochondrial functions. (c) The biases in (b) expected by chance. (d) GSEA-derived biases towards the mitochondrial functions. (e) The biases in (d) expected by chance. (TIF) [file pone.0121154.s018.TIF]

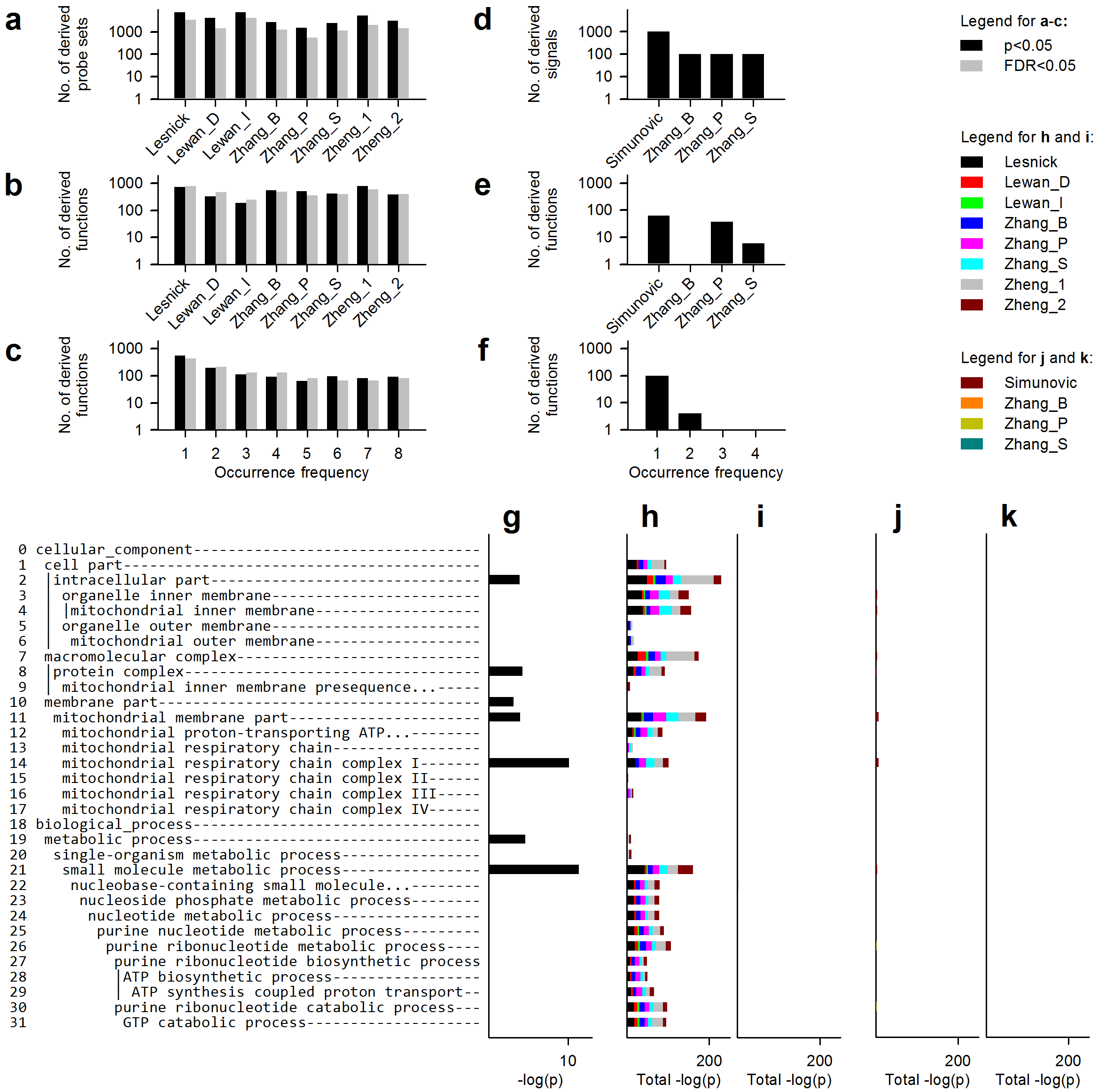

Supplement: S19 Fig — (a) Numbers of HTA-derived probe sets. (b) Numbers of HTA-derived functions. (c) Occurrence frequency distributions of HTA-derived functions. (d) Numbers of the literature-reported signals. (e) Numbers of functions derived from the literature-reported signals. (f) Occurrence frequency distributions of the functions in (e). (g) Bias of the PDGene list towards the mitochondrial functions. (h) Biases of HTA-derived signals towards the mitochondrial functions. (i) The biases in (h) expected by chance. (j) Biases of the literature-reported signals towards the mitochondrial functions. (k) The biases in (j) expected by chance. (TIF) [file pone.0121154.s019.TIF]

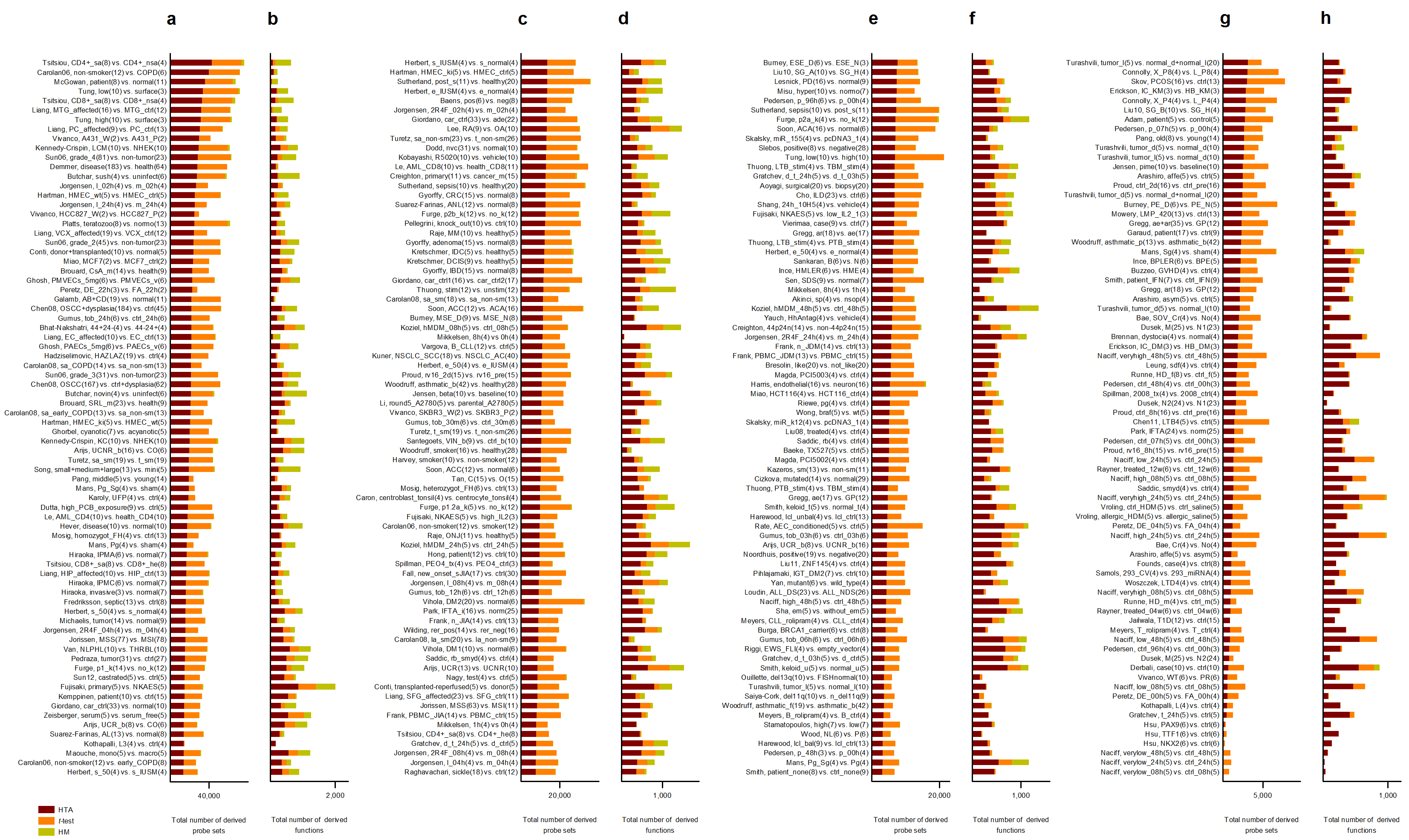

Supplement: S20 Fig — Number in parentheses is number of subjects. (a,c,e,g) Numbers of probe sets. (b,d,f,h) Numbers of functions. (TIF) [file pone.0121154.s020.TIF]

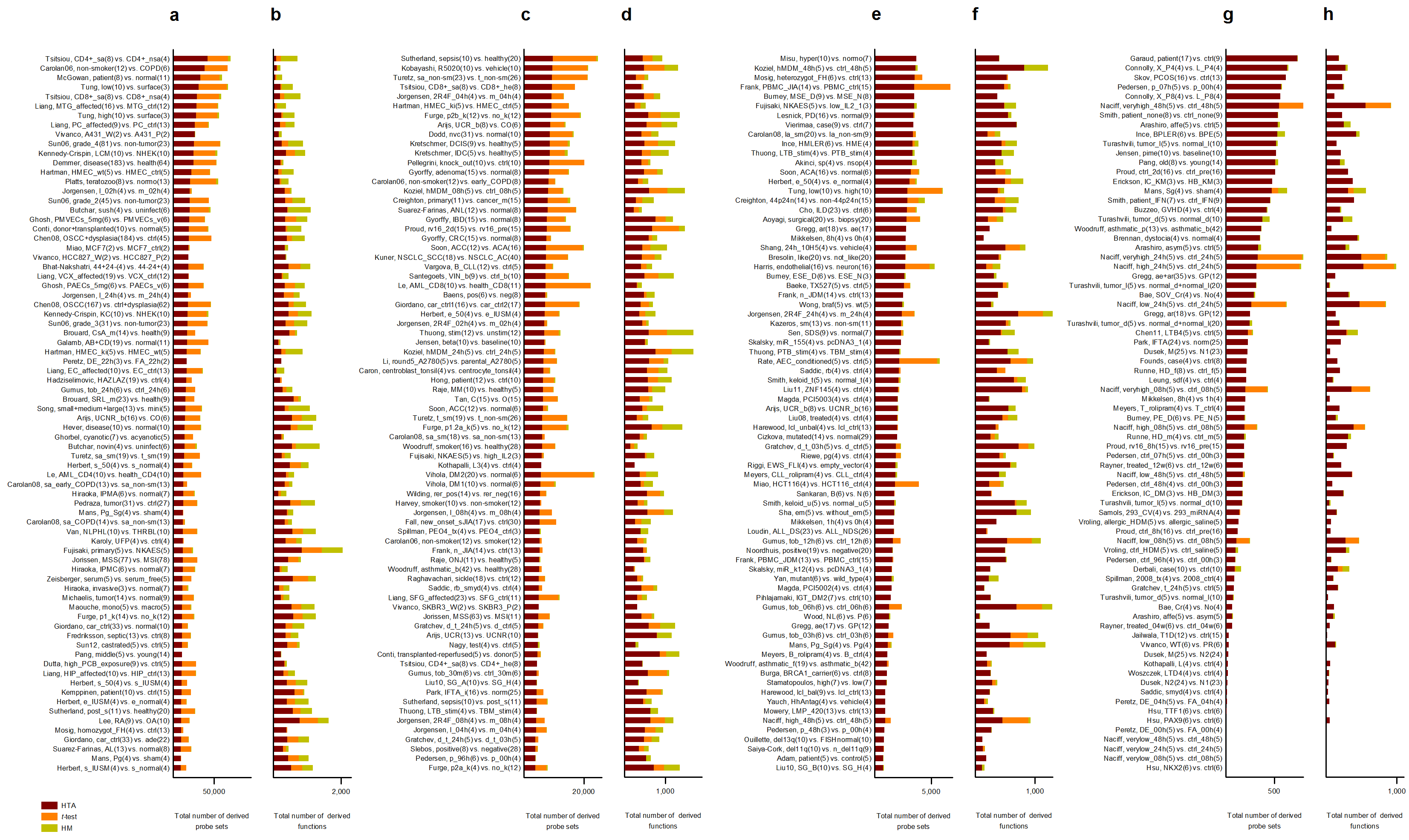

Supplement: S21 Fig — Number in parentheses is number of subjects. (a,c,e,g) Numbers of probe sets. (b,d,f,h) Numbers of functions. (TIF) [file pone.0121154.s021.TIF]

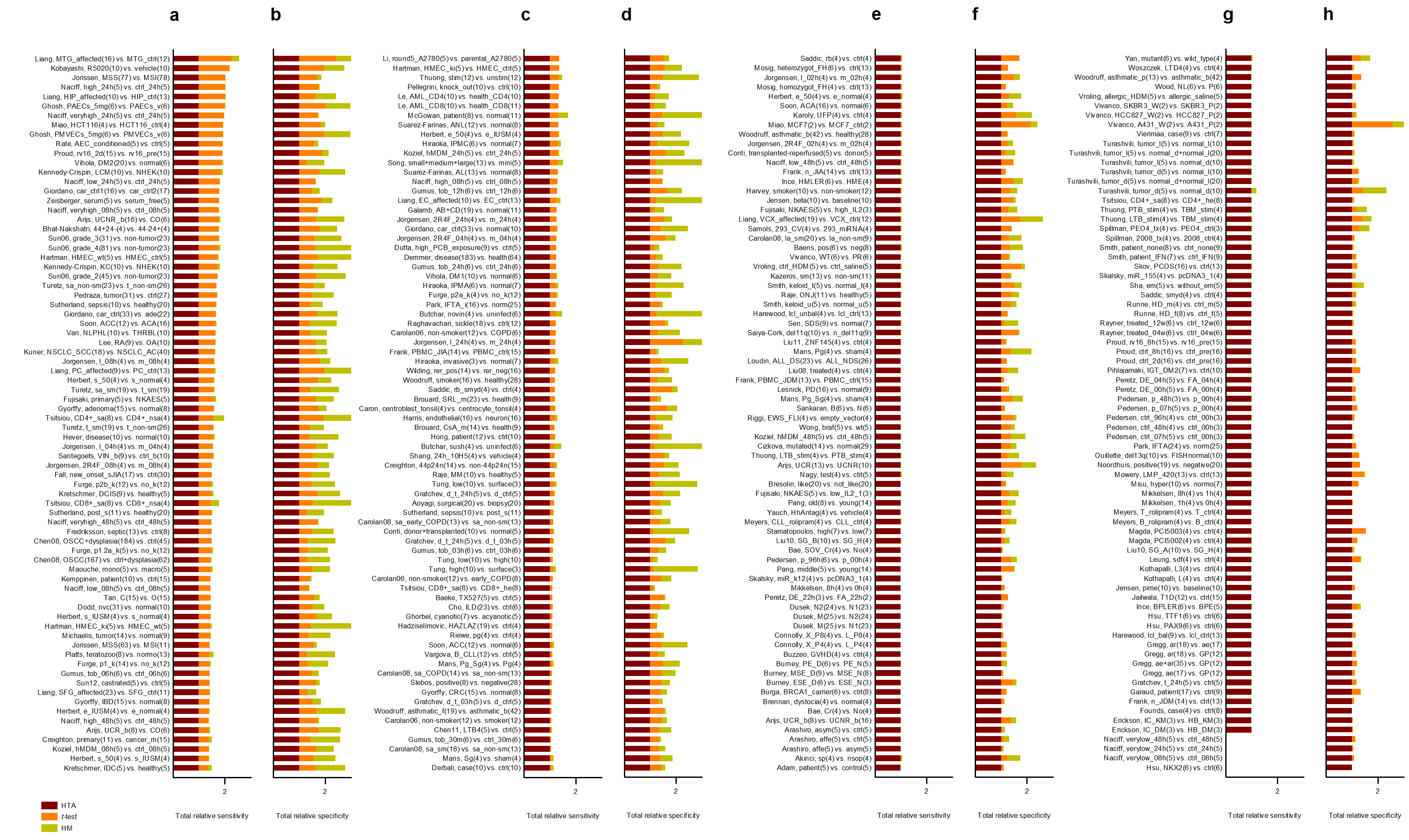

Supplement: S22 Fig — The 304 contrasts were used. Number in parentheses is number of subjects. (a,c,e,g) Relative sensitivity of the methods. (b,d,f,h) Relative specificity of the methods. (TIF) [file pone.0121154.s022.TIF]
