# Supplementary material for: Method Designed to Respect Molecular Heterogeneity Can Profoundly Correct Present Data Interpretations for Genome-Wide Expression Analysis
Source: PLoS One. 2015 Mar 20;10(3):e0121154. doi: 10.1371/journal.pone.0121154 (PMC4368820; doi:10.1371/journal.pone.0121154)
Supplement: S1 Table — (PDF) [file pone.0121154.s023.pdf]

Accession no.	Reference	Contrast
GSE14975	Adam et al. 2010 [1]	patient(5) vs. control(5)
GSE12631	Akinci et al. 2009 [2]	sp(4) vs. nsop(4)
GSE32701	Aoyagi et al. 2011 [3]	surgical(20) vs. biopsy(20)
GSE15090	Arashiro et al. 2009 [4]	affe(5) vs. asym(5)
		affe(5) vs. ctrl(5)
		asym(5) vs. ctrl(5)
GSE14580	Arijs et al. 2009 [5]	UCNR_b(16) vs. CO(6)
		UCR_b(8) vs. CO(6)
		UCR_b(8) vs. UCNR_b(16)
GSE12251	Arijs et al. 2009 [5]	UCR(13) vs. UCNR(10)
GSE12205	Bae et al. 2009 [6]	Cr(4) vs. No(4)
		SOV_Cr(4) vs. No(4)
GSE23984	Baeke et al. 2011 [7]	TX527(5) vs. ctrl(5)
GSE25550	Baens et al. 2011 [8]	pos(6) vs. neg(8)
GSE15192	Bhat-Nakshatri et al. 2010 [9]	44+24-(4) vs. 44-24+(4)
GSE32178	Brennan et al. 2011 [10]	dystocia(4) vs. normal(4)
GSE14858	Bresolin et al. 2009 [11]	like(20) vs. not_like(20)
GSE22224	Brouard et al. 2010 [12]	CsA_m(14) vs. health(9)
		SRL_m(23) vs. health(9)
GSE13671	Burga et al. 2009 [13]	BRCA1_carrier(6) vs. ctrl(8)
GSE6364	Burney et al. 2007 [14]	ESE_D(6) vs. ESE_N(3)
		MSE_D(9) vs. MSE_N(8)
		PE_D(6) vs. PE_N(5)
GSE12108	Butchar et al. 2008 [15]	novin(4) vs. uninfect(6)
		sush(4) vs. uninfect(6)
GSE7510	Buzzeo et al. 2008 [16]	GVHD(4) vs. ctrl(4)
GSE5058	Carolan et al. 2006 [17]	non-smoker(12) vs. COPD(6)
		non-smoker(12) vs. early_COPD(8)
		non-smoker(12) vs. smoker(12)
GSE10006	Carolan et al. 2008 [18]	la_sm(20) vs. la_non-sm(9)
		sa_COPD(14) vs. sa_non-sm(13)
		sa_early_COPD(13) vs. sa_non-sm(13)
		sa_sm(18) vs. sa_non-sm(13)
GSE15271	Caron et al. 2009 [19]	centroblast_tonsil(4) vs. centrocyte_tonsil(4)
GSE30784	Chen et al. 2008 [20]	OSCC(167) vs. ctrl+dysplasia(62)
		OSCC+dysplasia(184) vs. ctrl(45)
GSE24869	Chen et al. 2011 [21]	LTB4(5) vs. ctrl(5)
GSE21369	Cho et al. 2011 [22]	ILD(23) vs. ctrl(6)
GSE22035	Cizkova et al. 2010 [23]	mutated(14) vs. normal(29)
GSE11618	Connolly et al. 2009 [24]	X_P4(4) vs. L_P4(4)
		X_P8(4) vs. L_P8(4)
GSE14951	Conti et al. 2007 [25]	donor+transplanted(10) vs. normal(5)
		transplanted-reperfused(5) vs. donor(5)
GSE7513	Creighton et al. 2009 [26]	44p24n(14) vs. non-44p24n(15)
GSE7515		primary(11) vs. cancer_m(15)
GSE10334	Demmer et al. 2008 [27]	disease(183) vs. health(64)
GSE12644	Derbali et al. 2010 [28]	case(10) vs. ctrl(10)
GSE12452	Dodd et al. 2006 [29]	nvc(31) vs. normal(10)
GSE10041	Dusek et al. 2008 [30]	M(25) vs. N1(23)
		M(25) vs. N2(24)
		N2(24) vs. N1(23)
GSE22868	Dutta et al. 2012 [31]	high_PCB_exposure(9) vs. ctrl(5)
GSE11839	Erickson et al. 2008 [32]	IC_DM(3) vs. HB_DM(3)
		IC_KM(3) vs. HB_KM(3)
GSE7753	Fall et al. 2007 [33]	new_onset_sJIA(17) vs. ctrl(30)
GSE12767	Founds et al. 2009 [34]	case(4) vs. ctrl(8)

Accession no.	Reference	Contrast
GSE11083	Frank et al. 2009 [35]	PBMC_JDM(13) vs. PBMC_ctrl(15)
		PBMC_JIA(14) vs. PBMC_ctrl(15)
		n_JDM(14) vs. ctrl(13)
		n_JIA(14) vs. ctrl(13)
GSE13205	Fredriksson et al. 2008 [36]	septic(13) vs. ctrl(8)
GSE12198	Fujisaki et al. 2009 [37]	NKAES(5) vs. high_IL2(3)
		NKAES(5) vs. low_IL2_1(3)
		primary(5) vs. NKAES(5)
GSE7023	Furge et al. 2007 [38]	p1.2a_k(5) vs. no_k(12)
		p1_k(14) vs. no_k(12)
		p2a_k(4) vs. no_k(12)
		p2b_k(12) vs. no_k(12)
GSE10715	Galamb et al. 2008 [39]	AB+CD(19) vs. normal(11)
GSE30153	Garaud et al. 2011 [40]	patient(17) vs. ctrl(9)
GSE14970	Ghorbel et al. 2010 [41]	cyanotic(7) vs. acyanotic(5)
GSE25014	Ghosh et al. 2011 [42]	PAECs_5mg(6) vs. PAECs_v(6)
		PMVECs_5mg(6) vs. PMVECs_v(6)
GSE10927	Giordano et al. 2009 [43]	car_ctrl(33) vs. ade(22)
		car_ctrl(33) vs. normal(10)
		car_ctrl1(16) vs. car_ctrl2(17)
GSE7568	Gratchev et al. 2008 [44]	d_t_03h(5) vs. d_ctrl(5)
		d_t_24h(5) vs. d_ctrl(5)
		d_t_24h(5) vs. d_t_03h(5)
		t_24h(5) vs. ctrl(5)
GSE6575	Gregg et al. 2008 [45]	ae(17) vs. GP(12)
		ae+ar(35) vs. GP(12)
		ar(18) vs. GP(12)
		ar(18) vs. ae(17)
GSE10063	Gumus et al. 2008 [46]	tob_03h(6) vs. ctrl_03h(6)
		tob_06h(6) vs. ctrl_06h(6)
		tob_12h(6) vs. ctrl_12h(6)
		tob_24h(6) vs. ctrl_24h(6)
		tob_30m(6) vs. ctrl_30m(6)
GSE4183	Gyorffy et al. 2010 [47]	CRC(15) vs. normal(8)
		IBD(15) vs. normal(8)
		adenoma(15) vs. normal(8)
GSE25518	Hadziselimovic et al. 2011 [48]	HAZLAZ(19) vs. ctrl(4)
GSE13122	Harewood et al. 2010 [49]	lcl_bal(9) vs. lcl_ctrl(13)
		lcl_unbal(4) vs. lcl_ctrl(13)
GSE12679	Harris et al. 2008 [50]	endothelial(16) vs. neuron(16)
GSE13274	Hartman et al. 2010 [51]	HMEC_ki(5) vs. HMEC_ctrl(5)
		HMEC_ki(5) vs. HMEC_wt(5)
		HMEC_wt(5) vs. HMEC_ctrl(5)
GSE4498	Harvey et al. 2007 [52]	smoker(10) vs. non-smoker(12)
GSE23994	Herbert et al. 2010 [53]	e_50(4) vs. e_IUSM(4)
		e_50(4) vs. e_normal(4)
		e_IUSM(4) vs. e_normal(4)
		s_50(4) vs. s_IUSM(4)
		s_50(4) vs. s_normal(4)
		s_IUSM(4) vs. s_normal(4)
GSE7305	Hever et al. 2007 [54]	disease(10) vs. normal(10)
GSE19650	Hiraoka et al. 2011 [55]	IPMA(6) vs. normal(7)
		IPMC(6) vs. normal(7)
		invasive(3) vs. normal(7)
GSE4107	Hong et al. 2007 [56]	patient(12) vs. ctrl(10)
GSE9212	Hsu et al. 2009 [57]	NKX2(6) vs. ctrl(6)
		PAX9(6) vs. ctrl(6)
		TTF1(6) vs. ctrl(6)
GSE6885	Ince et al. 2007 [58]	BPLER(6) vs. BPE(5)
		HMLER(6) vs. HME(4)

Accession no.	Reference	Contrast
GSE10586	Jailwala et al. 2009 [59]	T1D(12) vs. ctrl(15)
GSE32473	Jensen et al. 2012 [60]	beta(10) vs. baseline(10)
		pime(10) vs. baseline(10)
GSE10700	Jorgensen et al. 2008 [61]	2R4F_02h(4) vs. m_02h(4)
		2R4F_04h(4) vs. m_04h(4)
		2R4F_08h(4) vs. m_08h(4)
		2R4F_24h(4) vs. m_24h(4)
		l_02h(4) vs. m_02h(4)
		l_04h(4) vs. m_04h(4)
		l_08h(4) vs. m_08h(4)
		l_24h(4) vs. m_24h(4)
GSE13067	Jorissen et al. 2008 [62]	MSS(63) vs. MSI(11)
GSE13294		MSS(77) vs. MSI(78)
GSE4567	Karoly et al. 2007 [63]	UFP(4) vs. ctrl(4)
GSE8823	Kazeros et al. 2008 [64]	sm(13) vs. non-sm(11)
GSE21942	Kemppinen et al. 2011 [65]	patient(10) vs. ctrl(15)
GSE30355	Kennedy-Crispin et al. 2012 [66]	KC(10) vs. NHEK(10)
		LCM(10) vs. NHEK(10)
GSE24468	Kobayashi et al. 2010 [67]	R5020(10) vs. vehicle(10)
GSE6519	Kothapalli et al. 2007 [68]	L(4) vs. ctrl(4)
		L3(4) vs. ctrl(4)
GSE13670	Koziel et al. 2009 [69]	hMDM_08h(5) vs. ctrl_08h(5)
		hMDM_24h(5) vs. ctrl_24h(5)
		hMDM_48h(5) vs. ctrl_48h(5)
GSE21422	Kretschmer et al. 2011 [70]	DCIS(9) vs. healthy(5)
		IDC(5) vs. healthy(5)
GSE10245	Kuner et al. 2009 [71]	NSCLC_SCC(18) vs. NSCLC_AC(40)
GSE14924	Le et al. 2009 [72]	AML_CD4(10) vs. health_CD4(10)
		AML_CD8(10) vs. health_CD8(11)
GSE27390	Lee et al. 2011 [73]	RA(9) vs. OA(10)
GSE7621	Lesnick et al. 2007 [74]	PD(16) vs. normal(9)
GSE20891	Leung et al. 2011 [75]	sdf(4) vs. ctrl(4)
GSE15372	Li et al. 2009 [76]	round5_A2780(5) vs. parental_A2780(5)
GSE5281	Liang et al. 2007 [77]	EC_affected(10) vs. EC_ctrl(13)
		HIP_affected(10) vs. HIP_ctrl(13)
		MTG_affected(16) vs. MTG_ctrl(12)
		PC_affected(9) vs. PC_ctrl(13)
		SFG_affected(23) vs. SFG_ctrl(11)
		VCX_affected(19) vs. VCX_ctrl(12)
GSE10479	Liu et al. 2008 [78]	treated(4) vs. ctrl(4)
GSE18608	Liu et al. 2010 [79]	SG_A(10) vs. SG_H(4)
		SG_B(10) vs. SG_H(4)
GSE26272	Liu et al. 2011 [80]	ZNF145(4) vs. ctrl(4)
GSE20910	Loudin et al. 2011 [81]	ALL_DS(23) vs. ALL_NDS(26)
GSE6962	Magda et al. 2008 [82]	PCI5002(4) vs. ctrl(4)
		PCI5003(4) vs. ctrl(4)
GSE12121	Mans et al. 2009 [83]	Pg(4) vs. sham(4)
		Pg_Sg(4) vs. Pg(4)
		Pg_Sg(4) vs. sham(4)
		Sg(4) vs. sham(4)
GSE11430	Maouche et al. 2008 [84]	mono(5) vs. macro(5)
GSE30201	McGowan et al. 2011 [85]	patient(8) vs. normal(11)
GSE13987	Meyers et al. 2009 [86]	B_rolipram(4) vs. B_ctrl(4)
		CLL_rolipram(4) vs. CLL_ctrl(4)
		T_rolipram(4) vs. T_ctrl(4)
GSE7161	Miao et al. 2007 [87]	HCT116(4) vs. HCT116_ctrl(4)
		MCF7(2) vs. MCF7_ctrl(2)
GSE26966	Michaelis et al. 2011 [88]	tumor(14) vs. normal(9)

Accession no.	Reference	Contrast
GSE12949	Mikkelsen et al. 2009 [89]	1h(4) vs 0h(4)
		8h(4) vs 0h(4)
		8h(4) vs 1h(4)
GSE23343	Misu et al. 2010 [90]	hyper(10) vs. normo(7)
GSE6054	Mosig et al. 2008 [91]	heterozygot_FH(6) vs. ctrl(13)
		homozygot_FH(4) vs. ctrl(13)
GSE20211	Mowery et al. 2010 [92]	LMP_420(13) vs. ctrl(13)
GSE11869	Naciff et al. 2009 [93]	high_08h(5) vs. ctrl_08h(5)
		high_24h(5) vs. ctrl_24h(5)
		high_48h(5) vs. ctrl_48h(5)
		low_08h(5) vs. ctrl_08h(5)
		low_24h(5) vs. ctrl_24h(5)
		low_48h(5) vs. ctrl_48h(5)
		veryhigh_08h(5) vs. ctrl_08h(5)
		veryhigh_24h(5) vs. ctrl_24h(5)
		veryhigh_48h(5) vs. ctrl_48h(5)
		verylow_08h(5) vs. ctrl_08h(5)
		verylow_24h(5) vs. ctrl_24h(5)
		verylow_48h(5) vs. ctrl_48h(5)
GSE4182	Nagy et al. 2006 [94]	test(4) vs. ctrl(5)
GSE26511	Noordhuis et al. 2011 [95]	positive(19) vs. negative(20)
GSE9250	Ouillette et al. 2008 [96]	del13q(10) vs. FISHnormal(10)
GSE32719	Pang et al. 2012 [97]	middle(5) vs. young(14)
		old(8) vs. young(14)
GSE22459	Park et al. 2010 [98]	IFTA(24) vs. norm(25)
		IFTA_i(16) vs. norm(25)
GSE6281	Pedersen et al. 2007 [99]	ctrl_07h(5) vs. ctrl_00h(3)
		ctrl_48h(4) vs. ctrl_00h(3)
		ctrl_96h(4) vs. ctrl_00h(3)
		p_07h(5) vs. p_00h(4)
		p_48h(3) vs. p_00h(4)
		p_96h(6) vs. p_00h(4)
GSE10810	Pedraza et al. 2010 [100]	tumor(31) vs. ctrl(27)
GSE12056	Pellegrini et al. 2008 [101]	knock_out(10) vs. ctrl(10)
GSE7462	Peretz et al. 2007 [102]	DE_00h(5) vs. FA_00h(4)
		DE_04h(5) vs. FA_04h(4)
		DE_22h(3) vs. FA_22h(2)
GSE22435	Pihlajamaki et al. 2011 [103]	IGT_DM2(7) vs. ctrl(10)
GSE6872	Platts et al. 2007 [104]	teratozoo(8) vs. normo(13)
GSE11348	Proud et al. 2007 [105]	ctrl_2d(16) vs. ctrl_pre(16)
		ctrl_8h(16) vs. ctrl_pre(16)
		rv16_2d(15) vs. rv16_pre(15)
		rv16_8h(15) vs. rv16_pre(15)
GSE11524	Raghavachari et al. 2007 [106]	sickle(18) vs. ctrl(12)
GSE7116	Raje et al. 2008 [107]	MM(10) vs. healthy(5)
		ONJ(11) vs. healthy(5)
GSE12773	Rate et al. 2009 [108]	AEC_conditioned(5) vs. ctrl(5)
GSE31177	Rayner et al. 2011 [109]	treated_04w(6) vs. ctrl_04w(6)
		treated_12w(6) vs. ctrl_12w(6)
GSE19810	Riewe et al. 2010 [110]	pg(4) vs. ctrl(4)
GSE31215	Riggi et al. 2010 [111]	EWS_FLI(4) vs. empty_vector(4)
GSE8762	Runne et al. 2010 [112]	HD_f(8) vs. ctrl_f(5)
		HD_m(4) vs. ctrl_m(5)
GSE22842	Saddic et al. 2010 [113]	rb(4) vs. ctrl(4)
		rb_smyd(4) vs. ctrl(4)
		smyd(4) vs. ctrl(4)
GSE26526	Saiya-Cork et al. 2011 [114]	del11q(10) vs. n_del11q(9)
GSE7554	Samols et al. 2007 [115]	293_CV(4) vs. 293_miRNA(4)
GSE13284	Sankaran et al. 2008 [116]	B(6) vs. N(6)
GSE5563	Santegoets et al. 2007 [117]	VIN_b(9) vs. ctrl_b(10)

Accession no.	Reference	Contrast
GSE32057	Sen et al. 2011 [118]	SDS(9) vs. normal(7)
GSE7846	Sha et al. 2007 [119]	em(5) vs. without_em(5)
GSE11959	Shang et al. 2008 [120]	24h_10H5(4) vs. vehicle(4)
GSE9264	Skalsky et al. 2007 [121]	miR_155(4) vs. pcDNA3_1(4)
		miR_k12(4) vs. pcDNA3_1(4)
GSE6798	Skov et al. 2007 [122]	PCOS(16) vs. ctrl(13)
GSE3292	Slebos et al. 2006 [123]	positive(8) vs. negative(28)
GSE7890	Smith et al. 2008 [124]	keloid_t(5) vs. normal_t(4)
		keloid_u(5) vs. normal_u(5)
GSE11886	Smith et al. 2008 [125]	patient_IFN(7) vs. ctrl_IFN(9)
		patient_none(8) vs. ctrl_none(9)
GSE7869	Song et al. 2009 [126]	small+medium+large(13) vs. mini(5)
GSE12368	Soon et al. 2009 [127]	ACA(16) vs. normal(6)
		ACC(12) vs. ACA(16)
		ACC(12) vs. normal(6)
GSE22600	Spillman et al. 2010 [128]	2008_tx(4) vs. 2008_ctrl(4)
		PEO4_tx(4) vs. PEO4_ctrl(3)
GSE12734	Stamatopoulos et al. 2009 [129]	high(7) vs. low(7)
GSE32924	Suarez-Farinas et al. 2011 [130]	AL(13) vs. normal(8)
		ANL(12) vs. normal(8)
GSE4290	Sun et al. 2006 [131]	grade_2(45) vs. non-tumor(23)
		grade_3(31) vs. non-tumor(23)
		grade_4(81) vs. non-tumor(23)
GSE33316	Sun et al. 2012 [132]	castrated(5) vs. ctrl(5)
GSE28750	Sutherland et al. 2011 [133]	post_s(11) vs. healthy(20)
		sepsis(10) vs. healthy(20)
		sepsis(10) vs. post_s(11)
GSE19982	Tan et al. 2010 [134]	C(15) vs. O(15)
GSE11199	Thuong et al. 2008 [135]	LTB_stim(4) vs. PTB_stim(4)
		LTB_stim(4) vs. TBM_stim(4)
		PTB_stim(4) vs. TBM_stim(4)
		stim(12) vs. unstim(12)
GSE31773	Tsitsiou et al. 2012 [136]	CD4+_sa(8) vs. CD4+_he(8)
		CD4+_sa(8) vs. CD4+_nsa(4)
		CD8+_sa(8) vs. CD8+_he(8)
		CD8+_sa(8) vs. CD8+_nsa(4)
GSE14001	Tung et al. 2009 [137]	high(10) vs. surface(3)
		low(10) vs. high(10)
		low(10) vs. surface(3)
GSE5764	Turashvili et al. 2007 [138]	tumor_d(5) vs. normal_d(10)
		tumor_d(5) vs. normal_d+normal_l(20)
		tumor_d(5) vs. normal_l(10)
		tumor_l(5) vs. normal_d(10)
		tumor_l(5) vs. normal_d+normal_l(20)
		tumor_l(5) vs. normal_l(10)
GSE13933	Turetz et al. 2009 [139]	sa_non-sm(23) vs. t_non-sm(26)
		sa_sm(19) vs. t_sm(19)
		t_sm(19) vs. t_non-sm(26)
GSE7788	Van et al. 2010 [140]	NLPHL(10) vs. THRBL(10)
GSE26725	Vargova et al. 2011 [141]	B_CLL(12) vs. ctrl(5)
GSE4488	Vierimaa et al. 2006 [142]	case(9) vs. ctrl(7)
GSE7014	Vihola et al. 2010 [143]	DM1(10) vs. normal(6)
		DM2(20) vs. normal(6)
GSE7562	Vivanco et al. 2007 [144]	A431_W(2) vs. A431_P(2)
		HCC827_W(2) vs. HCC827_P(2)
		SKBR3_W(2) vs. SKBR3_P(2)
		WT(6) vs. PR(6)
GSE9150	Vroling et al. 2008 [145]	allergic_HDM(5) vs. allergic_saline(5)
		ctrl_HDM(5) vs. ctrl_saline(5)

Accession no.	Reference	Contrast
GSE24795	Wilding et al. 2010 [146]	rer_pos(14) vs. rer_neg(16)
GSE27659	Wong et al. 2010 [147]	braf(5) vs. wt(5)
GSE5850	Wood et al. 2007 [148]	NL(6) vs. P(6)
GSE4302	Woodruff et al. 2007 [149]	asthmatic_b(42) vs. healthy(28)
		asthmatic_f(19) vs. asthmatic_b(42)
		asthmatic_p(13) vs. asthmatic_b(42)
		smoker(16) vs. healthy(28)
GSE7807	Woszczek et al. 2008 [150]	LTD4(4) vs. ctrl(4)
GSE27187	Yan et al. 2011 [151]	mutant(6) vs. wild_type(4)
GSE11981	Yauch et al. 2008 [152]	HhAntag(4) vs. vehicle(4)
GSE20504	Zeisberger et al. 2010 [153]	serum(5) vs. serum_free(5)

References

[1] Adam O, Lavall D, Theobald K, Hohl M, Grube M, et al. (2010) Rac1-induced connective tissue growth factor regulates connexin 43 and n-cadherin expression in atrial fibrillation. Journal of the American College of Cardiology 55: 469–480.

[2] Akinci MM, Turner H, Taveras M, Barash A, Wang Z, et al. (2009) Molecular profiling of conjunctival epithelial side-population stem cells: atypical cell surface markers and sources of a slow-cycling phenotype. Investigative ophthalmology & visual science 50: 4162–4172.

[3] Aoyagi K, Minashi K, Igaki H, Tachimori Y, Nishimura T, et al. (2011) Artificially induced epithelial-mesenchymal transition in surgical subjects: its implications in clinical and basic cancer research. PloS one 6: e18196.

[4] Arashiro P, Eisenberg I, Kho AT, Cerqueira AM, Canovas M, et al. (2009) Transcriptional regulation differs in affected facioscapulohumeral muscular dystrophy patients compared to asymptomatic related carriers. Proceedings of the National Academy of Sciences 106: 6220–6225.

[5] Arijs I, Li K, Toedter G, Quintens R, Van Lommel L, et al. (2009) Mucosal gene signatures to predict response to infliximab in patients with ulcerative colitis. Gut 58: 1612–1619.

[6] Bae D, Camilli TC, Chun G, Lal M, Wright K, et al. (2009) Bypass of hexavalent chromium-induced growth arrest by a protein tyrosine phosphatase inhibitor: enhanced survival and mutagenesis. Mutation Research/Fundamental and Molecular Mechanisms of Mutagenesis 660: 40–46.

[7] Baeke F, Korf H, Overbergh L, Verstuyf A, Thorrez L, et al. (2011) The vitamin d analog, tx527, promotes a human cd4+ cd25highcd127low regulatory t cell profile and induces a migratory signature specific for homing to sites of inflammation. The Journal of Immunology 186: 132–142.

[8] Baens M, Ferreiro JF, Tousseyn T, Urbankova H, Michaux L, et al. (2012) t (x; 14)(p11. 4; q32. 33) is recurrent in marginal zone lymphoma and up-regulates gpr34. haematologica 97: 184–188.

[9] Bhat-Nakshatri P, Appaiah H, Ballas C, Pick-Franke P, Goulet R, et al. (2010) Slug/snai2 and tumor necrosis factor generate breast cells with cd44+/cd24-phenotype. BMC cancer 10: 411.

[10] Brennan D, McGee S, Rexhepaj E, O'Connor D, Robson M, et al. (2011) Identification of a myometrial molecular profile for dystocic labor. BMC pregnancy and childbirth 11: 74.

[11] Bresolin S, Zecca M, Flotho C, Trentin L, Zangrando A, et al. (2010) Gene expression–based classification as an independent predictor of clinical outcome in juvenile myelomonocytic leukemia. Journal of Clinical Oncology 28: 1919–1927.

[12] Brouard S, Puig-Pey I, Lozano JJ, Pallier A, Braud C, et al. (2010) Comparative transcriptional and phenotypic peripheral blood analysis of kidney recipients under cyclosporin a or sirolimus monotherapy. American Journal of Transplantation 10: 2604–2614.

[13] Burga LN, Tung NM, Troyan SL, Bostina M, Konstantinopoulos PA, et al. (2009) Altered proliferation and differentiation properties of primary mammary epithelial cells from brca1 mutation carriers. Cancer research 69: 1273–1278.

[14] Burney RO, Talbi S, Hamilton AE, Vo KC, Nyegaard M, et al. (2007) Gene expression analysis of endometrium reveals progesterone resistance and candidate susceptibility genes in women with endometriosis. Endocrinology 148: 3814–3826.

[15] Butchar JP, Cremer TJ, Clay CD, Gavrilin MA, Wewers MD, et al. (2008) Microarray analysis of human monocytes infected with francisella tularensis identifies new targets of host response subversion. PloS one 3: e2924.

[16] Buzzeo MP, Yang J, Casella G, Reddy V (2008) A preliminary gene expression profile of acute graft-versus-host disease. Cell transplantation 17: 489–494.

[17] Carolan BJ, Heguy A, Harvey BG, Leopold PL, Ferris B, et al. (2006) Up-regulation of expression of the ubiquitin carboxyl-terminal hydrolase l1 gene in human airway epithelium of cigarette smokers. Cancer research 66: 10729–10740.

[18] Carolan BJ, Harvey BG, De BP, Vanni H, Crystal RG (2008) Decreased expression of intelectin 1 in the human airway epithelium of smokers compared to nonsmokers. The Journal of Immunology 181: 5760–5767.

[19] Caron G, Le Gallou S, Lamy T, Tarte K, Fest T (2009) Cxcr4 expression functionally discriminates centroblasts versus centrocytes within human germinal center b cells. The Journal of Immunology 182: 7595–7602.

[20] Chen C, Méndez E, Houck J, Fan W, Lohavanichbutr P, et al. (2008) Gene expression profiling identifies genes predictive of oral squamous cell carcinoma. Cancer Epidemiology Biomarkers & Prevention 17: 2152–2162.

[21] Chen L, Eberlein M, Alsaaty S, Martinez-Anton A, Barb J, et al. (2011) Cooperative and redundant signaling of leukotriene b4 and leukotriene d4 in human monocytes. Allergy 66: 1304–1311.

[22] Cho JH, Gelinas R, Wang K, Etheridge A, Piper MG, et al. (2011) Systems biology of interstitial lung diseases: integration of mrna and microrna expression changes. BMC medical genomics 4: 8.

[23] Cizkova M, Cizeron-Clairac G, Vacher S, Susini A, Andrieu C, et al. (2010) Gene expression profiling reveals new aspects of pik3ca mutation in eralpha-positive breast cancer: major implication of the wnt signaling pathway. PLoS One 5: e15647.

[24] Connolly K, Mitter R, Muir M, Jodrell D, Guichard S (2009) Stable xiap knockdown clones of hct116 colon cancer cells are more sensitive to trail, taxanes and irradiation in vitro. Cancer chemotherapy and pharmacology 64: 307–316.

[25] Conti A, Scala S, D'Agostino P, Alimenti E, Morelli D, et al. (2007) Wide gene expression profiling of ischemia-reperfusion injury in human liver transplantation. Liver transplantation 13: 99–113.

[26] Creighton CJ, Li X, Landis M, Dixon JM, Neumeister VM, et al. (2009) Residual breast cancers after conventional therapy display mesenchymal as well as tumor-initiating features. Proceedings of the National Academy of Sciences 106: 13820–13825.

[27] Demmer RT, Behle JH, Wolf DL, Handfield M, Kebschull M, et al. (2008) Transcriptomes in healthy and diseased gingival tissues. Journal of periodontology 79: 2112–2124.

[28] Derbali H, Bossé Y, Côté N, Pibarot P, Audet A, et al. (2010) Increased biglycan in aortic valve stenosis leads to the overexpression of phospholipid transfer protein via toll-like receptor 2. The American journal of pathology 176: 2638–2645.

[29] Dodd LE, Sengupta S, Chen IH, den Boon JA, Cheng YJ, et al. (2006) Genes involved in dna repair and nitrosamine metabolism and those located on chromosome 14q32 are dysregulated in nasopharyngeal carcinoma. Cancer Epidemiology Biomarkers & Prevention 15: 2216–2225.

[30] Dusek JA, Otu HH, Wohlhueter AL, Bhasin M, Zerbini LF, et al. (2008) Genomic counter-stress changes induced by the relaxation response. PLoS One 3: e2576.

[31] Dutta SK, Mitra PS, Ghosh S, Zang S, Sonneborn D, et al. (2012) Differential gene expression and a functional analysis of pcb-exposed children: Understanding disease and disorder development. Environment international 40: 143–154.

[32] Erickson DR, Schwarze SR, Dixon JK, Clark CJ, Hersh MA (2008) Differentiation associated changes in gene expression profiles of interstitial cystitis and control urothelial cells. The Journal of urology 180: 2681–2687.

[33] Fall N, Barnes M, Thornton S, Luyrink L, Olson J, et al. (2007) Gene expression profiling of peripheral blood from patients with untreated new-onset systemic juvenile idiopathic arthritis reveals molecular heterogeneity that may predict macrophage activation syndrome. Arthritis & Rheumatism 56: 3793–3804.

[34] Founds SA, Conley YP, Lyons-Weiler JF, Jeyabalan A, Allen Hogge W, et al. (2009) Altered global gene expression in first trimester placentas of women destined to develop preeclampsia. Placenta 30: 15–24.

[35] Frank MB, Wang S, Aggarwal A, Knowlton N, Jiang K, et al. (2009) Disease-associated pathophysiologic structures in pediatric rheumatic diseases show characteristics of scale-free networks seen in physiologic systems: implications for pathogenesis and treatment. BMC medical genomics 2: 9.

[36] Fredriksson K, Tjäder I, Keller P, Petrovic N, Ahlman B, et al. (2008) Dysregulation of mitochondrial dynamics and the muscle transcriptome in icu patients suffering from sepsis induced multiple organ failure. PloS one 3: e3686.

[37] Fujisaki H, Kakuda H, Shimasaki N, Imai C, Ma J, et al. (2009) Expansion of highly cytotoxic human natural killer cells for cancer cell therapy. Cancer research 69: 4010–4017.

[38] Furge KA, Chen J, Koeman J, Swiatek P, Dykema K, et al. (2007) Detection of dna copy number changes and oncogenic signaling abnormalities from gene expression data reveals myc activation in high-grade papillary renal cell carcinoma. Cancer Research 67: 3171–3176.

[39] Galamb O, Sipos F, Solymosi N, Spisák S, Krenács T, et al. (2008) Diagnostic mrna expression patterns of inflamed, benign, and malignant colorectal biopsy specimen and their correlation with peripheral blood results. Cancer Epidemiology Biomarkers & Prevention 17: 2835–2845.

[40] Garaud JC, Schickel JN, Blaison G, Knapp AM, Dembele D, et al. (2011) B cell signature during inactive systemic lupus is heterogeneous: toward a biological dissection of lupus. PLoS One 6: e23900.

[41] Ghorbel MT, Cherif M, Jenkins E, Mokhtari A, Kenny D, et al. (2010) Transcriptomic analysis of patients with tetralogy of fallot reveals the effect of chronic hypoxia on myocardial gene expression. The Journal of thoracic and cardiovascular surgery 140: 337–345.

[42] Ghosh S, Tan F, Yu T, Li Y, Adisa O, et al. (2011) Global gene expression profiling of endothelium exposed to heme reveals an organ-specific induction of cytoprotective enzymes in sickle cell disease. PloS one 6: e18399.

[43] Giordano TJ, Kuick R, Else T, Gauger PG, Vinco M, et al. (2009) Molecular classification and prognostication of adrenocortical tumors by transcriptome profiling. Clinical Cancer Research 15: 668–676.

[44] Gratchev A, Kzhyshkowska J, Kannookadan S, Ochsenreiter M, Popova A, et al. (2008) Activation of a tgf-β-specific multistep gene expression program in mature macrophages requires glucocorticoid-mediated surface expression of tgf-β receptor ii. The Journal of Immunology 180: 6553–6565.

[45] Gregg JP, Lit L, Baron CA, Hertz-Picciotto I, Walker W, et al. (2008) Gene expression changes in children with autism. Genomics 91: 22–29.

[46] Gümüş ZH, Du B, Kacker A, Boyle JO, Bocker JM, et al. (2008) Effects of tobacco smoke on gene expression and cellular pathways in a cellular model of oral leukoplakia. Cancer Prevention Research 1: 100–111.

[47] Gyorffy B, Molnar B, Lage H, Szallasi Z, Eklund AC (2009) Evaluation of microarray preprocessing algorithms based on concordance with rt-pcr in clinical samples. PLoS One 4: e5645.

[48] Hadziselimovic F, Hadziselimovic N, Demougin P, Oakeley E (2011) Testicular gene expression in cryptorchid boys at risk of azoospermia. Sexual Development 5: 49–59.

[49] Harewood L, Schütz F, Boyle S, Perry P, Delorenzi M, et al. (2010) The effect of translocation-induced nuclear reorganization on gene expression. Genome research 20: 554–564.

[50] Harris LW, Wayland M, Lan M, Ryan M, Giger T, et al. (2008) The cerebral microvasculature in schizophrenia: a laser capture microdissection study. PLoS One 3: e3964.

[51] Hartman ZC, Wei J, Osada T, Glass O, Lei G, et al. (2010) An adenoviral vaccine encoding full-length inactivated human her2 exhibits potent immunogenicty and enhanced therapeutic efficacy without oncogenicity. Clinical Cancer Research 16: 1466–1477.

[52] Harvey BG, Heguy A, Leopold PL, Carolan BJ, Ferris B, et al. (2007) Modification of gene expression of the small airway epithelium in response to cigarette smoking. Journal of Molecular Medicine 85: 39–53.

[53] Herbert BS, Chanoux RA, Liu Y, Baenziger PH, Goswami CP, et al. (2010) A molecular signature of normal breast epithelial and stromal cells from li-fraumeni syndrome mutation carriers. Oncotarget 1: 405.

[54] Hever A, Roth RB, Hevezi P, Marin ME, Acosta JA, et al. (2007) Human endometriosis is associated with plasma cells and overexpression of b lymphocyte stimulator. Proceedings of the National Academy of Sciences 104: 12451–12456.

[55] Hiraoka N, Yamazaki-Itoh R, Ino Y, Mizuguchi Y, Yamada T, et al. (2011) Cxcl17 and icam2 are associated with a potential anti-tumor immune response in early intraepithelial stages of human pancreatic carcinogenesis. Gastroenterology 140: 310–321.

[56] Hong Y, Ho KS, Eu KW, Cheah PY (2007) A susceptibility gene set for early onset colorectal cancer that integrates diverse signaling pathways: implication for tumorigenesis. Clinical cancer research 13: 1107–1114.

[57] Hsu DS, Acharya CR, Balakumaran BS, Riedel RF, Kim MK, et al. (2009) Characterizing the developmental pathways ttf-1, nkx2–8, and pax9 in lung cancer. Proceedings of the National Academy of Sciences 106: 5312–5317.

[58] Ince TA, Richardson AL, Bell GW, Saitoh M, Godar S, et al. (2007) Transformation of different human breast epithelial cell types leads to distinct tumor phenotypes. Cancer cell 12: 160–170.

[59] Jailwala P, Waukau J, Glisic S, Jana S, Ehlenbach S, et al. (2009) Apoptosis of cd4+ cd25high t cells in type 1 diabetes may be partially mediated by il-2 deprivation. PloS one 4: e6527.

[60] Jensen J, Scherer A, Wanke C, Bräutigam M, Bongiovanni S, et al. (2012) Gene expression is differently affected by pimecrolimus and betamethasone in lesional skin of atopic dermatitis. Allergy 67: 413–423.

[61] Jorgensen E, Stinson A, Shan L, Yang J, Gietl D, et al. (2008) Cigarette smoke induces endoplasmic reticulum stress and the unfolded protein response in normal and malignant human lung cells. BMC cancer 8: 229.

[62] Jorissen RN, Lipton L, Gibbs P, Chapman M, Desai J, et al. (2008) Dna copy-number alterations underlie gene expression differences between microsatellite stable and unstable colorectal cancers. Clinical Cancer Research 14: 8061–8069.

[63] Karoly ED, Li Z, Dailey LA, Hyseni X, Huang YCT (2007) Up-regulation of tissue factor in human pulmonary artery endothelial cells after ultrafine particle exposure. Environmental health perspectives 115: 535.

[64] Kazeros A, Harvey BG, Carolan BJ, Vanni H, Krause A, et al. (2008) Overexpression of apoptotic cell removal receptor mertk in alveolar macrophages of cigarette smokers. American journal of respiratory cell and molecular biology 39: 747.

[65] Kemppinen A, Kaprio J, Palotie A, Saarela J (2011) Systematic review of genome-wide expression studies in multiple sclerosis. BMJ open 1.

[66] Kennedy-Crispin M, Billick E, Mitsui H, Gulati N, Fujita H, et al. (2011) Human keratinocytes' response to injury upregulates ccl20 and other genes linking innate and adaptive immunity. Journal of Investigative Dermatology 132: 105–113.

[67] Kobayashi S, Stice JP, Kazmin D, Wittmann BM, Kimbrel EA, et al. (2010) Mechanisms of progesterone receptor inhibition of inflammatory responses in cellular models of breast cancer. Molecular Endocrinology 24: 2292–2302.

[68] Kothapalli KS, Anthony JC, Pan BS, Hsieh AT, Nathanielsz PW, et al. (2007) Differential cerebral cortex transcriptomes of baboon neonates consuming moderate and high docosahexaenoic acid formulas. PLoS One 2: e370.

[69] Koziel J, Maciag-Gudowska A, Mikolajczyk T, Bzowska M, Sturdevant DE, et al. (2009) Phagocytosis of staphylococcus aureus by macrophages exerts cytoprotective effects manifested by the upregulation of antiapoptotic factors. PLoS One 4: e5210.

[70] Kretschmer C, Sterner-Kock A, Siedentopf F, Schoenegg W, Schlag PM, et al. (2011) Identification of early molecular markers for breast cancer. Mol Cancer 10: 15.

[71] Kuner R, Muley T, Meister M, Ruschhaupt M, Buness A, et al. (2009) Global gene expression analysis reveals specific patterns of cell junctions in non-small cell lung cancer subtypes. Lung Cancer 63: 32–38.

[72] Le Dieu R, Taussig DC, Ramsay AG, Mitter R, Miraki-Moud F, et al. (2009) Peripheral blood t cells in acute myeloid leukemia (aml) patients at diagnosis have abnormal phenotype and genotype and form defective immune synapses with aml blasts. Blood 114: 3909–3916.

[73] Lee HM, Sugino H, Aoki C, Shimaoka Y, Suzuki R, et al. (2011) Abnormal networks of immune response-related molecules in bone marrow cells from patients with rheumatoid arthritis as revealed by dna microarray analysis. Arthritis Res Ther 13: R89.

[74] Lesnick TG, Papapetropoulos S, Mash DC, Ffrench-Mullen J, Shehadeh L, et al. (2007) A genomic pathway approach to a complex disease: axon guidance and parkinson disease. PLoS genetics 3: e98.

[75] Leung KT, Chan KYY, Ng PC, Lau TK, Chiu WM, et al. (2011) The tetraspanin cd9 regulates migration, adhesion, and homing of human cord blood cd34+ hematopoietic stem and progenitor cells. Blood 117: 1840–1850.

[76] Li M, Balch C, Montgomery JS, Jeong M, Chung JH, et al. (2009) Integrated analysis of dna methylation and gene expression reveals specific signaling pathways associated with platinum resistance in ovarian cancer. BMC medical genomics 2: 34.

[77] Liang WS, Dunckley T, Beach TG, Grover A, Mastroeni D, et al. (2007) Gene expression profiles in anatomically and functionally distinct regions of the normal aged human brain. Physiological genomics 28: 311–322.

[78] Liu SB, He YY, Zhang Y, Lee WH, Qian JQ, et al. (2008) A novel non-lens $\beta\gamma$- crystallin and trefoil factor complex from amphibian skin and its functional implications. PLoS One 3: e1770.

[79] Liu D, Glaser AP, Patibandla S, Blum A, Munson PJ, et al. (2011) Transcriptional profiling of cd133^{+} cells in coronary artery disease and effects of exercise on gene expression. Cytotherapy 13: 227–236.

[80] Liu TM, Guo XM, Tan HS, Hui JH, Lim B, et al. (2011) Zinc-finger protein 145, acting as an upstream regulator of sox9, improves the differentiation potential of human mesenchymal stem cells for cartilage regeneration and repair. Arthritis & Rheumatism 63: 2711–2720.

[81] Loudin M, Wang J, Leung HE, Gurusiddappa S, Meyer J, et al. (2011) Genomic profiling in down syndrome acute lymphoblastic leukemia identifies histone gene deletions associated with altered methylation profiles. Leukemia 25: 1555–1563.

[82] Magda D, Lecane P, Wang Z, Hu W, Thiemann P, et al. (2008) Synthesis and anticancer properties of water-soluble zinc ionophores. Cancer research 68: 5318–5325.

[83] Mans JJ, von Lackum K, Dorsey C, Willis S, Wallet SM, et al. (2009) The degree of microbiome complexity influences the epithelial response to infection. BMC genomics 10: 380.

[84] Maouche S, Poirier O, Godefroy T, Olaso R, Gut I, et al. (2008) Performance comparison of two microarray platforms to assess differential gene expression in human monocyte and macrophage cells. BMC genomics 9: 302.

[85] McGowan KA, Pang WW, Bhardwaj R, Perez MG, Pluvinage JV, et al. (2011) Reduced ribosomal protein gene dosage and p53 activation in low-risk myelodysplastic syndrome. Blood 118: 3622–3633.

[86] Meyers JA, Su DW, Lerner A (2009) Chronic lymphocytic leukemia and b and t cells differ in their response to cyclic nucleotide phosphodiesterase inhibitors. The Journal of Immunology 182: 5400–5411.

[87] Miao ZH, Player A, Shankavaram U, Wang YH, Zimonjic DB, et al. (2007) Nonclassic functions of human topoisomerase i: genome-wide and pharmacologic analyses. Cancer research 67: 8752–8761.

[88] Michaelis KA, Knox AJ, Xu M, Kiseljak-Vassiliades K, Edwards MG, et al. (2011) Identification of growth arrest and dna-damage-inducible gene β (gadd45β) as a novel tumor suppressor in pituitary gonadotrope tumors. Endocrinology 152: 3603–3613.

[89] Mikkelsen T, Brodie C, Finniss S, Berens ME, Rennert JL, et al. (2009) Radiation sensitization of glioblastoma by cilengitide has unanticipated schedule-dependency. International Journal of Cancer 124: 2719–2727.

[90] Misu H, Takamura T, Takayama H, Hayashi H, Matsuzawa-Nagata N, et al. (2010) A liver-derived secretory protein, selenoprotein p, causes insulin resistance. Cell metabolism 12: 483–495.

[91] Mosig S, Rennert K, Büttner P, Krause S, Lütjohann D, et al. (2008) Monocytes of patients with familial hypercholesterolemia show alterations in cholesterol metabolism. BMC Medical Genomics 1: 60.

[92] Mowery Y, Weinberg J, Kennedy M, Bond K, Moore J, et al. (2010) Lmp-420: a novel purine nucleoside analog with potent cytotoxic effects for cll cells and minimal toxicity for normal hematopoietic cells. Leukemia 24: 1580–1587.

[93] Naciff JM, Khambatta ZS, Thomason RG, Carr GJ, Tiesman JP, et al. (2009) The genomic response of a human uterine endometrial adenocarcinoma cell line to 17α-ethynyl estradiol. Toxicological sciences 107: 40–55.

[94] Nagy GR, Gyõrffy B, Galamb O, Molnár B, Nagy B, et al. (2006) Use of routinely collected amniotic fluid for whole-genome expression analysis of polygenic disorders. Clinical chemistry 52: 2013–2020.

[95] Noordhuis MG, Fehrmann RS, Wisman GBA, Nijhuis ER, van Zanden JJ, et al. (2011) Involvement of the tgf-β and β-catenin pathways in pelvic lymph node metastasis in early-stage cervical cancer. Clinical Cancer Research 17: 1317–1330.

[96] Ouillette P, Erba H, Kujawski L, Kaminski M, Shedden K, et al. (2008) Integrated genomic profiling of chronic lymphocytic leukemia identifies subtypes of deletion 13q14. Cancer research 68: 1012–1021.

[97] Pang WW, Price EA, Sahoo D, Beerman I, Maloney WJ, et al. (2011) Human bone marrow hematopoietic stem cells are increased in frequency and myeloid-biased with age. Proceedings of the National Academy of Sciences 108: 20012–20017.

[98] Park WD, Griffin MD, Cornell LD, Cosio FG, Stegall MD (2010) Fibrosis with inflammation at one year predicts transplant functional decline. Journal of the American Society of Nephrology 21: 1987–1997.

[99] Pedersen MB, Skov L, Menné T, Johansen JD, Olsen J (2007) Gene expression time course in the human skin during elicitation of allergic contact dermatitis. Journal of Investigative Dermatology 127: 2585–2595.

[100] Pedraza V, Gomez-Capilla JA, Escaramis G, Gomez C, Torné P, et al. (2010) Gene expression signatures in breast cancer distinguish phenotype characteristics, histologic subtypes, and tumor invasiveness. Cancer 116: 486–496.

[101] Pellegrini M, Cheng JC, Voutila J, Judelson D, Taylor J, et al. (2008) Expression profile of creb knockdown in myeloid leukemia cells. BMC cancer 8: 264.

[102] Peretz A, Peck EC, Bammler TK, Beyer RP, Sullivan JH, et al. (2007) Diesel exhaust inhalation and assessment of peripheral blood mononuclear cell gene transcription effects: an exploratory study of healthy human volunteers. Inhalation toxicology 19: 1107–1119.

[103] Pihlajamäki J, Lerin C, Itkonen P, Boes T, Floss T, et al. (2011) Expression of the splicing factor gene *SFRS10* is reduced in human obesity and contributes to enhanced lipogenesis. Cell metabolism 14: 208–218.

[104] Platts AE, Dix DJ, Chemes HE, Thompson KE, Goodrich R, et al. (2007) Success and failure in human spermatogenesis as revealed by teratozoospermic rnas. Human molecular genetics 16: 763–773.

[105] Proud D, Turner RB, Winther B, Wiehler S, Tiesman JP, et al. (2008) Gene expression profiles during in vivo human rhinovirus infection insights into the host response. American journal of respiratory and critical care medicine 178: 962–968.

[106] Raghavachari N, Xu X, Harris A, Villagra J, Logun C, et al. (2007) Amplified expression profiling of platelet transcriptome reveals changes in arginine metabolic pathways in patients with sickle cell disease. Circulation 115: 1551–1562.

[107] Raje N, Woo SB, Hande K, Yap JT, Richardson PG, et al. (2008) Clinical, radiographic, and biochemical characterization of multiple myeloma patients with osteonecrosis of the jaw. Clinical Cancer Research 14: 2387–2395.

[108] Rate A, Upham JW, Bosco A, McKenna KL, Holt PG (2009) Airway epithelial cells regulate the functional phenotype of locally differentiating dendritic cells: implications for the pathogenesis of infectious and allergic airway disease. The Journal of Immunology 182: 72–83.

[109] Rayner KJ, Esau CC, Hussain FN, McDaniel AL, Marshall SM, et al. (2011) Inhibition of mir-33a/b in non-human primates raises plasma hdl and lowers vldl triglycerides. Nature 478: 404–407.

[110] Riewe S, Mans J, Hirano T, Katz J, Shiverick K, et al. (2010) Human trophoblast responses to porphyromonas gingivalis infection. Molecular oral microbiology 25: 252–259.

[111] Riggi N, Suvà ML, De Vito C, Provero P, Stehle JC, et al. (2010) Ews-fli-1 modulates mirna145 and sox2 expression to initiate mesenchymal stem cell reprogramming toward ewing sarcoma cancer stem cells. Genes & development 24: 916–932.

[112] Runne H, Kuhn A, Wild EJ, Pratyaksha W, Kristiansen M, et al. (2007) Analysis of potential transcriptomic biomarkers for huntington's disease in peripheral blood. Proceedings of the National Academy of Sciences 104: 14424–14429.

[113] Saddic LA, West LE, Aslanian A, Yates JR, Rubin SM, et al. (2010) Methylation of the retinoblastoma tumor suppressor by smyd2. Journal of Biological Chemistry 285: 37733–37740.

[114] Saiya-Cork K, Collins R, Parkin B, Ouillette P, Kuizon E, et al. (2011) A pathobiological role of the insulin receptor in chronic lymphocytic leukemia. Clinical Cancer Research 17: 2679–2692.

[115] Samols MA, Skalsky RL, Maldonado AM, Riva A, Lopez MC, et al. (2007) Identification of cellular genes targeted by kshv-encoded micrornas. PLoS pathogens 3: e65.

[116] Sankaran VG, Menne TF, Xu J, Akie TE, Lettre G, et al. (2008) Human fetal hemoglobin expression is regulated by the developmental stage-specific repressor bcl11a. Science 322: 1839–1842.

[117] Santegoets LA, Seters Mv, Helmerhorst TJ, Heijmans-Antonissen C, Hanifi-Moghaddam P, et al. (2007) Hpv related vin: highly proliferative and diminished responsiveness to extracellular signals. International journal of cancer 121: 759–766.

[118] Sen S, Wang H, Nghiem CL, Zhou K, Yau J, et al. (2011) The ribosome-related protein, sbds, is critical for normal erythropoiesis. Blood 118: 6407–6417.

[119] Sha G, Wu D, Zhang L, Chen X, Lei M, et al. (2007) Differentially expressed genes in human endometrial endothelial cells derived from eutopic endometrium of patients with endometriosis compared with those from patients without endometriosis. Human reproduction 22: 3159–3169.

[120] Shang Y, Mao Y, Batson J, Scales SJ, Phillips G, et al. (2008) Antixenograft tumor activity of a humanized anti-insulin-like growth factor-i receptor monoclonal antibody is associated with decreased akt activation and glucose uptake. Molecular cancer therapeutics 7: 2599–2608.

[121] Skalsky RL, Samols MA, Plaisance KB, Boss IW, Riva A, et al. (2007) Kaposi's sarcoma-associated herpesvirus encodes an ortholog of mir-155. Journal of virology 81: 12836–12845.

[122] Skov V, Glintborg D, Knudsen S, Jensen T, Kruse TA, et al. (2007) Reduced expression of nuclear-encoded genes involved in mitochondrial oxidative metabolism in skeletal muscle of insulin-resistant women with polycystic ovary syndrome. Diabetes 56: 2349–2355.

[123] Slebos RJ, Yi Y, Ely K, Carter J, Evjen A, et al. (2006) Gene expression differences associated with human papillomavirus status in head and neck squamous cell carcinoma. Clinical cancer research 12: 701–709.

[124] Smith JC, Boone BE, Opalenik SR, Williams SM, Russell SB (2007) Gene profiling of keloid fibroblasts shows altered expression in multiple fibrosis-associated pathways. Journal of Investigative Dermatology 128: 1298–1310.

[125] Smith JA, Barnes MD, Hong D, DeLay ML, Inman RD, et al. (2008) Gene expression analysis of macrophages derived from ankylosing spondylitis patients reveals interferon-γ dysregulation. Arthritis & Rheumatism 58: 1640–1649.

[126] Song X, Di Giovanni V, He N, Wang K, Ingram A, et al. (2009) Systems biology of autosomal dominant polycystic kidney disease (adpkd): computational identification of gene expression pathways and integrated regulatory networks. Human molecular genetics 18: 2328–2343.

[127] Soon P, Gill A, Benn D, Clarkson A, Robinson B, et al. (2009) Microarray gene expression and immunohistochemistry analyses of adrenocortical tumors identify igf2 and ki-67 as useful in differentiating carcinomas from adenomas. Endocrine-related cancer 16: 573–583.

[128] Spillman MA, Manning NG, Dye WW, Sartorius CA, Post MD, et al. (2010) Tissue-specific pathways for estrogen regulation of ovarian cancer growth and metastasis. Cancer research 70: 8927–8936.

[129] Stamatopoulos B, Haibe-Kains B, Equeter C, Meuleman N, Sorée A, et al. (2009) Gene expression profiling reveals differences in microenvironment interaction between patients with chronic lymphocytic leukemia expressing high versus low zap70 mrna. haematologica 94: 790–799.

[130] Suárez-Fariñas M, Tintle SJ, Shemer A, Chiricozzi A, Nograles K, et al. (2011) Nonlesional atopic dermatitis skin is characterized by broad terminal differentiation defects and variable immune abnormalities. Journal of Allergy and Clinical Immunology 127: 954–964.

[131] Sun L, Hui AM, Su Q, Vortmeyer A, Kotliarov Y, et al. (2006) Neuronal and glioma-derived stem cell factor induces angiogenesis within the brain. Cancer cell 9: 287–300.

[132] Sun Y, Wang BE, Leong KG, Yue P, Li L, et al. (2012) Androgen deprivation causes epithelial–mesenchymal transition in the prostate: implications for androgen-deprivation therapy. Cancer research 72: 527–536.

[133] Sutherland A, Thomas M, Brandon RA, Brandon RB, Lipman J, et al. (2011) Development and validation of a novel molecular biomarker diagnostic test for the early detection of sepsis. Crit Care 15: R149.

[134] Tan MH, Wong C, Tan H, Yang X, Ditlev J, et al. (2010) Genomic expression and single-nucleotide polymorphism profiling discriminates chromophobe renal cell carcinoma and oncocytoma. BMC cancer 10: 196.

[135] Thuong NTT, Dunstan SJ, Chau TTH, Thorsson V, Simmons CP, et al. (2008) Identification of tuberculosis susceptibility genes with human macrophage gene expression profiles. PLoS pathogens 4: e1000229.

[136] Tsitsiou E, Williams AE, Moschos SA, Patel K, Rossios C, et al. (2012) Transcriptome analysis shows activation of circulating $cd8^{+}$ t cells in patients with severe asthma. Journal of Allergy and Clinical Immunology 129: 95–103.

[137] Tung CS, Mok SC, Tsang YT, Zu Z, Song H, et al. (2009) Pax2 expression in low malignant potential ovarian tumors and low-grade ovarian serous carcinomas. Modern Pathology 22: 1243–1250.

[138] Turashvili G, Bouchal J, Baumforth K, Wei W, Dziechciarkova M, et al. (2007) Novel markers for differentiation of lobular and ductal invasive breast carcinomas by laser microdissection and microarray analysis. BMC cancer 7: 55.

[139] Turetz ML, OConnor TP, Tilley AE, Strulovici-Barel Y, Salit J, et al. (2009) Trachea epithelium as a canary for cigarette smoking-induced biologic phenotype of the small airway epithelium. Clinical and translational science 2: 260–272.

[140] Van Loo P, Tousseyn T, Vanhentenrijk V, Dierickx D, Malecka A, et al. (2010) T-cell/histiocyte-rich large b-cell lymphoma shows transcriptional features suggestive of a tolerogenic host immune response. haematologica 95: 440–448.

[141] Vargova K, Curik N, Burda P, Basova P, Kulvait V, et al. (2011) Myb transcriptionally regulates the mir-155 host gene in chronic lymphocytic leukemia. Blood 117: 3816–3825.

[142] Vierimaa O, Georgitsi M, Lehtonen R, Vahteristo P, Kokko A, et al. (2006) Pituitary adenoma predisposition caused by germline mutations in the aip gene. Science 312: 1228–1230.

[143] Vihola A, Bachinski LL, Sirito M, Olufemi SE, Hajibashi S, et al. (2010) Differences in aberrant expression and splicing of sarcomeric proteins in the myotonic dystrophies dm1 and dm2. Acta neuropathologica 119: 465–479.

[144] Vivanco I, Palaskas N, Tran C, Finn SP, Getz G, et al. (2007) Identification of the jnk signaling pathway as a functional target of the tumor suppressor pten. Cancer cell 11: 555–569.

[145] Vroling AB, Jonker MJ, Luiten S, Breit TM, Fokkens WJ, et al. (2008) Primary nasal epithelium exposed to house dust mite extract shows activated expression in allergic individuals. American journal of respiratory cell and molecular biology 38: 293–299.

[146] Wilding JL, McGowan S, Liu Y, Bodmer WF (2010) Replication error deficient and proficient colorectal cancer gene expression differences caused by 3' utr polyt sequence deletions. Proceedings of the National Academy of Sciences 107: 21058–21063.

[147] Wong KK, Tsang Y, Deavers MT, Mok SC, Zu Z, et al. (2010) Braf mutation is rare in advanced-stage low-grade ovarian serous carcinomas. The American journal of pathology 177: 1611–1617.

[148] Wood JR, Dumesic DA, Abbott DH, Strauss JF (2007) Molecular abnormalities in oocytes from women with polycystic ovary syndrome revealed by microarray analysis. Journal of Clinical Endocrinology & Metabolism 92: 705–713.

[149] Woodruff PG, Boushey HA, Dolganov GM, Barker CS, Yang YH, et al. (2007) Genome-wide profiling identifies epithelial cell genes associated with asthma and with treatment response to corticosteroids. Proceedings of the National Academy of Sciences 104: 15858–15863.

[150] Woszczek G, Chen LY, Nagineni S, Kern S, Barb J, et al. (2008) Leukotriene d_4 induces gene expression in human monocytes through cysteinyl leukotriene type i receptor. Journal of Allergy and Clinical Immunology 121: 215–221.

[151] Yan XJ, Xu J, Gu ZH, Pan CM, Lu G, et al. (2011) Exome sequencing identifies somatic mutations of dna methyltransferase gene dnmt3a in acute monocytic leukemia. Nature genetics 43: 309–315.

[152] Yauch RL, Gould SE, Scales SJ, Tang T, Tian H, et al. (2008) A paracrine requirement for hedgehog signalling in cancer. Nature 455: 406–410.

[153] Zeisberger SM, Zoller S, Riegel M, Chen S, Krenning G, et al. (2010) Optimization of the culturing conditions of human umbilical cord blood-derived endothelial colony-forming cells under xeno-free conditions applying a transcriptomic approach. Genes to Cells 15: 671–687.
