# Supplementary material for: Method Designed to Respect Molecular Heterogeneity Can Profoundly Correct Present Data Interpretations for Genome-Wide Expression Analysis
Source: PLoS One. 2015 Mar 20;10(3):e0121154. doi: 10.1371/journal.pone.0121154 (PMC4368820; doi:10.1371/journal.pone.0121154)
Supplement: S2 Table — (PDF) [file pone.0121154.s024.pdf]

Data set id.	Reference	Brain region(s)	No. of subjects (PTN:CTL)	Platform
Schizophrenia				
AltarA	SMRI	FC (BA46)	32:34	HG-U133A
AltarC	SMRI	FC (BA46/10)	11:11	HG-U133A
Bahn	SMRI	FC (BA46)	34:33	HG-U133A
Barnes	Barnes et al. [1]	TC (BA22)	23:19	HG-U133 Plus 2.0
Iwa_BA10	Iwamoto et al. [2]	PFC (BA10)	13:15	HG-U95Av2
Iwa_BA46	Iwamoto et al. [3]	FC (BA46)	35:34	HG-U133A
Maycox	Maycox et al. [4]	PFC (BA10)	28:23	HG-U133 Plus 2.0
McLean	Glatt et al. [5]	PFC (BA9)	19:26	HG-U133A
Bipolar disorder				
AltarA	SMRI	FC (BA46)	31:34	HG-U133A
Bahn	SMRI	FC (BA46)	31:33	HG-U133A
Cing	HBTRC	CC	15:21	HG-U133A
Dobrin	SMRI	FC (BA46)	26:25	HG-U133 Plus 2.0
Iwamoto	Iwamoto et al. [3]	FC (BA46)	32:34	HG-U133A
Ryan_D	Ryan et al. [6]	DPFC (BA46)	30:31	HG-U133A
Ryan_O	Ryan et al. [6]	OFC	10:11	HG-U133A
SklarA	SMRI	FC (BA8/9)	11:12	HG-U95Av2
SklarB	SMRI	CB	11:10	HG-U95Av2
Parkinson's disease				
Lesnick	Lesnick et al. [7]	SN	16:9	HG-U133 Plus 2.0
Lewan_D	Lewandowski et al. [8]	DMNV	6:5	HG-U133A 2.0
Lewan_I	Lewandowski et al. [8]	ION	6:5	HG-U133A 2.0
Zhang_B	Zhang et al. [9]	PFC (BA9)	14:15	HG-U133A
Zhang_P	Zhang et al. [9]	PT	15:20	HG-U133A
Zhang_S	Zhang et al. [9]	SN	11:18	HG-U133A
Zheng_1	Zheng et al. [10]	SN	10:8	HG-U133 Plus 2.0
Zheng_2	Zheng et al. [10]	SN	8:9	HG-U133A

Abbreviations: CB, cerebellum; CC, cingulate cortex; CTL, control; DMNV, dorsal motor nucleus of the vagus; DPFC, dorsolateral prefrontal cortex; FC, frontal cortex; HBTRC, Harvard Brain Tissue Resource Center; OFC, orbital frontal cortex; ION, inferior olivary nucleus; PFC, prefrontal cortex; PT, putamen; PTN, patient; SMRI, Stanley Medical Research Institute; SN, substantia nigra; TC, temporal cortex.

References

[1] Barnes MR, Huxley-Jones J, Maycox PR, Lennon M, Thornber A, et al. (2011) Transcription and pathway analysis of the superior temporal cortex and anterior prefrontal cortex in schizophrenia. Journal of neuroscience research 89: 1218–1227.

[2] Iwamoto K, Kakiuchi C, Bundo M, Ikeda K, Kato T (2004) Molecular characterization of bipolar disorder by comparing gene expression profiles of postmortem brains of major mental disorders. Molecular psychiatry 9: 406–416.

[3] Iwamoto K, Bundo M, Kato T (2005) Altered expression of mitochondria-related genes in postmortem brains of patients with bipolar disorder or schizophrenia, as revealed by large-scale dna microarray analysis. Human molecular genetics 14: 241–253.

[4] Maycox P, Kelly F, Taylor A, Bates S, Reid J, et al. (2009) Analysis of gene expression in two large schizophrenia cohorts identifies multiple changes associated with nerve terminal function. Molecular psychiatry 14: 1083–1094.

[5] Glatt SJ, Everall IP, Kremen WS, Corbeil J, Šášik R, et al. (2005) Comparative gene expression analysis of blood and brain provides concurrent validation of selenbp1 up-regulation in schizophrenia. Proceedings of the National Academy of Sciences of the United States of America 102: 15533–15538.

[6] Ryan M, Lockstone H, Huffaker S, Wayland M, Webster M, et al. (2006) Gene expression analysis of bipolar disorder reveals downregulation of the ubiquitin cycle and alterations in synaptic genes. Molecular psychiatry 11: 965–978.

[7] Lesnick TG, Papapetropoulos S, Mash DC, Ffrench-Mullen J, Shehadeh L, et al. (2007) A genomic pathway approach to a complex disease: axon guidance and parkinson disease. PLoS genetics 3: e98.

[8] Lewandowski NM, Ju S, Verbitsky M, Ross B, Geddie ML, et al. (2010) Polyamine pathway contributes to the pathogenesis of parkinson disease. Proceedings of the National Academy of Sciences 107: 16970–16975.

[9] Zhang Y, James M, Middleton FA, Davis RL (2005) Transcriptional analysis of multiple brain regions in parkinson's disease supports the involvement of specific protein processing, energy metabolism, and signaling pathways, and suggests novel disease mechanisms. American Journal of Medical Genetics Part B: Neuropsychiatric Genetics 137: 5–16.

[10] Zheng B, Liao Z, Locascio JJ, Lesniak KA, Roderick SS, et al. (2010) Pgc-1α, a potential therapeutic target for early intervention in parkinsons disease. Science translational medicine 2: 52ra73.
