# Supplementary material for: Method Designed to Respect Molecular Heterogeneity Can Profoundly Correct Present Data Interpretations for Genome-Wide Expression Analysis
Source: PLoS One. 2015 Mar 20;10(3):e0121154. doi: 10.1371/journal.pone.0121154 (PMC4368820; doi:10.1371/journal.pone.0121154)
Supplement: S3 Table — (PDF) [file pone.0121154.s025.pdf]

List id.	Reference	List size	Data analysis method(s)
Schizophrenia			
Arion	Arion et al. [1]	110 genes	HM(p<0.05, |FC|>0.263)
Barnes	Barnes et al. [2]	1998 probe sets	DQC, t-test(p<0.05)
Glatt	Glatt et al. [3]	177 probe sets	t-test(p<0.05)
Maycox	Maycox et al. [4]	1846 probe sets	DQC, t-test(p<0.05)
Mistry	Mistry et al. [5]	136 probe sets	MTA,CFC,t-test(FDR<0.1)
PS	Pérez-Santiago et al. [6]	160 probe sets	MTA,CFC,t-test(80% CF NFP<10)
Bipolar disorder			
Nakatani	Nakatani et al. [7]	108 genes	HM(p<0.05, |FC|>1.5)
Ryan	Ryan et al. [8]	81 genes	DQC,ANCOVA(p<0.05)
Seifu_A	Seifuddin et al. [9]	171 genes	MTA,CFC,HM(p<0.05, |FC|>1.07)
Seifu_F	Seifuddin et al. [9]	141 genes	MTA,CFC,HM(p<0.05, |FC|>1.07)
Seifu_H	Seifuddin et al. [9]	100 genes	MTA,CFC,HM(p<0.05, |FC|>1.07)
Parkinson's disease			
Simunovic	Simunovic et al. [10]	1044 probe sets	SAM, t-test, ANOVA
Zhang_B	Zhang et al. [11]	100 probe sets	HM(p<0.05, FC>1.58 or <-2.09)
Zhang_P	Zhang et al. [11]	100 probe sets	HM(p<0.05, FC>1.33 or <-1.61)
Zhang_S	Zhang et al. [11]	100 probe sets	HM(p<0.05, FC>1.50 or <-1.95)

Abbreviations: CFC, confounding factor control; CF, confidence; DQC, data quality control; FC, fold-change; MTA, meta-analysis; NFP, number of false positives.

References

[1] Arion D, Unger T, Lewis DA, Levitt P, Mirnics K (2007) Molecular evidence for increased expression of genes related to immune and chaperone function in the prefrontal cortex in schizophrenia. Biological psychiatry 62: 711–721.

[2] Barnes MR, Huxley-Jones J, Maycox PR, Lennon M, Thornber A, et al. (2011) Transcription and pathway analysis of the superior temporal cortex and anterior prefrontal cortex in schizophrenia. Journal of neuroscience research 89: 1218–1227.

[3] Glatt SJ, Everall IP, Kremen WS, Corbeil J, Šášik R, et al. (2005) Comparative gene expression analysis of blood and brain provides concurrent validation of selenbp1 up-regulation in schizophrenia. Proceedings of the National Academy of Sciences of the United States of America 102: 15533–15538.

[4] Maycox P, Kelly F, Taylor A, Bates S, Reid J, et al. (2009) Analysis of gene expression in two large schizophrenia cohorts identifies multiple changes associated with nerve terminal function. Molecular psychiatry 14: 1083–1094.

[5] Mistry M, Gillis J, Pavlidis P (2013) Genome-wide expression profiling of schizophrenia using a large combined cohort. Molecular psychiatry 18: 215–225.

[6] Pérez-Santiago J, Diez-Alarcia R, Callado LF, Zhang JX, Chana G, et al. (2012) A combined analysis of microarray gene expression studies of the human prefrontal cortex identifies genes implicated in schizophrenia. Journal of psychiatric research .

[7] Nakatani N, Hattori E, Ohnishi T, Dean B, Iwayama Y, et al. (2006) Genome-wide expression analysis detects eight genes with robust alterations specific to bipolar i disorder: relevance to neuronal network perturbation. Human molecular genetics 15: 1949–1962.

[8] Ryan M, Lockstone H, Huffaker S, Wayland M, Webster M, et al. (2006) Gene expression analysis of bipolar disorder reveals downregulation of the ubiquitin cycle and alterations in synaptic genes. Molecular psychiatry 11: 965–978.

[9] Seifuddin F, Pirooznia M, Judy JT, Goes FS, Potash JB, et al. (2013) Systematic review of genome-wide gene expression studies of bipolar disorder. BMC psychiatry 13: 213.

[10] Simunovic F, Yi M, Wang Y, Macey L, Brown LT, et al. (2009) Gene expression profiling of substantia nigra dopamine neurons: further insights into parkinson's disease pathology. Brain 132: 1795–1809.

[11] Zhang Y, James M, Middleton FA, Davis RL (2005) Transcriptional analysis of multiple brain regions in parkinson's disease supports the involvement of specific protein processing, energy metabolism, and signaling pathways, and suggests novel disease mechanisms. American Journal of Medical Genetics Part B: Neuropsychiatric Genetics 137: 5–16.
