# Supplementary material for: Method Designed to Respect Molecular Heterogeneity Can Profoundly Correct Present Data Interpretations for Genome-Wide Expression Analysis
Source: PLoS One. 2015 Mar 20;10(3):e0121154. doi: 10.1371/journal.pone.0121154 (PMC4368820; doi:10.1371/journal.pone.0121154)
Supplement: S1 Text — (PDF) [file pone.0121154.s026.pdf]

**Harvard Brain Tissue Resource Center:**

1. http://national_databank.mclean.harvard.edu/brainbank/Browse?action=cases&gid=1010
2. http://national_databank.mclean.harvard.edu/brainbank/Browse?action=cases&gid=1009
3. http://national_databank.mclean.harvard.edu/brainbank/Search?action=searchCases&collection=1001&group=-1&region=-1&diagnosis=-1&ethnicity=-1&gender=-1&causedeath=-1&agedeath=-1&pmi=-1&percentprobesets=-1&RNAratio=-1&3-5ratio_gapdh=-1&3-5ratio_b-actin=-1&searchCases=Search+Assays

**Stanley Medical Research Institute:**

1. https://www.stanleygenomics.org/stanley/standard/studyDetail.jsp?study_id=1
2. https://www.stanleygenomics.org/stanley/standard/studyDetail.jsp?study_id=2
3. https://www.stanleygenomics.org/stanley/standard/studyDetail.jsp?study_id=3
4. https://www.stanleygenomics.org/stanley/standard/studyDetail.jsp?study_id=5
5. https://www.stanleygenomics.org/stanley/standard/studyDetail.jsp?study_id=10
6. https://www.stanleygenomics.org/stanley/standard/studyDetail.jsp?study_id=14

**Gene Expression Omnibus:**

1. http://www.ncbi.nlm.nih.gov/geo/query/acc.cgi?acc=GSE10006
2. http://www.ncbi.nlm.nih.gov/geo/query/acc.cgi?acc=GSE10041
3. http://www.ncbi.nlm.nih.gov/geo/query/acc.cgi?acc=GSE10063
4. http://www.ncbi.nlm.nih.gov/geo/query/acc.cgi?acc=GSE10245
5. http://www.ncbi.nlm.nih.gov/geo/query/acc.cgi?acc=GSE10334
6. http://www.ncbi.nlm.nih.gov/geo/query/acc.cgi?acc=GSE10479
7. http://www.ncbi.nlm.nih.gov/geo/query/acc.cgi?acc=GSE10586
8. http://www.ncbi.nlm.nih.gov/geo/query/acc.cgi?acc=GSE10700
9. http://www.ncbi.nlm.nih.gov/geo/query/acc.cgi?acc=GSE10715
10. http://www.ncbi.nlm.nih.gov/geo/query/acc.cgi?acc=GSE10810
11. http://www.ncbi.nlm.nih.gov/geo/query/acc.cgi?acc=GSE10927
12. http://www.ncbi.nlm.nih.gov/geo/query/acc.cgi?acc=GSE11083
13. http://www.ncbi.nlm.nih.gov/geo/query/acc.cgi?acc=GSE11199
14. http://www.ncbi.nlm.nih.gov/geo/query/acc.cgi?acc=GSE11348
15. http://www.ncbi.nlm.nih.gov/geo/query/acc.cgi?acc=GSE11430
16. http://www.ncbi.nlm.nih.gov/geo/query/acc.cgi?acc=GSE11524
17. http://www.ncbi.nlm.nih.gov/geo/query/acc.cgi?acc=GSE11618
18. http://www.ncbi.nlm.nih.gov/geo/query/acc.cgi?acc=GSE11839
19. http://www.ncbi.nlm.nih.gov/geo/query/acc.cgi?acc=GSE11869

20. http://www.ncbi.nlm.nih.gov/geo/query/acc.cgi?acc=GSE11886
21. http://www.ncbi.nlm.nih.gov/geo/query/acc.cgi?acc=GSE11959
22. http://www.ncbi.nlm.nih.gov/geo/query/acc.cgi?acc=GSE11981
23. http://www.ncbi.nlm.nih.gov/geo/query/acc.cgi?acc=GSE12056
24. http://www.ncbi.nlm.nih.gov/geo/query/acc.cgi?acc=GSE12108
25. http://www.ncbi.nlm.nih.gov/geo/query/acc.cgi?acc=GSE12121
26. http://www.ncbi.nlm.nih.gov/geo/query/acc.cgi?acc=GSE12198
27. http://www.ncbi.nlm.nih.gov/geo/query/acc.cgi?acc=GSE12205
28. http://www.ncbi.nlm.nih.gov/geo/query/acc.cgi?acc=GSE12251
29. http://www.ncbi.nlm.nih.gov/geo/query/acc.cgi?acc=GSE12368
30. http://www.ncbi.nlm.nih.gov/geo/query/acc.cgi?acc=GSE12452
31. http://www.ncbi.nlm.nih.gov/geo/query/acc.cgi?acc=GSE12631
32. http://www.ncbi.nlm.nih.gov/geo/query/acc.cgi?acc=GSE12644
33. http://www.ncbi.nlm.nih.gov/geo/query/acc.cgi?acc=GSE12679
34. http://www.ncbi.nlm.nih.gov/geo/query/acc.cgi?acc=GSE12734
35. http://www.ncbi.nlm.nih.gov/geo/query/acc.cgi?acc=GSE12767
36. http://www.ncbi.nlm.nih.gov/geo/query/acc.cgi?acc=GSE12773
37. http://www.ncbi.nlm.nih.gov/geo/query/acc.cgi?acc=GSE12949
38. http://www.ncbi.nlm.nih.gov/geo/query/acc.cgi?acc=GSE13067
39. http://www.ncbi.nlm.nih.gov/geo/query/acc.cgi?acc=GSE13122
40. http://www.ncbi.nlm.nih.gov/geo/query/acc.cgi?acc=GSE13205
41. http://www.ncbi.nlm.nih.gov/geo/query/acc.cgi?acc=GSE13274
42. http://www.ncbi.nlm.nih.gov/geo/query/acc.cgi?acc=GSE13284
43. http://www.ncbi.nlm.nih.gov/geo/query/acc.cgi?acc=GSE13294
44. http://www.ncbi.nlm.nih.gov/geo/query/acc.cgi?acc=GSE13670
45. http://www.ncbi.nlm.nih.gov/geo/query/acc.cgi?acc=GSE13671
46. http://www.ncbi.nlm.nih.gov/geo/query/acc.cgi?acc=GSE13933
47. http://www.ncbi.nlm.nih.gov/geo/query/acc.cgi?acc=GSE13987
48. http://www.ncbi.nlm.nih.gov/geo/query/acc.cgi?acc=GSE14001
49. http://www.ncbi.nlm.nih.gov/geo/query/acc.cgi?acc=GSE14580
50. http://www.ncbi.nlm.nih.gov/geo/query/acc.cgi?acc=GSE14858
51. http://www.ncbi.nlm.nih.gov/geo/query/acc.cgi?acc=GSE14924
52. http://www.ncbi.nlm.nih.gov/geo/query/acc.cgi?acc=GSE14951
53. http://www.ncbi.nlm.nih.gov/geo/query/acc.cgi?acc=GSE14970
54. http://www.ncbi.nlm.nih.gov/geo/query/acc.cgi?acc=GSE14975
55. http://www.ncbi.nlm.nih.gov/geo/query/acc.cgi?acc=GSE15090
56. http://www.ncbi.nlm.nih.gov/geo/query/acc.cgi?acc=GSE15192
57. http://www.ncbi.nlm.nih.gov/geo/query/acc.cgi?acc=GSE15271

58. http://www.ncbi.nlm.nih.gov/geo/query/acc.cgi?acc=GSE15372
59. http://www.ncbi.nlm.nih.gov/geo/query/acc.cgi?acc=GSE18608
60. http://www.ncbi.nlm.nih.gov/geo/query/acc.cgi?acc=GSE19650
61. http://www.ncbi.nlm.nih.gov/geo/query/acc.cgi?acc=GSE19810
62. http://www.ncbi.nlm.nih.gov/geo/query/acc.cgi?acc=GSE19982
63. http://www.ncbi.nlm.nih.gov/geo/query/acc.cgi?acc=GSE20211
64. http://www.ncbi.nlm.nih.gov/geo/query/acc.cgi?acc=GSE20504
65. http://www.ncbi.nlm.nih.gov/geo/query/acc.cgi?acc=GSE20891
66. http://www.ncbi.nlm.nih.gov/geo/query/acc.cgi?acc=GSE20910
67. http://www.ncbi.nlm.nih.gov/geo/query/acc.cgi?acc=GSE21369
68. http://www.ncbi.nlm.nih.gov/geo/query/acc.cgi?acc=GSE21422
69. http://www.ncbi.nlm.nih.gov/geo/query/acc.cgi?acc=GSE21942
70. http://www.ncbi.nlm.nih.gov/geo/query/acc.cgi?acc=GSE22035
71. http://www.ncbi.nlm.nih.gov/geo/query/acc.cgi?acc=GSE22224
72. http://www.ncbi.nlm.nih.gov/geo/query/acc.cgi?acc=GSE22435
73. http://www.ncbi.nlm.nih.gov/geo/query/acc.cgi?acc=GSE22459
74. http://www.ncbi.nlm.nih.gov/geo/query/acc.cgi?acc=GSE22600
75. http://www.ncbi.nlm.nih.gov/geo/query/acc.cgi?acc=GSE22842
76. http://www.ncbi.nlm.nih.gov/geo/query/acc.cgi?acc=GSE22868
77. http://www.ncbi.nlm.nih.gov/geo/query/acc.cgi?acc=GSE23343
78. http://www.ncbi.nlm.nih.gov/geo/query/acc.cgi?acc=GSE23984
79. http://www.ncbi.nlm.nih.gov/geo/query/acc.cgi?acc=GSE23994
80. http://www.ncbi.nlm.nih.gov/geo/query/acc.cgi?acc=GSE24468
81. http://www.ncbi.nlm.nih.gov/geo/query/acc.cgi?acc=GSE24795
82. http://www.ncbi.nlm.nih.gov/geo/query/acc.cgi?acc=GSE24869
83. http://www.ncbi.nlm.nih.gov/geo/query/acc.cgi?acc=GSE25014
84. http://www.ncbi.nlm.nih.gov/geo/query/acc.cgi?acc=GSE25518
85. http://www.ncbi.nlm.nih.gov/geo/query/acc.cgi?acc=GSE25550
86. http://www.ncbi.nlm.nih.gov/geo/query/acc.cgi?acc=GSE26272
87. http://www.ncbi.nlm.nih.gov/geo/query/acc.cgi?acc=GSE26511
88. http://www.ncbi.nlm.nih.gov/geo/query/acc.cgi?acc=GSE26526
89. http://www.ncbi.nlm.nih.gov/geo/query/acc.cgi?acc=GSE26725
90. http://www.ncbi.nlm.nih.gov/geo/query/acc.cgi?acc=GSE26966
91. http://www.ncbi.nlm.nih.gov/geo/query/acc.cgi?acc=GSE27187
92. http://www.ncbi.nlm.nih.gov/geo/query/acc.cgi?acc=GSE27390
93. http://www.ncbi.nlm.nih.gov/geo/query/acc.cgi?acc=GSE27659
94. http://www.ncbi.nlm.nih.gov/geo/query/acc.cgi?acc=GSE28750
95. http://www.ncbi.nlm.nih.gov/geo/query/acc.cgi?acc=GSE30153

96. http://www.ncbi.nlm.nih.gov/geo/query/acc.cgi?acc=GSE30201
97. http://www.ncbi.nlm.nih.gov/geo/query/acc.cgi?acc=GSE30355
98. http://www.ncbi.nlm.nih.gov/geo/query/acc.cgi?acc=GSE30784
99. http://www.ncbi.nlm.nih.gov/geo/query/acc.cgi?acc=GSE31177
100. http://www.ncbi.nlm.nih.gov/geo/query/acc.cgi?acc=GSE31215
101. http://www.ncbi.nlm.nih.gov/geo/query/acc.cgi?acc=GSE31773
102. http://www.ncbi.nlm.nih.gov/geo/query/acc.cgi?acc=GSE32057
103. http://www.ncbi.nlm.nih.gov/geo/query/acc.cgi?acc=GSE32178
104. http://www.ncbi.nlm.nih.gov/geo/query/acc.cgi?acc=GSE32473
105. http://www.ncbi.nlm.nih.gov/geo/query/acc.cgi?acc=GSE32701
106. http://www.ncbi.nlm.nih.gov/geo/query/acc.cgi?acc=GSE32719
107. http://www.ncbi.nlm.nih.gov/geo/query/acc.cgi?acc=GSE3292
108. http://www.ncbi.nlm.nih.gov/geo/query/acc.cgi?acc=GSE32924
109. http://www.ncbi.nlm.nih.gov/geo/query/acc.cgi?acc=GSE33316
110. http://www.ncbi.nlm.nih.gov/geo/query/acc.cgi?acc=GSE4107
111. http://www.ncbi.nlm.nih.gov/geo/query/acc.cgi?acc=GSE4182
112. http://www.ncbi.nlm.nih.gov/geo/query/acc.cgi?acc=GSE4183
113. http://www.ncbi.nlm.nih.gov/geo/query/acc.cgi?acc=GSE4290
114. http://www.ncbi.nlm.nih.gov/geo/query/acc.cgi?acc=GSE4302
115. http://www.ncbi.nlm.nih.gov/geo/query/acc.cgi?acc=GSE4488
116. http://www.ncbi.nlm.nih.gov/geo/query/acc.cgi?acc=GSE4498
117. http://www.ncbi.nlm.nih.gov/geo/query/acc.cgi?acc=GSE4567
118. http://www.ncbi.nlm.nih.gov/geo/query/acc.cgi?acc=GSE5058
119. http://www.ncbi.nlm.nih.gov/geo/query/acc.cgi?acc=GSE5281
120. http://www.ncbi.nlm.nih.gov/geo/query/acc.cgi?acc=GSE5563
121. http://www.ncbi.nlm.nih.gov/geo/query/acc.cgi?acc=GSE5764
122. http://www.ncbi.nlm.nih.gov/geo/query/acc.cgi?acc=GSE5850
123. http://www.ncbi.nlm.nih.gov/geo/query/acc.cgi?acc=GSE6054
124. http://www.ncbi.nlm.nih.gov/geo/query/acc.cgi?acc=GSE6281
125. http://www.ncbi.nlm.nih.gov/geo/query/acc.cgi?acc=GSE6364
126. http://www.ncbi.nlm.nih.gov/geo/query/acc.cgi?acc=GSE6519
127. http://www.ncbi.nlm.nih.gov/geo/query/acc.cgi?acc=GSE6575
128. http://www.ncbi.nlm.nih.gov/geo/query/acc.cgi?acc=GSE6798
129. http://www.ncbi.nlm.nih.gov/geo/query/acc.cgi?acc=GSE6872
130. http://www.ncbi.nlm.nih.gov/geo/query/acc.cgi?acc=GSE6885
131. http://www.ncbi.nlm.nih.gov/geo/query/acc.cgi?acc=GSE6962
132. http://www.ncbi.nlm.nih.gov/geo/query/acc.cgi?acc=GSE7014
133. http://www.ncbi.nlm.nih.gov/geo/query/acc.cgi?acc=GSE7023

134. http://www.ncbi.nlm.nih.gov/geo/query/acc.cgi?acc=GSE7116
135. http://www.ncbi.nlm.nih.gov/geo/query/acc.cgi?acc=GSE7161
136. http://www.ncbi.nlm.nih.gov/geo/query/acc.cgi?acc=GSE7305
137. http://www.ncbi.nlm.nih.gov/geo/query/acc.cgi?acc=GSE7462
138. http://www.ncbi.nlm.nih.gov/geo/query/acc.cgi?acc=GSE7510
139. http://www.ncbi.nlm.nih.gov/geo/query/acc.cgi?acc=GSE7513
140. http://www.ncbi.nlm.nih.gov/geo/query/acc.cgi?acc=GSE7515
141. http://www.ncbi.nlm.nih.gov/geo/query/acc.cgi?acc=GSE7554
142. http://www.ncbi.nlm.nih.gov/geo/query/acc.cgi?acc=GSE7562
143. http://www.ncbi.nlm.nih.gov/geo/query/acc.cgi?acc=GSE7568
144. http://www.ncbi.nlm.nih.gov/geo/query/acc.cgi?acc=GSE7621
145. http://www.ncbi.nlm.nih.gov/geo/query/acc.cgi?acc=GSE7753
146. http://www.ncbi.nlm.nih.gov/geo/query/acc.cgi?acc=GSE7788
147. http://www.ncbi.nlm.nih.gov/geo/query/acc.cgi?acc=GSE7807
148. http://www.ncbi.nlm.nih.gov/geo/query/acc.cgi?acc=GSE7846
149. http://www.ncbi.nlm.nih.gov/geo/query/acc.cgi?acc=GSE7869
150. http://www.ncbi.nlm.nih.gov/geo/query/acc.cgi?acc=GSE7890
151. http://www.ncbi.nlm.nih.gov/geo/query/acc.cgi?acc=GSE8762
152. http://www.ncbi.nlm.nih.gov/geo/query/acc.cgi?acc=GSE8823
153. http://www.ncbi.nlm.nih.gov/geo/query/acc.cgi?acc=GSE9150
154. http://www.ncbi.nlm.nih.gov/geo/query/acc.cgi?acc=GSE9212
155. http://www.ncbi.nlm.nih.gov/geo/query/acc.cgi?acc=GSE9250
156. http://www.ncbi.nlm.nih.gov/geo/query/acc.cgi?acc=GSE9264
157. http://www.ncbi.nlm.nih.gov/geo/query/acc.cgi?acc=GSE21935
158. http://www.ncbi.nlm.nih.gov/geo/query/acc.cgi?acc=GSE17612
159. http://www.ncbi.nlm.nih.gov/geo/query/acc.cgi?acc=GSE12649
160. http://www.ncbi.nlm.nih.gov/geo/query/acc.cgi?acc=GSE12654
161. http://www.ncbi.nlm.nih.gov/geo/query/acc.cgi?acc=GSE5388
162. http://www.ncbi.nlm.nih.gov/geo/query/acc.cgi?acc=GSE5389
163. http://www.ncbi.nlm.nih.gov/geo/query/acc.cgi?acc=GSE19587
164. http://www.ncbi.nlm.nih.gov/geo/query/acc.cgi?acc=GSE20168
165. http://www.ncbi.nlm.nih.gov/geo/query/acc.cgi?acc=GSE20291
166. http://www.ncbi.nlm.nih.gov/geo/query/acc.cgi?acc=GSE20292
167. http://www.ncbi.nlm.nih.gov/geo/query/acc.cgi?acc=GSE20141
168. http://www.ncbi.nlm.nih.gov/geo/query/acc.cgi?acc=GSE20163
